# Discovery of increased epidermal DNAH10 expression after regeneration of dermis in a randomized with-in person trial — reflections on psoriatic inflammation

**DOI:** 10.1038/s41598-019-53874-z

**Published:** 2019-12-13

**Authors:** Heli Lagus, Mariliis Klaas, Susanna Juteau, Outi Elomaa, Juha Kere, Jyrki Vuola, Viljar Jaks, Esko Kankuri

**Affiliations:** 1 Helsinki Burn Centre, Department of Plastic Surgery, Helsinki University Hospital, University of Helsinki, Helsinki, Finland; 2 Department of Cell Biology, Institute of Molecular and Cell Biology, University of Tartu, Tartu, Estonia; 3 Department of Pathology, Haartman Institute, University of Helsinki and HUSLAB, Helsinki, Finland; 4 Folkhälsan Research Center, Helsinki, Finland; 5 Department of Medical and Clinical Genetics, Medicum and Research Programs Unit, Molecular Neurology, University of Helsinki, Helsinki, Finland; 6 Department of Biosciences and Nutrition, Karolinska Institutet, Solna, Sweden; 7 Faculty of Medicine, Department of Pharmacology, University of Helsinki, Helsinki, Finland

**Keywords:** Proteomic analysis, Outcomes research, Translational research

## Abstract

Because molecular memories of past inflammatory events can persist in epidermal cells, we evaluated the long-term epidermal protein expression landscapes after dermal regeneration and in psoriatic inflammation. We first characterized the effects of two dermal regeneration strategies on transplants of indicator split-thickness skin grafts (STSGs) in ten adult patients with deep burns covering more than 20% of their body surface area. After fascial excision, three adjacent areas within the wound were randomized to receive a permanent dermal matrix, a temporary granulation-tissue-inducing dressing or no dermal component as control. Control areas were covered with STSG immediately, and treated areas after two-weeks of dermis formation. Epidermis-dermis-targeted proteomics of one-year-follow-up samples were performed for protein expression profiling. Epidermal expression of axonemal dynein heavy chain 10 (DNAH10) was increased 20-fold in samples having had regenerating dermis vs control. Given the dermal inflammatory component found in our dermal regeneration samples as well as in early psoriatic lesions, we hypothesized that DNAH10 protein expression also would be affected in psoriatic skin samples. We discovered increased DNAH10 expression in inflammatory lesions when compared to unaffected skin. Our results associate DNAH10 expression with cell proliferation and inflammation as well as with the epidermal memory resulting from the previous regenerative signals of dermis. This study (ISRCTN14499986) was funded by the Finnish Ministry of Defense and by government subsidies for medical research.

## Introduction

Cellular and molecular interactions in the skin are crucial for preserving structure and functionality of the epidermis, the skin’s outer boundary to the external world^1^. Epidermal cells undergo a coordinated process of differentiation and apoptosis as they migrate to their demise, from the basement membrane separating epidermis from the underlying dermis^2–4^. The basement membrane anchors basal keratinocytes and regulates cell trafficking and diffusion of bioactive molecules^5^. The dermis, rich in extracellular matrix, nerves, and vasculature, nurtures the epidermis from below and produces critical paracrine signals to assist maintenance and regulation of epidermal structure and homeostasis^6–9^.

Signals and pathways that mediate regulating epidermal-dermal interactions include members of Wnt^10^ and sonic hedgehog^11^ pathways, several growth factors, and cytokines and their receptors^12–14^, as well as cell-cell juxtacrine signaling by, for example, the Notch pathway^15^. Epidermal cells utilize highly conserved membranous structures, such as the primary cilia, to translate these signals for control of critical cellular responses e.g., proliferation, migration, and differentiation^16–19^. Especially during wound healing, signaling between the epidermal and dermal compartments is highly activated^20–22^. After wound healing, memory of the wound persists as molecular level changes in epidermal T-cells^23,24^ and in epithelial stem cells^25,26^. Very little is known about epidermal responses and molecular memory after regeneration of the entire dermis in humans.

**Figure 1:**
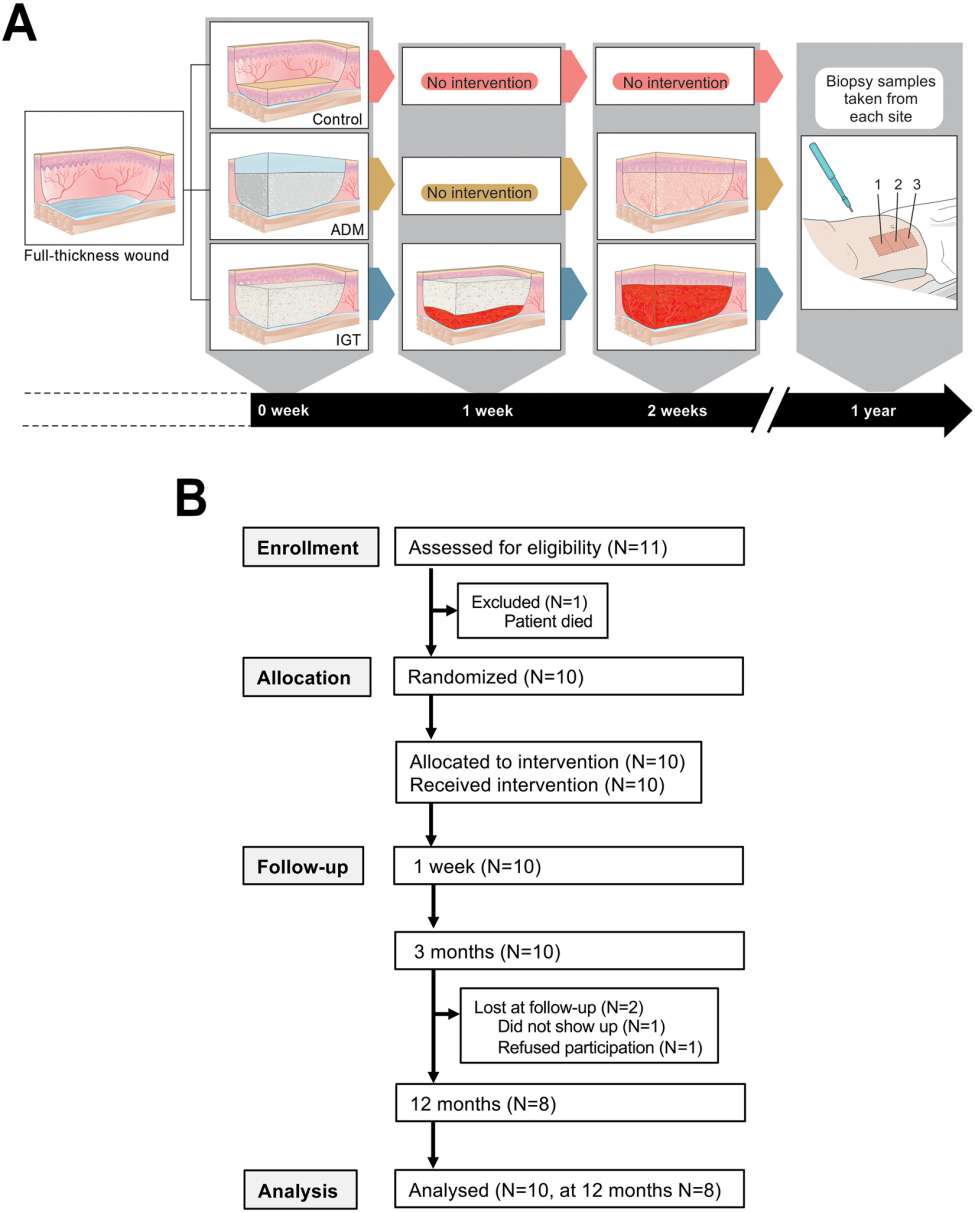
(**A**) Timeline of the treatment protocol. 0 week: After excision to fascial level the wound bed of the C group (the top row) was covered with STSG, ADM group (the middle row) with dermal matrix Integra and IGT group (the bottom row) with a temporary dressing of viscose cellulose sponge Cellonex. 1 week: IGT group: cellulose sponge was changed to a new one. 2 weeks: ADM group: Silicone layer of dermal matrix was replaced with a STSG, IGT group: cellulose sponge was replaced with STSG. 1 year: Punch biopsies of each study section were gathered. Illustration made by Sole Lätti. (**B**) CONSORT flow diagram

Skin regeneration follows a path similar to wound healing beginning with the formation of granulation tissue—an initial inflammatory, vascularized, and highly proliferative dermal template—to support the epidermis. Here, we employed a clinical setting to investigate long-term epidermal reactivity in response to complete dermal regeneration (Fig. 1). We extend our results to a pathological perspective of dermal inflammation in skin samples collected from lesional and non-lesional sites of psoriatic skin, as well as from healthy control skin^27,28^.

## Results

### Proteins identified in skin grafts

**Figure 2:**
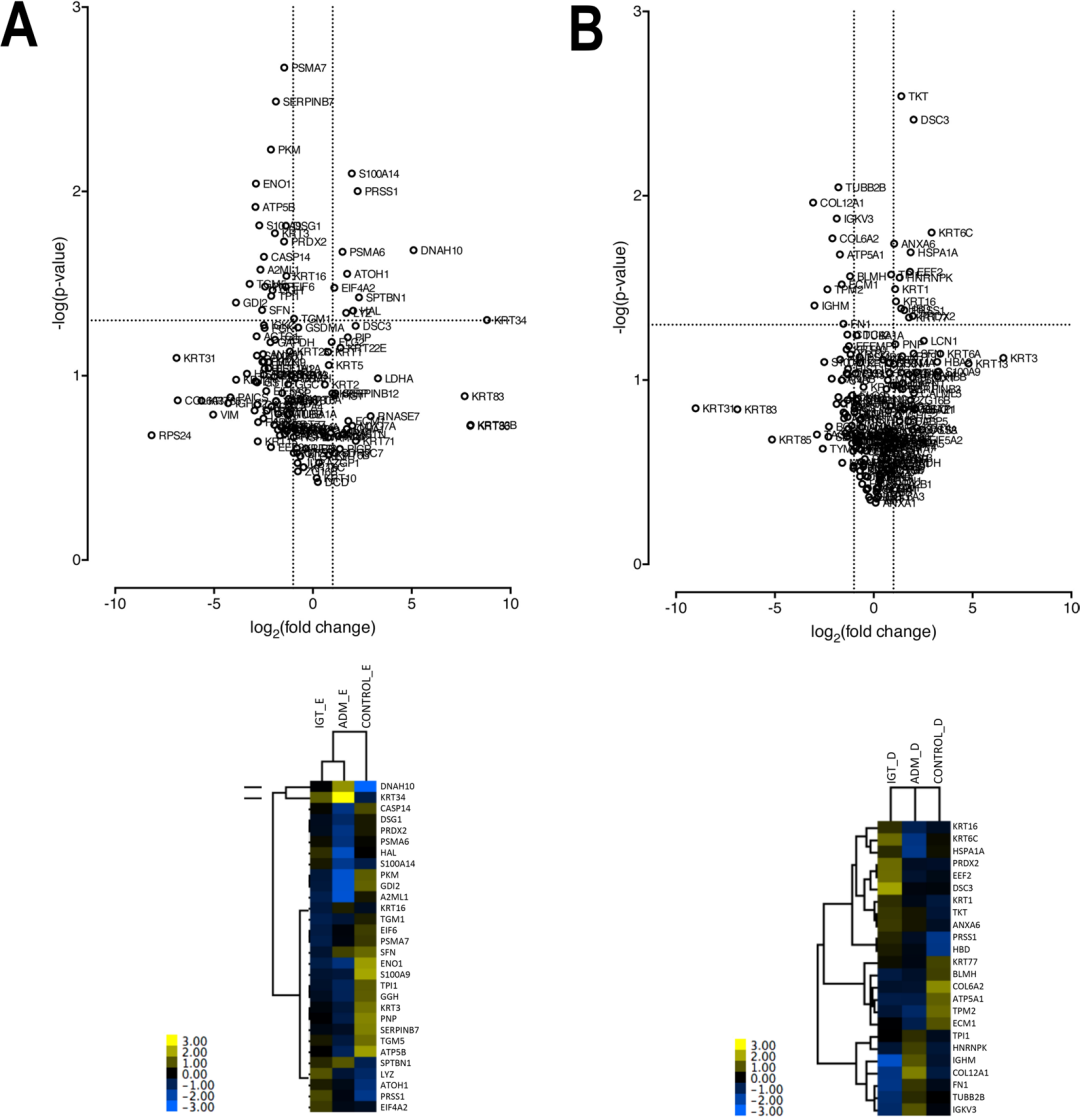
Volcano plots (upper panels) and heat maps (lower panels) of proteins in epidermis (**A**) and dermis (**B**). Values selected separately for highest significance (y-axis/−log, p-value) and greatest fold change (x-axis/log2) across all comparisons between groups. Horizontal dotted line, p = 0.05. Vertical dotted lines, fold change = ±2. Lower panels heat maps show proteins expressed significantly differently (p > 0.05) in these three different treatment groups (IGT, ADM, and control).

We identified 481 proteins by untargeted, non-labelled proteomics of epidermal and dermal samples (The mass spectrometry proteomics data are available via the PRIDE^29^ partner repository with dataset identifier PXD010852; www.proteomexchange.org). All samples were subjected to blind clustering analysis to validate the ability of the microdissection procedure to distinguish between epidermis and dermis (Supplementary Table S1A n = 131 and B n = 181; with predominant proteins both in epidermis and dermis and Fig. S1 heat maps of proteins of all groups (Fig. S1A) and of epidermal and dermal proteins (Fig. S1B)). The volcano plots of proteins detectable across samples in epidermis and dermis are shown in Fig. 2 (upper panels). We found 30 proteins in epidermal and 24 proteins in dermal samples that were differentially expressed across treatment groups. The heat maps of these differentially expressed proteins (DEPs) are shown in the lower panels of Fig. 2. Of all proteins identified, 12% were significantly differently expressed between the treatment sites.

### Early granulation tissue and dermal substitute increased the motor protein DNAH10 long-term expression in skin-graft keratinocytes

**Figure 3:**
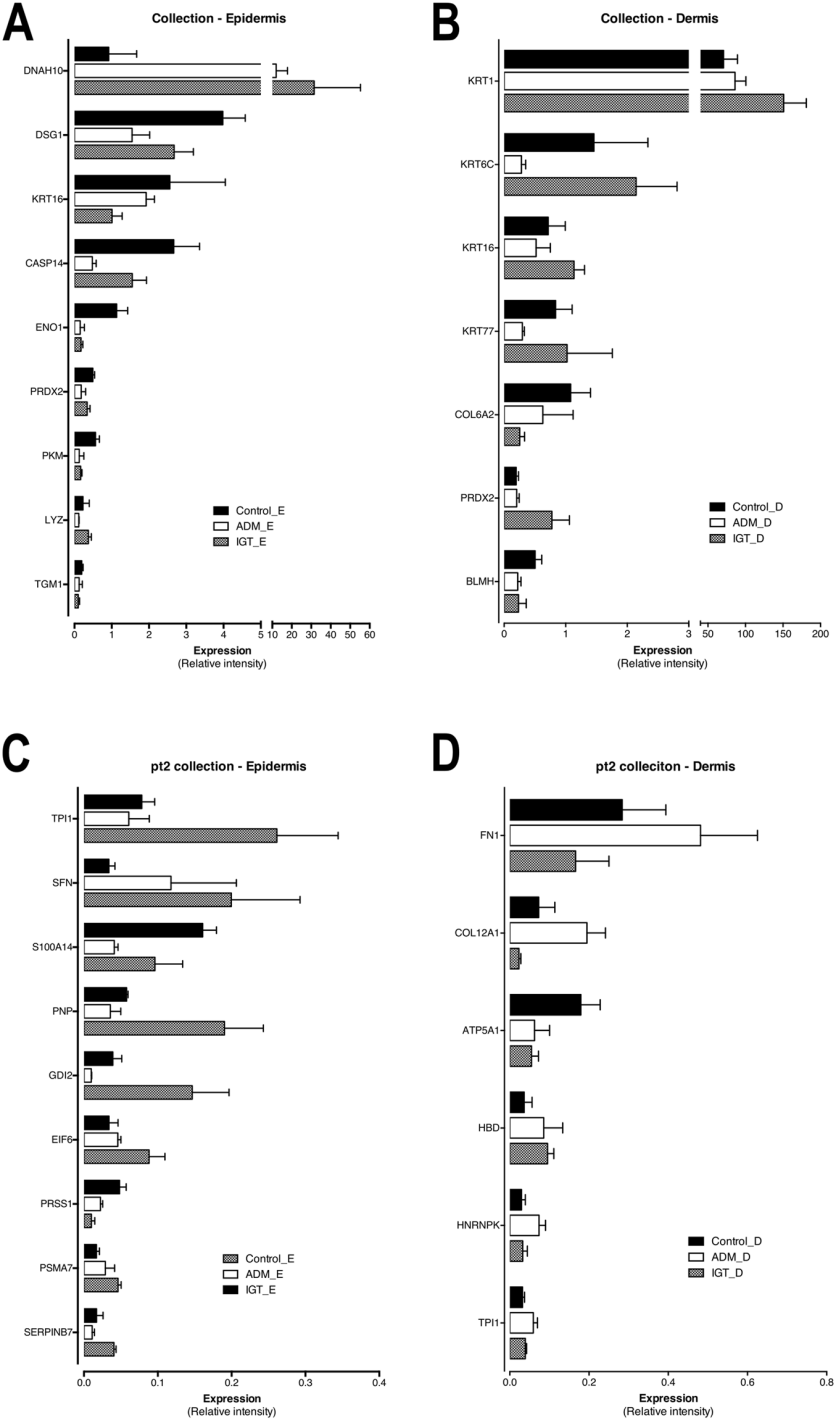
Differentially expressed proteins in comparison between the test-groups measured in relative intensity in mass-spectrometry of all proteins identified. Panels A and C show proteins with quantified abundant expression in epidermis and dermis respectively. Panels B and D show proteins with quantified low expression levels in epidermis and dermis respectively.

The most striking significant difference among epidermal proteins was the expression of DNAH10 protein, with a 34-fold difference between the control group with the lowest and the cellulose-sponge-treated group with induced granulation tissue (IGT group) with the highest expression values. Although the expression of an acidic (type I) cuticular hair keratin 34, KRT34, demonstrated an immense 448-fold increase in artificial dermal matrix (ADM)-treated samples compared to control, and a 119-fold difference between the ADM and IGT groups, only the 14-fold change in the IGT group vs control group attained statistical significance, possibly due to small sample size in ADM group. Caspase-14 (CASP14), a marker for keratinocyte differentiation, showed decreased expression in groups with dermal manipulation compared to the control group. The most differently expressed proteins in epidermis are shown in Fig. 3 panels A and C, and the most differently expressed proteins in dermis in panels B and D (Supplementary Fig. S2 shows functions and diseases associated with these proteins, and Table S2 DEPs).

**Figure 4:**
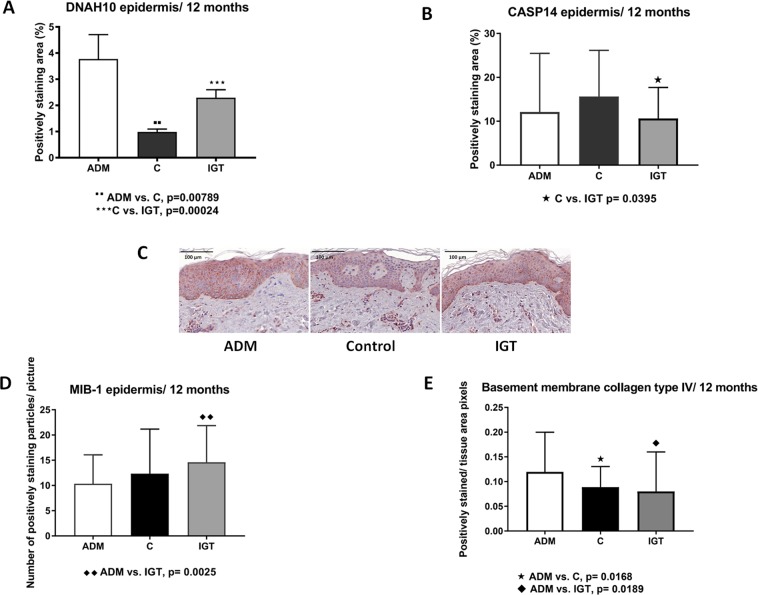
Immunohistochemistry of STSGs 1 year after operation. (**A**) Diagram of DNAH10 staining in control group (**C**), with no additional treatment of wound bed and in manipulated wound beds ADM and IGT. (**B**) Pictures of STSGs of DNAH10- staining: left on ADM, in the middle control and on right on IGT. (**C**) CASP14 is a marker for differentiation of keratinocytes. (**D**) Proliferation marker MIB-1 showed positively stained particles corresponding cell nuclei/picture. The slides were scanned with the 20x air objective, images were captured with the magnification 65 (±1.4)x scanned slides. (**E**) Basement membrane component type IV collagen staining. In all diagrams error bars present mean +/−SEM.

In order to validate the findings from the proteomic data, the corresponding tissue sections were immunohistochemically stained using anti-DNAH10 and anti-CASP14 antibodies. Although the differences seen with immunohistochemistry were less distinct than in the proteomic analyses, the control group similarly showed the least staining for DNAH10 with significant differences compared to the other treatment groups (Fig. 4).

In samples receiving dermal components, DNAH10 staining demonstrated a tendency for an aberrant or biphasic cytosolic pattern with staining observed in the basal layer as well as in the upper layers (stratum granulosum), while staining remained less intensive for DNAH10 in the thickest layer, stratum spinosum. In the control samples, the DNAH10 staining intensity remained at low levels and a similar staining pattern to that seen in the samples treated with dermal component manipulation was not observed. For CASP14, the control group showed most staining. This corroborates the difference found in the proteomics analysis.

To further investigate the role of DNAH10 and its association to different biological processes, such as proliferation and formation of basement membrane, immunohistochemical staining was performed to detect proliferation using antibodies against the Ki-67 (clone MIB-1) and basement membrane protein collagen type IV antigens. An inverse relationship between DNAH10 and MIB-1 was detected. Staining for collagen type IV also showed an inverse relationship to MIB-1 staining (Fig. 4).

### DNAH10 is associated with epithelial cell proliferation and differentiation

We performed pathway and network analysis for the epidermal DEPs using Ingenuity Pathway Analysis (IPA) software. Proteins with increased expression across comparisons, including DNAH10, were associated with pathways such as proliferation of epithelial cells, differentiation of hair cells, and epithelial barrier formation (Supplementary Fig. S3). The Diseases and Functions-category listings by IPA that demonstrated the strongest associations with the DEPs are shown in Supplementary Fig. S2 and Supplementary Table S3.

The analysis of the DNAH10 promoter region to predict transcription factor (TF) binding sites was performed using TRANSFAC (Supplementary Table S4). The transcription factors AP-2, KLF-6, and ZF-5 had most binding sites within the promoter. All three TFs regulate cell proliferation and differentiation. The DNAH10 gene on chromosome 12 has ten splice variants, six of which yield protein products (Supplementary Table S5). The transcripts DNAH10-210 and DNAH10-201 produce the large dynein proteins with molecular weights of 500 kDa, whereas the transcript variants DNAH10-205, −207, and −209 yield smaller proteins (molecular weights ranging from 35 to 115 kDa). Our proteomics profiling identified a unique peptide sequence “LESIFIGGDIRSQLPEEAKKFDNIDKVF” for the differentially expressed DNAH10. This sequence is present in the high molecular weight protein products of DNAH10, whereas only the DNAH10-205 variant of transcripts yielding low molecular weight proteins includes this sequence (Supplementary Table S6).

### Mitomycin C-treatment increases the expression of DNAH10 in cultured primary keratinocytes

**Figure 5:**
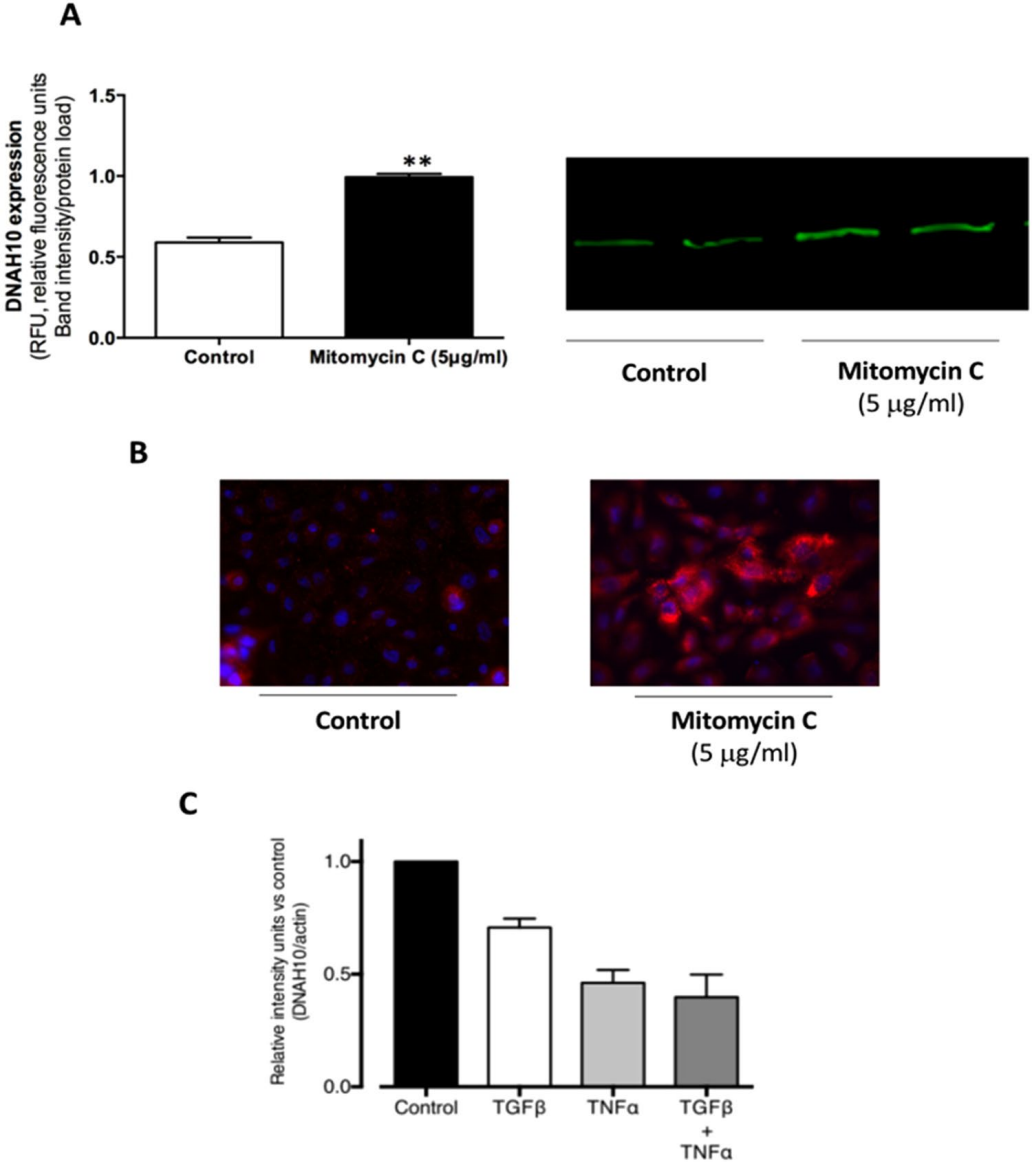
Effect of induction of cell cycle arrest on DNAH10 expression in keratinocytes. (**A**,**B**) Primary keratinocyte cell cultures treated with or without mitomycin C (5 μg/ml) for 3 hours. After 24-hour incubation in culture media DNAH10 expression in keratinocytes shown both in Western blot (**A**) and immunofluorence cytochemistry (**B**) after 24-hour incubation in culture media shown in 200 x magnification power. (**C**) Keratinocyte culture stimulation with TNFα, TGF-β1, and both together for 24 hours shown in relative intensity of DNAH10 expression in Western blot. In Western blots n = 3–4.

To further examine the association between proliferation and DNAH10 expression, cultured early passage (P3) primary keratinocytes were treated with mitomycin C (5 µg/ml) for three hours and the expression of DNAH10 was evaluated after 24 hours with Western blot (Fig. 5A) and immunofluorescence staining (Fig. 5B). Mitomycin C is an antineoplastic agent that inhibits DNA synthesis in various mammalian cells. Both Western blot and immunocytochemistry showed high DNAH10 expression in the mitomycin C treatment group vs control.

### Treatments with TGF-β1 and TNFα decrease DNAH10 expression in cultured primary keratinocytes

The protein expression of DNAH10 was assessed by Western blot analysis after stimulation of primary keratinocytes (P4) with inflammatory cytokine TNFα or growth factor TGF-β1 and their combination (Fig. 5C). Both stimulants are multitasking proteins known to take part in wound healing, affect keratinocyte proliferation, and cause cell-cycle arrest. Treatment with TNFα, TGF-β1, and their combination down regulated the expression of DNAH10 in keratinocytes after a 24-h-stimulation. DNAH10 expression levels were as follows: control (100%) > TGF-β1 (71%, SD 6.9%) > TNFα (46%, SD 9.9%) > combination of TGF-β1 and TNFα (40%, SD 17.0%).

### DNAH10 protein expression is increased in psoriatic lesions

**Figure 6:**
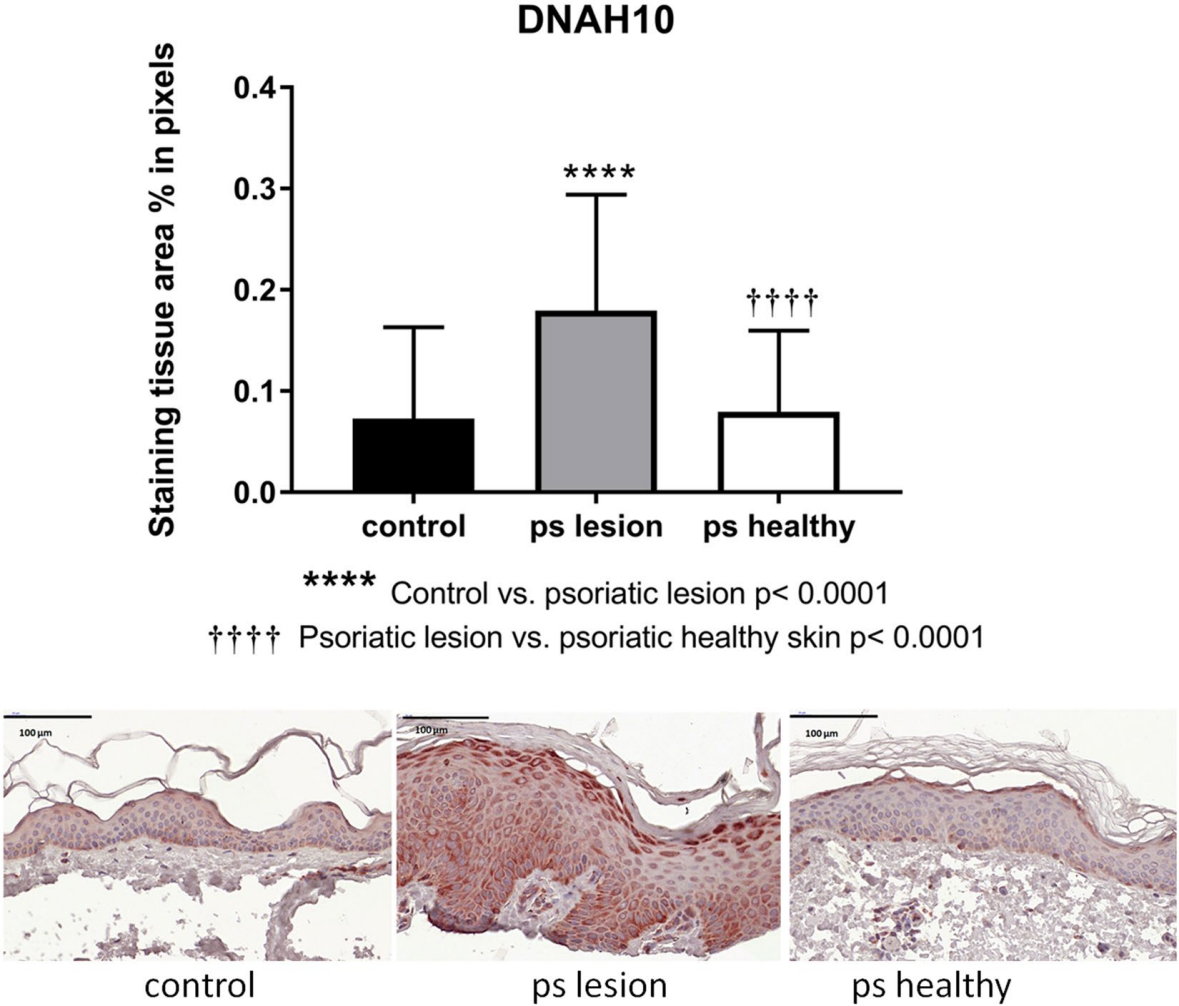
DNAH10 immunohistochemistry of STSGs of psoriasis patients, lesional and non-lesional samples, compared to the samples of healthy individuals. The slides were scanned with 20x air objective and images were captured with the magnification 65 (±1.4)x scanned slides.

Using IPA, we associated the increased expression of DNAH10 in STSGs of dermis-manipulated groups vs control, with biological networks relating to proliferation, differentiation, and barrier function. Moreover, we found DNAH10 protein expression to be regulated by cytokine stimulation in primary human keratinocytes. Based on these results, we hypothesized that expression of DNAH10 would be affected in active psoriatic epidermis characterized by early dermal inflammation, keratinocyte hyperproliferation, and defective epidermal barrier function^30–32^. Moreover, for our previous work on psoriasis and keratinocytes^27,28,33^ we had collected specifically thin STSG samples from both active inflamed and normal skin areas. To investigate whether DNAH10 would be differently present or distributed in normal skin, in samples of healthy controls compared to those of both lesional and non-lesional skin of psoriasis patients, the samples were immunohistochemically stained with anti-DNAH10 antibody. Expression of DNAH10 in lesional psoriatic samples was significantly higher than in either healthy control samples or non-lesional samples of psoriatic patients (Fig. 6).

The samples from healthy controls without psoriasis tended to demonstrate a similar cytosolic epithelial staining pattern as that observed in the samples receiving additional dermal components (Fig. 4C). A similar pattern was also observed in the psoriasis patient’s non-lesional epidermis. However, DNAH10 staining in the epidermis of the lesional skin showed less pronounced patterning, suggesting that psoriasis-associated alterations may disrupt DNAH10 expression patterning.

We then reviewed previous RNA sequencing data of psoriatic skin samples^28,34,35^. This review demonstrated that axonemal heavy chain dynein genes, including DNAH10, were not among the differentially expressed genes when comparing psoriatic lesional and non-lesional thin skin grafts. Down regulation was seen, however, in the lesional samples when analyzed from the full thickness skin samples including dermis. However, several other dynein genes as well as kinesin genes showed upregulation in lesional skin when compared to non-lesional samples (Supplementary Table S7).

## Discussion

Dermal signals and support are crucial for function, maturation, maintenance, and repair of the overlaying epidermal protective barrier layer. Here we utilized a clinical setup with three dermal compartments, on top of which autologous mainly epidermis-containing indicator tissues were transplanted. The sites were randomized to receive no additional dermal support, a permanent acellular dermal substitute, or a temporary dressing of viscose cellulose to induce granulation tissue formation. At one-year follow-up the gross clinical appearances of the treatment sites were equal^36^, but we observed specific early differences between treatment sites^37^. At one-week follow-up, dermal cells in both the granulation tissue and artificial dermal template proliferated actively, while such proliferative activity was only observed after three months at sites without additional dermal support^37^. In one-year follow-up samples, 12% of the proteins identified were differently expressed between treatment sites. We discovered greatly increased expression of a dynein protein, DNAH10, in epidermis predisposed to an active dermal component. There was also an evident trend for increased expression of KRT34 in both groups receiving dermal pretreatment compared to the control group, suggesting that dermal pretreatment may promote hair follicle maintenance^38^.

DNAH10 expression has been found in cilia and flagella^39^. Dyneins are multisubunit motor proteins that are associated with processes including microtubule transport, axonemal and ciliary signaling^39^, as well as chemosensory perception^40^ and cell migration^41^. DNAH proteins have also been linked to various cancers^42^. Axonemal dyneins, associated with the axonemes of primary cilia, are found in most human cells during G0/G1 and at the beginning of S phase, mainly in differentiated cells or in resting stem cells^40^. The primary cilium is resorbed in cells that re-enter the cell cycle and grows again on each daughter cell during the quiescent period^40^. Primary cilia transduce juxta- and paracrine signals to modulate key processes like cell survival, proliferation, migration, differentiation, and innate immunity. They mediate signals from various cascades and associate with pathways including the TGF-β pathway as well as the Hedgehog, Wnt, Notch, and Hippo signaling pathways^43,44^.

We demonstrated that the expression of DNAH10 was affected by mitomycin C, a DNA synthesis and cell-cycle inhibitor, in early passage primary cultures of normal human keratinocytes. Intriguingly, we observed increased DNAH10 staining overnight after the cells were transiently treated with the cell proliferation inhibitor mitomycin C. We also showed that treatment with TNFα, TGF-β1, and their combination led to a decrease in DNAH10 expression in cultured primary keratinocytes. Especially in the skin, TNFα and TGF-β1 have a pro-inflammatory role^45,46^. TNFα may sensitize cells to additional stimuli that determine whether the keratinocytes will undergo apoptosis, differentiation, or proliferation^47^. Thus, the cytokines’ suppressive action on DNAH10 expression in normal keratinocytes and the increased expression of DNAH10 in epidermis of psoriasis lesions suggest differential regulation of DNAH10 expression in normal keratinocytes and psoriatic epidermis, or even a dysregulation in psoriatic epidermis. Future elucidation of the mechanism of DNAH10 expressional regulation can be expected to unravel physiological and pathological, as well as acute and delayed expression differences, and possibly to unveil novel anti-inflammatory and anti-aging therapeutic targets.

Our findings demonstrated long-term increased expression of epidermal DNAH10 protein triggered by dermal signaling in a healing wound. We associated DNAH10 expression with cell proliferation, migration, and differentiation of keratinocytes, as well as inflammation. Because psoriasis, a hyperproliferative, inflammatory skin disorder with defects in epidermal differentiation and barrier function^29,46^ shows similar biological pathway associations, we hypothesized that the expression of epidermal DNAH10 would be differentially regulated as compared to normal or non-lesional skin. In the early stages of psoriatic plaques, dermal involvement is dominant, including immune-cell infiltration and production of TNFα as one of the main executor cytokines in psoriasis. TNFα regulates not only immune and inflammatory responses, but also tissue remodeling, cell motility, cell cycle, and apoptosis^47^. We discovered a significantly increased expression of DNAH10 in the lesional epidermis as compared to non-lesional epidermis or to epidermis of non-psoriatic patients. The tendency to aberrant biphasic or dual pattern of DNAH10 staining across the depth of the epidermis as observed in normal control epidermis, psoriatic epidermis, and during wound healing, suggests that DNAH10 expression is activated at specific times during keratinocyte differentiation. DNAH10 expression may associate with the progress of keratinocytes through epidermal layers during the epidermal differentiation process. The expression of axonemal dyneins has already been linked to cell differentiation and correlated with the appearance of a ciliated phenotype^48^. The results of this study highlight the need to elucidate the roles of primary cilia and the axoneme in the epidermis and the functional role of the axonemal dyneins during epidermal differentiation. These findings will increase our understanding of the intricate signaling networks and signaling pathways orchestrating the formation and integrity of the skin. Less intense staining in the stratum spinosum upon loss of cell-matrix contacts, followed by reinstated DNAH10 expression in the upper layers with reinforced cell-cell contacts, suggests the association of DNAH10 expression with processes and molecules especially related to the maintenance of cell contacts and adherence. Cell-cell and cell-matrix contacts are critically important in communication between homotypic and heterotypic cells, as well as in communication between the cells and their microenvironment. Cell-cell contacts, e.g. tight junctions in stratum granulosum and corneodesmosomes in stratum corneum, contribute to the formation of physical barriers crucial for the protective barrier function. Interestingly, tight junctions start to assemble only in granular cells, not in the spinous layer where the DNAH10 expression is lower^49^.

The induction of DNAH10 protein expression remained undetected at the mRNA level in previous studies based on RNA sequencing, suggesting that DNAH10 expression is controlled at a post-translational level. The size of the DNAH10 protein (515 kDa) can either directly or indirectly contribute to slow cycling and expressional changes of the protein. Moreover, we identified here by mass-spectrometry a unique DNAH10 peptide which could be encoded by a shorter transcript variant DNAH10-205. Interestingly this transcript is subject to nonsense-mediated decay (NMD)^50–52^ suggesting its escape and a contribution of the NMD pathway to DNAH10 or DNAH10-205 expression in the long-term memory response. The post-translational modification may regulate the expression pattern of DNAH10, similarly as diverse post-translational modifications of tubulin control the specialization of microtubules. This biological regulation known as the tubulin code^53^ may well have an analogous “dynein code” that regulates the dynein protein’s function and stability. Deciphering the enigma of post-translational “dynein code”, as well as the transcriptional and post-transcriptional events in epidermal activation and differentiation (e.g., transcripts escaping NMD^51,54^, could yield intriguing new insights into the physiological and pathological aspects of epithelial-mesenchymal interactions.

We also analyzed the promoter sequence of *DNAH10*^55^ using the TRANSFAC resource, and found binding sites to transcription factors, such as AP-2, Kruppel like factor 6 (KLF-6), and ZF5 (ZBTB14) that generally regulate cell proliferation, differentiation, and apoptosis. The AP-2 family belongs to key regulators of ectodermal development and skin differentiation^56^. This analysis further strengthens the association of *DNAH10* with keratinocytes’ cell cycle control. Elucidating the factors guiding *DNAH10* gene expression, as well as specific control of DNAH10 at the post-translational level can be expected to yield unprecedented insights into the physiological and pathological roles of long-term memory of epidermal-mesenchymal interactions. This will lead to the discovery of early diagnostic markers or drug targets for inflammation or altered cell proliferation as observed in precancerous lesions.

Epidermal stem cells can remember earlier inflammation by maintaining changes in their chromosomes and thus recurrent tissue damage triggers accelerated wound healing and hastened barrier restoration^25^. This altered genetic memory is considered to be beneficial, but may predispose to skin cancers or lead to autoimmune disorders of skin, like psoriasis and atopic dermatitis^25^. It is intriguing to speculate that such epidermal stem cell’s memory could explain the differences seen in thin superficial split-thickness skin grafts, even one year after the operation on dermal compartments, offering different early cues. The molecular changes associated with long term memory of epidermal cells may present as targets for therapeutic interventions to correct awry memories. Future therapeutic approaches may involve intervention in the early phase of wound healing, or in pathological skin conditions like psoriasis, by chemical or physical means to interfere with either target proteins like DNAH10, axonemal signaling, or with signaling cascades.

### Limitations

The low number of patients is compensated by the power of the study design: patients served as their own controls and only autologous tissue was used as indicator. The utilized clinical study protocol serves as a relevant human model for future studies evaluating the clinical effects of various materials on full thickness wound healing, and provides relevant results while minimizing the required number of participants. Expression of DNAH10 in the epidermis was validated using immunohistochemistry in unrelated samples from control patients, as well as non-lesional samples from psoriasis patients’ epidermis. Meticulous care was taken to perform each graft harvest similarly and by applying the same amount of pressure to the dermatome to further ascertain equal harvesting depths in addition to the same dermatome blade depth setting.

## Conclusions

Our results provide a new perspective on epithelial-mesenchymal interactions and demonstrate that long-term persistent epidermal DNAH10 expression associates with transient regenerative and inflammatory dermal signaling. We also show that DNAH10 expression is increased in inflamed psoriatic skin. These findings necessitate further investigations into the acute and delayed roles of dyneins, especially DNAH10, and the axoneme in inflammatory epithelial-mesenchymal interactions.

## Materials and Methods

A detailed description of materials and methods is available as Supplemental content.

### Protocol, assignment, participant flow and follow-up

Briefly, in ten adult patients (age range 19–58 years), one female and nine males, with large (total burn surface area range 22–45%) deep third degree burns (Supplementary Table S8), a wound area after excision onto fascia was randomized to receive three different dermal templates: 1) no dermal supplement as a control, 2) a permanent acellular dermal matrix (ADM) substitution, or 3) a non-permanent cellulose dressing for induction of granulation tissue (IGT) (Fig. 1). All treatment sites were covered with an autologous, mainly epidermal, indicator transplant.

After a follow-up period of one year, biopsies of each site of four patients were collected, epidermal and dermal compartments were separated using laser-capture microdissection, and analyzed using non-targeted, label-free proteomics. Results were validated using immunohistochemistry and primary keratinocyte cultures.

The study was conducted according to Declaration of Helsinki principles and was approved by Research Ethics Committee of the Helsinki University Hospital (DNro 101/E6/2000). Written informed consent was obtained for all participants. This clinical trial has been registered 16/01/2019 in ISRCTN Register with ISRCTN14499986.

## Supplementary information

Supplementary data and legends

## Data Availability

The mass spectrometry proteomics data are available via ProteomeXchange with identifier PXD010852.
