# Supplementary material for: Discovery of increased epidermal DNAH10 expression after regeneration of dermis in a randomized with-in person trial — reflections on psoriatic inflammation
Source: Sci Rep. 2019 Dec 13;9:19136. doi: 10.1038/s41598-019-53874-z (PMC6910998; doi:10.1038/s41598-019-53874-z)
Supplement: Supplementary file 1 — Supplementary data and legends [file 41598_2019_53874_MOESM1_ESM.pdf]

Discovery of increased epidermal DNAH10 expression after regeneration of dermis in a randomised with-in person trial - reflections on psoriatic inflammation

Heli Lagus MD[1], Mariliis Klaas PhD[2], Susanna Juteau PhD[3], Outi Elomaa PhD[4,5], Juha Kere Professor[6], Jyrki Vuola PhD[1], Viljar Jaks PhD[2], Esko Kankuri PhD[7*]

Supplementary materials

sMethods

Burn patients - This report is the third in series evaluating the effect of dermal substitution on STSG transplantation. Previously, the clinical outcome[1] and the early stage differences at the tissue level[2] have been published. The study has been conducted according to Declaration of Helsinki principles and it has been approved by Research Ethics Committee of the Helsinki University Hospital (DNro 101/E6/2000). Informed consent was obtained for all participants. This clinical trial has been registered in ISRCTN Register with ISRCTN14499986.

Out of ten patients (age range 19-58 years), with large (total burn surface area range 22-45%) deep third degree burns samples of four patients of one year follow-up were included in proteomics analysis, whereas samples from all ten patients were used for histological validations (See Figure 1 and Table S8).

Burn wounds and the clinical experiment setup - A 10x15cm area within a fascially excised deep burn on the anterior side of the body of each patient was further divided into three similarly sized sections (10x5cm). After excision, the three sections were covered as follows: one immediately with a non-meshed STSG as the control site (C), one with a permanent artificial dermal matrix (ADM), and one with a temporary viscose cellulose sponge dressing to induce formation of granulation tissue (IGT). The order of the three treatment sections was randomized in each patient by simple randomization, which resulted following orders from cranial to caudal in the ten patients: 3 x IGT-ADM-C, 2 x ADM-C-IGT, 3 x C-ADM-IGT and 2 x ADM-IGT-C. The 8/1000 inches thick, non-meshed STSGs were

harvested with a Zimmer dermatome (Zimmer Biomet, Warsaw, IN) from unaffected healthy skin areas. Viscose cellulose sponge-like matrix Cellonex (Vivoxid Ltd., Finland) containing supportive cotton fibers was selected to the study to represent a temporary wound care product inducing granulation tissue formation. The permanent artificial dermal template, Integra (Integra Life Science Corporation, Plainsboro, NJ), is a two-layer construct composed of a matrix of bovine type I collagen and shark chondroitin-6-sulfate covered by a removable silicone protecting layer. The surrounding areas of the study sites were treated with meshed autologous STSGs. Topical antibiotics or silver were not used at the study sites or in the surrounding areas.

The timeline of the study is illustrated in Figure 6. The epidermal indicator STSG was transplanted directly onto the fascia of the control site after excision. The wound beds of the two other areas were pretreated for two weeks before transplantation of the indicator STSGs. The viscose cellulose sponge dressings were removed and replaced at three and seven days after excision, and were removed after two weeks when STSGs were transplanted on the granulation tissue that was formed. At the same session, two weeks after excision, the silicone layer on the permanent artificial dermal matrix was removed, followed by STSG transplantation onto the matrix.

Samples of burn patients - For this study, 3 mm punch biopsy samples were collected from each study area one year after the wound excision. The samples were formalin fixed and then embedded in paraffin. The samples of four patients were analyzed in mass-spectrometry for protein identification and quantification. For validation of the proteomics data, immunohistochemistry for DNAH10 and caspase-14 was performed from the same samples of those four patients. Eight patient samples were used for immunohistochemistry analysis of collagen type IV and MIB-1 (Table S8).

Tissue microdissection and lysis - Three formalin-fixed, paraffin-embedded, 10-µm-thick sections from each sample block were deparaffinized by incubation at room temperature in xylene for 10 min. The deparaffinized tissue sections were then rehydrated with a graded series of ethanol and briefly air-dried.

Skin epidermis and dermis were dissected with the PALM MicroBeam laser capture microdissection system (Zeiss, Göttingen, Germany) and collected into AdhesiveCap opaque tubes (Zeiss). The tissue was lysed in a buffer consisting of 0.3 M Tris-HCl, pH 8.0, 0.1 M DTT, 0.5% (w/v) polyethylene glycol 20 000, and 4% SDS. The samples were incubated at 99 °C for 90 min as described previously[3].

Mass-spectrometry - Samples were purified by methanol/chloroform protein precipitation. Protein reduction and alkylation was performed in 100 mM Tris (pH 8.0) containing 5% sodium deoxycholate by adding 5 mM DTT and 10 mM iodoacetamide for 20 min at room temperature. Protein digestion was performed by diluting the solution 5x with mQ H2O, prior to adding 400 ng trypsin (Pierce, ThermoFischer Scientific, Waltham, MA) per reaction and by incubating overnight at +37°C. The digestion was then stopped by adding triflouroacetic acid to a concentration of 0.5%. The peptides were purified using C18 StageTips (3M Empore, Eagan, MN). Peptides were analyzed on an Ultimate 3000 RSLCnano (Dionex, ThermoFisher Scientific) nano-LC system with a C18 cartridge column (Dionex) and an in-house packed (3 µm ReproSil-Pur C18AQ particles, Dr. Maisch HPLC GmbH, Ammerbuch, Germany) 50 cm 75 µm ID emitter-column (New Objective, Woburn, MA) using a 60 min 8-50% B gradient where buffer A was 0.1% formic acid in water and buffer B was 0.1% formic acid in 80% acetonitrile. Separated peptides were eluted at 200 nl/min (spray voltage 2.4 kV) to a Q Exactive Plus (ThermoFisher Scientific) mass-spectrometer operating with a top-5 MS/MS strategy with a 0.6 s cycle time. Dynamic exclusion was set to 30 s. Only charge states +2 to +6 were analyzed. Raw data were identified and quantified with MaxQuant 1.4.0.8 software package. Search was performed against the UniProt (www.uniprot.org) human database using the tryptic digestion rule.

Analysis of proteomics data - Raw proteomics data from each sample was first normalized to the dissected tissue area, and then each sample was normalized to its detected proteins' average intensity. These sample tissue area- and intensity-normalized values were then used in subsequent analyses using

a statistically significant cutoff of $p<0.05$ for each identified protein in any comparison between groups. Clustering of proteins and sample groups was performed using Gene Cluster 3.0 software[4], and heat map visualizations were produced using Java TreeView 1.16r4 software[5].

Immunohistochemistry - Immunohistochemistry was performed on 5μm-thick paraffin embedded sections for DNAH10, caspase 14, collagen type IV, and MIB1, to validate the results of proteomic analysis. Stainings were performed with anti-DNAH10 (HPA039065, Sigma-Aldrich, Saint Louis, MO), anti-Ki-67 antigen clone MIB-1 (M7240, DakoCytomation, Glostrup, Denmark), anti-Collagen IV (M0785, DakoCytomation), anti-CASP14 (HPA027062, Sigma-Aldrich) and counterstained with Mayer’s hematoxylin (Sigma-Aldrich). Briefly, after deparaffinization, the samples underwent four cycles of heat-induced antigen retrieval in a microwave oven. After cooling and washes, the samples were blocked for endogenous peroxidase with H2O2 in methanol for 30 min. Non-specific binding was blocked using 10% normal goat serum in TBS buffer at RT for 2h. The samples were then incubated in primary antibody solution overnight at +4°C. The next day, after washes with TBS+0.025% Triton, dilutions of secondary antibody in 0.5% BSA-TBS were added and the slides were incubated for 30 min at RT. The samples were then washed and incubated in ABC-complex followed by washes and incubation in AEC solution. After color development, the slides were washed in lukewarm tap water and counterstained with Mayer’s haematoxylin. Samples were then coated with Aquamount mounting medium.

Cell experiments - Primary keratinocytes from full thickness skin samples obtained from patients undergoing reduction mammoplasties were isolated with dispase digestion and trypsinization. All donors provided written informed consent under a protocol adherent to the Helsinki Guidelines and approved by the Ethics Committee of the Hospital District of Helsinki and Uusimaa. Primary human keratinocytes were then cultured in defined keratinocyte serum-free growth medium (10744019, ThermoFisher Scientific, Gibco) for two passages and were then passaged onto six-well plates or glass

slides coated with type I collagen for the experiments with or without mitomycin C. Keratinocytes were incubated for three hours with 5 ug/ml mitomycin C (Merck KGaA, Darmstadt, Germany), then washed with culture medium and incubated in culture medium for 24 h. The slides were briefly fixed with formalin and washed in ice cold methanol. Non-specific binding was blocked using 10% normal goat serum in 1% bovine serum in TBS buffer at RT for 2h. The samples were then washed with 1% BSA-TBS and incubated in primary antibody, anti-DNAH10 rabbit IgG (HPA039065, Sigma-Aldrich) overnight at +4°C. The next day, after wash with 1% BSA-TBS the samples were incubated in secondary antibody, Alexa Fluor 555-conjugated donkey anti-rabbit IgG (A-31572, ThermoFisher Scientific), in 0.5% BSA-TBS solution at RT for 2h. After washing, the slides were mounted with ProLong Gold Antifade Mountant with DAPI (ThermoFisher Scientific). Fluorescence signals were detected using EVOS FL Imaging System (ThermoFisher Scientific).

Western Blotting - We used Western blotting to analyze expressional differences in DNAH10 in primary human keratinocytes after treatment with mitomycin C (5ug/ml). Cultures of primary human keratinocytes that had undergone 3 passages were treated with or without mitomycin-C for three hours. After incubation, cultures were washed and fresh medium was replaced. After 24 hours, cell lysates were collected into Laemmli Sample Buffer (Bio-Rad, Hertfordshire, UK), and separated using polyacrylamide gel electrophoresis (Bio-Rad). After transfer of proteins onto Whatman nitrocellulose membranes (Sigma-Aldrich), the membranes were blocked in 5% bovine serum albumin solution (Sigma-Aldrich). For the detection of DNAH10, the membranes were incubated with anti-DNAH10 antibody (Sigma-Aldrich) or anti-pan-actin (1:500; Thermo Fisher Scientific) for loading control. After washing, blots were incubated with fluorophore-conjugated IRDye secondary antibodies (goat anti-rabbit 800CW and goat anti-mouse 680LT; 1:10,000; Li-Cor Biosciences, Lincoln, NE). Fluorescence signals were detected using the Odyssey reader (Li-Cor Biosciences).

In order to elucidate the effect of inflammatory cytokines on the expression of DNAH10, P4 primary keratinocytes were cultured as above. Triplicate cultures on 3cm dishes were divided into four groups. After 48 hours of culturing cells in the keratinocyte culture media together with keratinocyte growth factors, the keratinocytes were stimulated with TNFα, TGF-β1, and with both together (both from PeproTech PE Ltd, London, UK), as well as with culture media alone as a control group. After 24 hours of incubation, Western blot was performed as described above.

Samples of psoriatic patients and healthy controls - Split thickness skin grafts of four healthy controls and four psoriatic patients were harvested with Zimmer dermatome (Zimmer Biomet) with settings of 4-6/1000 inches. The control samples, as well as samples from psoriatic patients' lesional and non-lesional areas, were collected, processed, and analyzed as described previously[6]. From the 5'-end targeted RNA-sequencing data[6] we collected the fold-change values of differentially expressed dynein and kinesin genes from the comparison between psoriatic lesional and non-lesional skin samples and compared that with other published studies. For this study, 5µm-thick sections of the formalin-fixed paraffin embedded samples were cut and stained immunohistochemically for evaluation of DNAH10 expression using the same staining protocol as detailed above for the burn patients' samples.

Analysis - Scanning of microscopy slides was carried out by the Digital microscopy and molecular pathology unit at the Finnish Institute for Molecular Medicine (FIMM, Helsinki, Finland). Microscopy images captured using the Panoramic Viewer software (3DHISTEC Ltd, Budapest, Hungary) were further analyzed using FIJI Image J software[7]. The macros used for automated quantitative analysis of signals are freely available from the authors.

The pathway analysis and the upstream and downstream protein networks were generated through the use of Ingenuity Pathway Analysis (IPA, Qiagen Bioinformatics, Redwood City, CA[8]). We uploaded

a data set of protein identifiers and their corresponding relative expression values from the proteomics results into the IPA-application. Each identifier was mapped to its corresponding object in Ingenuity's Knowledge Base, and these network-eligible molecules were overlaid onto a global molecular network developed from information contained in Ingenuity's Knowledge Base. Networks of network-eligible molecules were then algorithmically generated based on their connectivity. The functional analysis identified the biological functions and/or diseases that were most significant to the data set.

To elucidate the transcription of DNAH10 a transcription factor and gene promoter analysis was performed with latest version of the manually curated database TRANSFAC [9]. The promoter area sequence ENSR00000058778[10] was used to predict the transcription factors of DNAH10.

Statistics - Proteins from the dataset that met the $p<0.05$ cutoff and were associated with biological functions and/or diseases in Ingenuity's Knowledge Base were considered for the analysis. Right-tailed Fisher's exact test was used to calculate a p-value determining the probability that each biological function and/or disease assigned to that data set is due to chance alone.

If not otherwise stated, statistical analyses were performed using Student's-t-test for pairwise comparisons and diagrams were produced with GraphPad Prism 7.0 software (GraphPad software, LaJolla, CA). P-values less than 0.05 were considered significant. Data are expressed as mean±SD, unless otherwise specified.

Supplemental Figures presents heat maps of all identified and differentially expressed proteins (Figure S1A and S1B), IPA pathways associated with the differentially expressed proteins (Figure S2), DNAH10-associated protein-protein interactions and pathways (Figure S3). Supplementary Tables present distribution of predominant proteins in epidermis and dermis (Table S1), differentially expressed proteins (DEPs) in epidermis and dermis of STSGs (Table S2), collected data from diseases and functions associated with the proteomics results by IPA pathway analysis (Table S3), DNAH10 transcription factors and promoter analysis (Table S4) and DNAH10 transcripts predicted by

TRANSFAC analysis (Table S5) and alternative transcripts (Table S6), collected data from transcriptomic studies on mRNA of dyneins and kinesics (Table S7) as well as patient demographics in immunohistochemistry (Table S8).

References

1 Lagus, H., Sarlomo-Rikala, M., Bohling, T. and Vuola, J. (2013) Prospective study on burns treated with integra(R), a cellulose sponge and split thickness skin graft: Comparative clinical and histological study--randomized controlled trial. Burns **39,** 1577-1587

2 Lagus, H., Kankuri, E., Nuutila, K., Juteau, S., Sarlomo-Rikala, M. and Vuola, J. (2018) Induced granulation tissue but not artificial dermis enhances early host-graft interactions in full-thickness burn wounds. World J. Surg. **42,** 981-991

3 Kawashima, Y., Kodera, Y., Singh, A., Matsumoto, M. and Matsumoto, H. (2014) Efficient extraction of proteins from formalin-fixed paraffin-embedded tissues requires higher concentration of tris(hydroxymethyl)aminomethane. Clin. Proteomics **11,** 4-0275-11-4

4 de Hoon, M.J., Imoto, S., Nolan, J. and Miyano, S. (2004) Open source clustering software. Bioinformatics **20,** 1453-1454

5 Saldanha, A.J. (2004) Java treeview--extensible visualization of microarray data. Bioinformatics **20,** 3246-3248

6 Tervaniemi, M.H., Katayama, S., Skoog, T., et al. (2016) NOD-like receptor signaling and inflammasome-related pathways are highlighted in psoriatic epidermis. Sci. Rep. **6,** 22745

7 Schindelin, J., Arganda-Carreras, I., Frise, E., et al. (2012) Fiji: An open-source platform for biological-image analysis. Nat. Methods **9,** 676-682

8 Kramer, A., Green, J., Pollard, J.,Jr and Tugendreich, S. (2014) Causal analysis approaches in ingenuity pathway analysis. Bioinformatics **30,** 523-530

9 Matys, V., Kel-Margoulis, O.V., Fricke, E., et al. (2006) TRANSFAC and its module TRANSCompel: Transcriptional gene regulation in eukaryotes. Nucleic Acids Res. **34,** D108-10

10 Zerbino, D.R., Wilder, S.P., Johnson, N., Juettemann, T. and Flicek, P.R. (2015) The ensembl regulatory build. Genome Biol. **16,** 56-015-0621-5

Figures S1A and S1B

Protein distribution according to treatment group (ADM, IGT, and control) visualized in heat maps. Distribution of proteins clustered into two main branches: epidermal and dermal branches. **B.** Protein heatmaps divided into those identified from the epidermal (left panel) and dermal (right panel) samples.

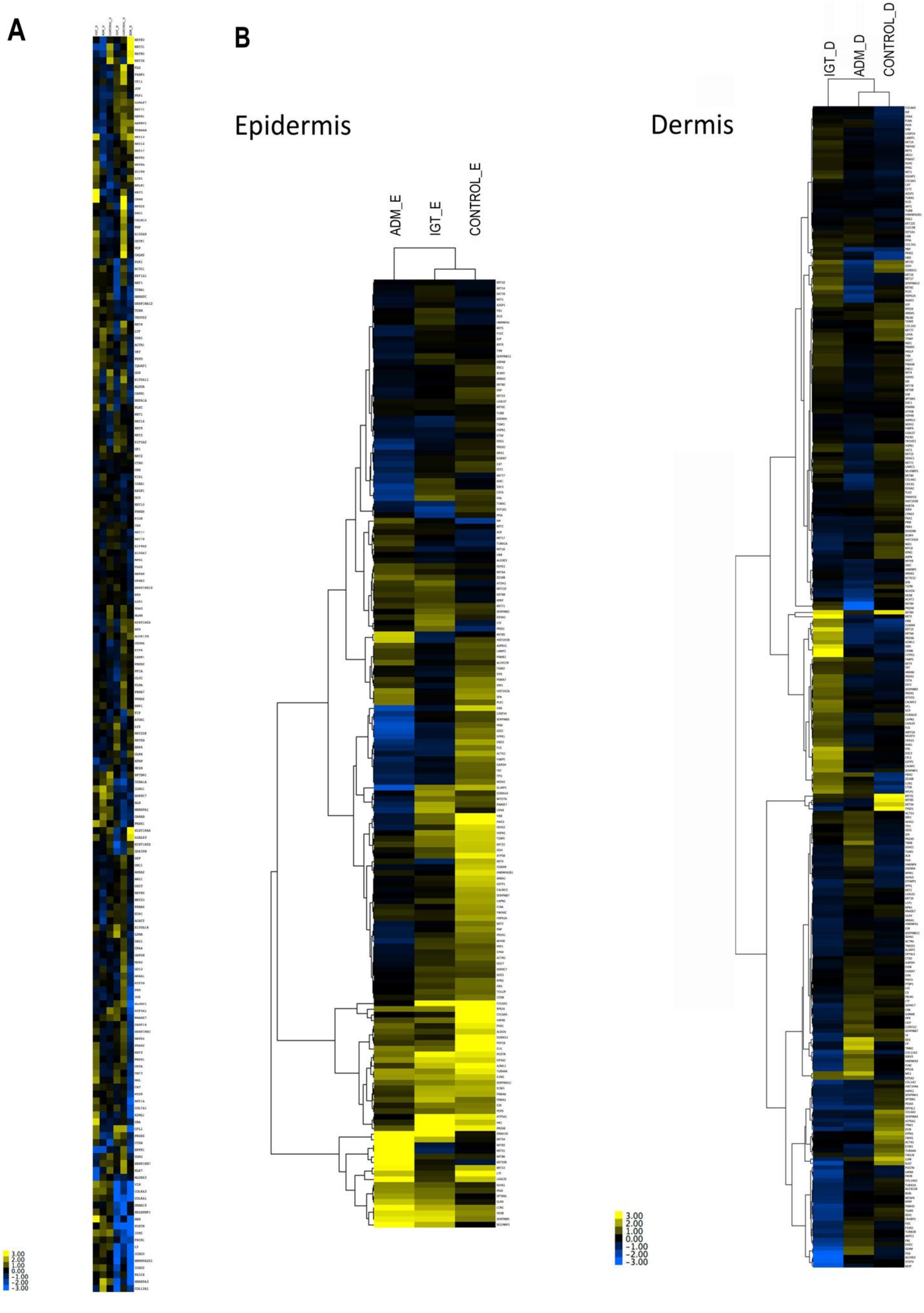
A
B
Epidermis
Dermis
ADM_E
IGT_E
CONTROL_E
IGT_D
ADM_D
CONTROL_D
3.00
2.00
1.00
0.00
−1.00
−2.00
−3.00

Figure S2

Ingenuity pathway analysis (IPA) and associations to cellular functions and diseases of differentially expressed proteins in the epidermis (upper panel) and dermis (lower panel).

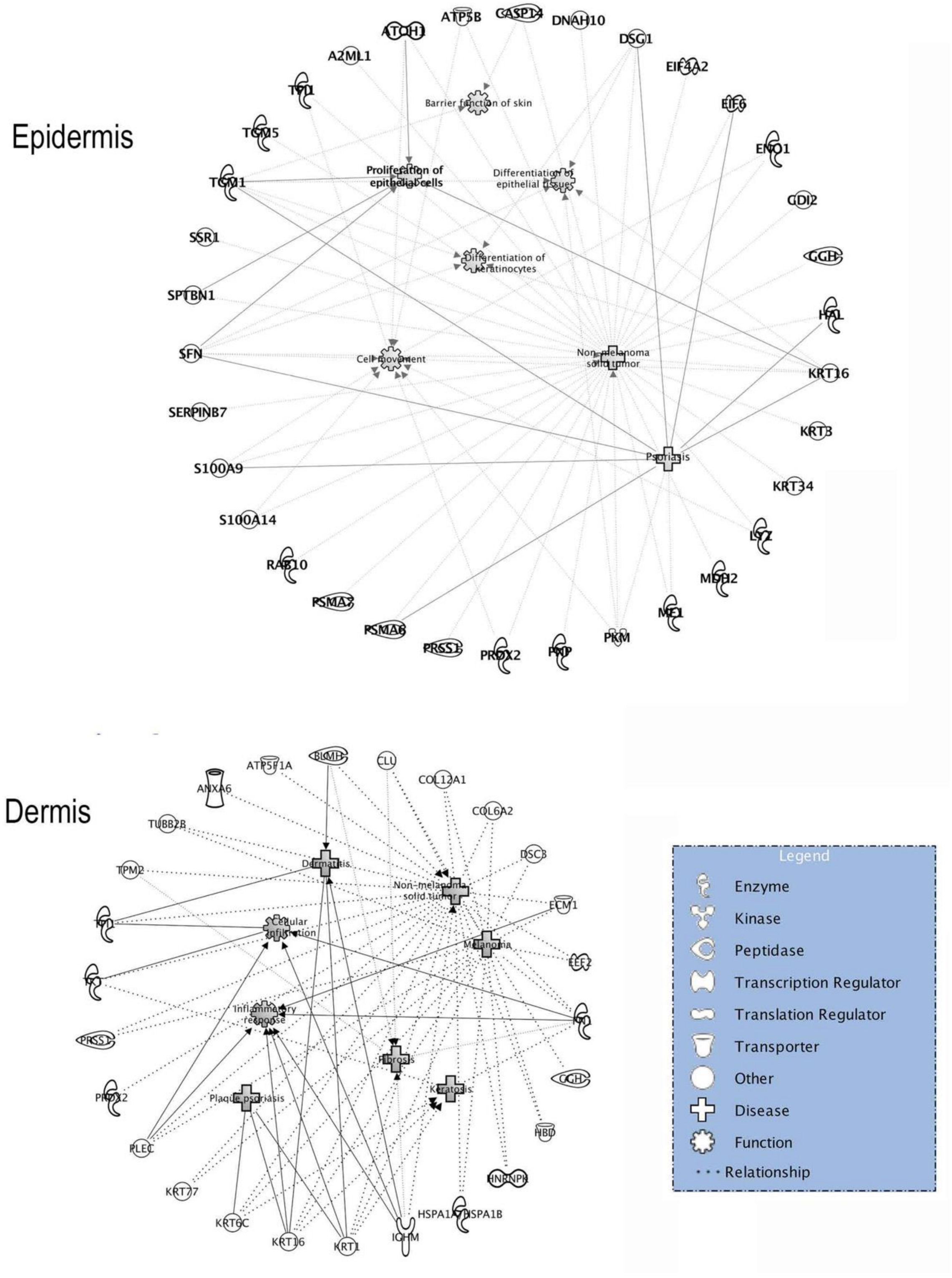

Figure S3.

Possible association of DNAH10 to various keratinocyte functions and diseases are shown in the figure.

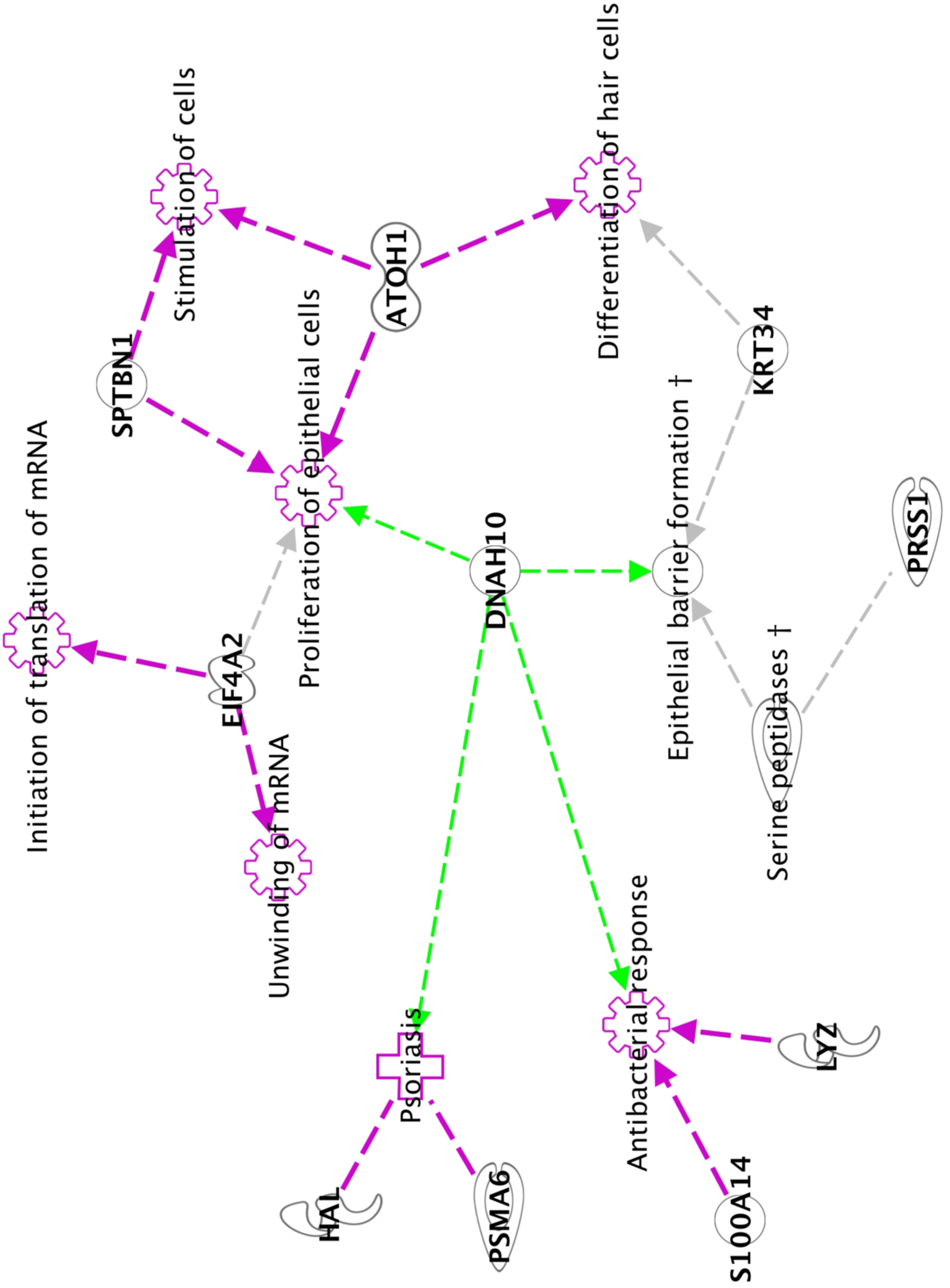

Table S1A. Identified proteins in the epidermal samples. Of the 312 proteins, 131 were found to be expressed in the epidermal samples. * $p<0.05$ between groups.

Page 1/4

Epidermal proteins identified across groups (N=131)

Protein	Induced granulation tissue IGT Mean		IGT SD	IGT N	Artificial dermal matrix ADM Mean		ADM SD	ADM N	Control Mean		Control SD	Control N	IGT vs ADM log_2 fold change	IGT vs ADM p-value		IGT vs control log_2 fold change	IGT vs control p-value		ADM vs control log_2 fold change	ADM vs control p-value	
KRT34	0.07	±	0.04	2	8.25	±	11.64	2	0.02	±	0.01	3	-6.89	0.21		1.92	0.05	*	8.81	0.14	
KRT33B	0.02	±	0.01	2	6.09	±	10.40	3	0.02	±	0.03	3	-7.94	0.25		0.04	0.49		7.98	0.18	
KRT86	0.09	±	0.07	3	17.69	±	30.51	3	0.07	±	0.04	3	-7.64	0.19		0.32	0.37		7.97	0.19	
KRT83	0.09	±	0.11	4	18.75	±	30.27	3	0.09	±	0.07	4	-7.65	0.13		0.03	0.49		7.68	0.13	
DNAH10	31.49	±	41.23	3	11.98	±	8.23	2	0.92	±	1.50	4	1.39	0.29		5.10	0.09		3.70	0.02	*
LDHA	0.12	±	0.12	2	0.01	±	0.01	2	0.04	±	0.03	4	3.29	0.16		1.74	0.10		-1.55	0.18	
RNASE7	0.06	±	0.06	2	0.01	±	0.01	2	0.03	±	0.03	3	2.92	0.17		1.07	0.23		-1.85	0.20	
SPTBN1	0.60	±	0.78	2	0.38	±	0.19	2	0.12	±	0.10	4	0.67	0.37		2.34	0.12		1.66	0.04	*
PRSS1	0.05	±	0.02	4	0.02	±	0.00	2	0.01	±	0.01	3	1.11	0.07		2.26	0.01	*	1.15	0.08	
KRT71	1.56	±	2.77	4	0.35	±	0.16	3	0.43	±	0.33	4	2.17	0.25		1.85	0.23		-0.32	0.35	
DSC3	0.11	±	0.07	4	0.02	±	0.02	2	0.06	±	0.02	4	2.16	0.10		0.98	0.10		-1.19	0.05	
HAL	0.06	±	0.01	2	0.01	±	0.02	2	0.05	±	0.04	4	2.03	0.04	*	0.24	0.38		-1.80	0.13	
MYO7A	0.02	±	0.01	2	0.00	±	0.00	2	0.02	±	0.02	3	2.00	0.19		0.01	0.50		-1.99	0.23	
S100A14	0.16	±	0.03	3	0.04	±	0.01	2	0.10	±	0.07	3	1.97	0.01	*	0.74	0.10		-1.23	0.17	
ECM1	0.09	±	0.08	3	0.03	±	0.02	2	0.08	±	0.06	3	1.79	0.18		0.20	0.43		-1.59	0.18	
PIP	0.63	±	0.43	4	1.02	±	0.58	2	0.30	±	0.36	4	-0.69	0.20		1.07	0.14		1.77	0.06	
ATOH1	0.20	±	0.07	4	0.14	±	0.09	2	0.06	±	0.03	2	0.49	0.21		1.74	0.03	*	1.24	0.18	
IGHA1	0.10	±	0.05	3	0.29	±	0.37	2	0.09	±	0.07	3	-1.58	0.20		0.14	0.43		1.72	0.19	
LYZ	0.37	±	0.14	3	0.12	±	0.02	2	0.23	±	0.29	3	1.68	0.05	*	0.71	0.24		-0.98	0.32	
POSTN	0.05	±	0.04	2	0.02	±	0.02	2	0.03	±	0.02	2	1.53	0.22		0.52	0.35		-1.01	0.21	
PSMA6	0.04	±	0.01	2	0.01	±	0.01	2	0.04	±	0.00	2	1.50	0.03	*	0.08	0.34		-1.42	0.02	*
KRT22E	1.56	±	0.93	4	1.01	±	0.50	3	0.59	±	0.07	3	0.62	0.20		1.40	0.07		0.78	0.11	
PIGR	0.04	±	0.02	3	0.07	±	0.09	2	0.03	±	0.03	2	-1.00	0.25		0.37	0.36		1.37	0.28	
ALB	2.29	±	0.65	4	5.53	±	7.68	3	2.28	±	2.48	4	-1.27	0.21		0.01	0.50		1.28	0.23	
SERPINB12	1.19	±	0.78	4	0.54	±	0.37	3	0.71	±	0.35	4	1.13	0.12		0.74	0.15		-0.40	0.28	
SDR9C7	0.03	±	0.04	3	0.02	±	0.02	2	0.01	±	0.01	3	0.96	0.33		1.09	0.26		0.13	0.46	
FIS1	4.55	±	2.79	4	5.28	±	5.11	3	2.49	±	1.74	4	-0.21	0.41		0.87	0.13		1.09	0.17	
EIF4A2	0.02	±	0.00	2	0.01	±	0.00	2	0.01	±	0.00	2	0.88	0.09		1.08	0.03	*	0.20	0.36	
KPRP	0.06	±	0.03	3	0.05	±	0.02	2	0.03	±	0.02	2	0.34	0.30		0.99	0.12		0.65	0.20	
FLG2	0.91	±	0.39	4	0.47	±	0.17	3	0.89	±	0.50	4	0.96	0.07		0.03	0.48		-0.92	0.12	
HSPA8	0.05	±	0.04	4	0.03	±	0.01	2	0.04	±	0.02	3	0.91	0.22		0.35	0.32		-0.56	0.20	
KRT5	9.79	±	3.44	4	5.59	±	3.54	3	8.21	±	3.46	4	0.81	0.09		0.25	0.27		-0.56	0.18	

Table S1A. Continued. Identified proteins in the epidermal samples. Of the 312 proteins, 131 were found to be expressed in the epidermal samples. * p<0.05 between groups.

Page 2/4

Protein	Induced granulation tissue IGT Mean		SD	N	Artificial dermal matrix ADM Mean		SD	N	Control Mean		SD	N	IGT vs ADM log_2 fold change	p-value	IGT vs control log_2 fold change	p-value		ADM vs control log_2 fold change	p-value
KRT6B	0.05	±	0.03	4	0.06	±	0.05	2	0.03	±	0.03	3	-0.22	0.41	0.58	0.27		0.80	0.29
KRT1	162.19	±	63.16	4	94.55	±	27.24	3	114.45	±	79.44	4	0.78	0.07	0.50	0.19		-0.28	0.35
KRT17	1.40	±	1.32	4	1.96	±	1.32	3	1.15	±	1.20	4	-0.49	0.30	0.28	0.39		0.77	0.22
KRT14	9.69	±	6.21	4	6.31	±	2.65	3	7.76	±	6.38	4	0.62	0.21	0.32	0.34		-0.30	0.37
KRT2	39.98	±	9.52	4	51.38	±	20.34	3	33.82	±	13.43	4	-0.36	0.18	0.24	0.24		0.60	0.11
TXN	0.24	±	0.10	4	0.17	±	0.06	2	0.23	±	0.08	4	0.51	0.20	0.08	0.43		-0.43	0.22
AZGP1	0.55	±	0.18	4	0.51	±	0.49	2	0.45	±	0.34	4	0.11	0.44	0.31	0.30		0.20	0.42
DCD	0.66	±	0.36	4	0.63	±	0.62	3	0.55	±	0.54	4	0.07	0.47	0.26	0.38		0.18	0.44
KRT10	143.28	±	53.50	4	128.54	±	45.25	3	125.00	±	79.89	4	0.16	0.36	0.20	0.36		0.04	0.47
UBB	0.04	±	0.01	4	0.05	±	0.00	2	0.05	±	0.03	4	-0.15	0.31	-0.34	0.25		-0.19	0.39
KRT6C	1.58	±	0.98	4	1.32	±	0.28	3	1.83	±	1.64	4	0.26	0.34	-0.21	0.40		-0.47	0.31
KRT6A	0.13	±	0.05	4	0.20	±	0.09	2	0.15	±	0.18	4	-0.61	0.13	-0.20	0.42		0.41	0.37
ALOXE3	0.03	±	0.02	3	0.03	±	0.01	2	0.04	±	0.04	4	-0.26	0.39	-0.63	0.27		-0.37	0.37
ANXA2	0.51	±	0.24	4	0.39	±	0.11	2	0.64	±	0.34	4	0.39	0.28	-0.33	0.28		-0.72	0.20
GSDMA	0.21	±	0.13	4	0.25	±	0.13	2	0.35	±	0.07	4	-0.23	0.38	-0.74	0.05		-0.51	0.13
ZG16B	0.05	±	0.04	3	0.07	±	0.05	2	0.09	±	0.14	3	-0.42	0.33	-0.76	0.34		-0.34	0.43
JUP	1.14	±	0.34	4	1.05	±	0.76	3	1.79	±	2.27	4	0.13	0.41	-0.64	0.30		-0.77	0.31
CTSD	0.25	±	0.17	4	0.16	±	0.07	2	0.27	±	0.26	4	0.67	0.26	-0.12	0.45		-0.79	0.30
KRT78	0.52	±	0.38	4	0.33	±	0.29	3	0.58	±	0.52	4	0.63	0.26	-0.17	0.42		-0.81	0.25
BLMH	0.28	±	0.10	4	0.27	±	0.22	3	0.51	±	0.32	4	0.01	0.50	-0.90	0.10		-0.90	0.16
LAMP1	0.07	±	0.06	4	0.11	±	0.07	2	0.13	±	0.12	4	-0.62	0.28	-0.93	0.19		-0.31	0.40
TGM1	0.10	±	0.07	4	0.13	±	0.12	2	0.20	±	0.07	4	-0.31	0.38	-0.95	0.05	*	-0.64	0.19
KRT9	35.15	±	21.25	4	25.27	±	15.69	3	49.55	±	58.51	4	0.48	0.27	-0.50	0.33		-0.97	0.26
HSPB1	0.22	±	0.18	4	0.20	±	0.13	2	0.39	±	0.35	4	0.17	0.44	-0.81	0.22		-0.98	0.26
TUBA4A	0.08	±	0.10	2	0.06	±	0.02	2	0.12	±	0.08	2	0.50	0.38	-0.57	0.35		-1.06	0.19
KRT80	0.18	±	0.11	4	0.15	±	0.09	3	0.31	±	0.21	4	0.26	0.36	-0.82	0.15		-1.08	0.14
TGM3	0.60	±	0.56	4	0.74	±	0.52	3	1.28	±	0.76	4	-0.30	0.38	-1.09	0.10		-0.79	0.17
KRT23	0.12	±	0.05	4	0.08	±	0.02	2	0.19	±	0.08	4	0.50	0.19	-0.66	0.09		-1.16	0.07
TUBA1A	0.04	±	0.03	3	0.10	±	0.09	3	0.04	±	0.04	4	-1.19	0.18	-0.06	0.48		1.13	0.16
CPA4	0.07	±	0.04	3	0.05	±	0.05	2	0.12	±	0.06	3	0.48	0.33	-0.74	0.17		-1.22	0.15
PPIA	0.07	±	0.08	4	0.09	±	0.01	2	0.16	±	0.12	4	-0.42	0.37	-1.23	0.13		-0.81	0.25
GGCT	0.02	±	0.01	3	0.01	±	0.00	2	0.02	±	0.01	3	0.77	0.20	-0.48	0.24		-1.25	0.11
TUBB	0.14	±	0.08	3	0.14	±	0.05	2	0.34	±	0.30	4	-0.01	0.49	-1.30	0.16		-1.29	0.22

Table S1A. Continued. Identified proteins in the epidermal samples. Of the 312 proteins, 131 were found to be expressed in the epidermal samples. * $p<0.05$ between groups.

	Induced granulation tissue IGT				Artificial dermal matrix ADM				Control				IGT vs ADM			IGT vs control			ADM vs control		
Protein	Mean		SD	N	Mean		SD	N	Mean		SD	N	$\log_2$ fold change	p-value		$\log_2$ fold change	p-value		$\log_2$ fold change	p-value	
DSC1	0.56	±	0.32	4	0.50	±	0.15	2	1.24	±	1.45	4	0.17	0.41		-1.13	0.20		-1.31	0.27	
KRT16	1.01	±	0.54	4	1.92	±	0.39	3	2.56	±	2.98	4	-0.93	0.03	*	-1.34	0.17		-0.41	0.37	
KRT77	0.60	±	0.33	4	0.31	±	0.33	2	0.78	±	0.86	4	0.96	0.18		-0.40	0.35		-1.36	0.25	
DSG1	2.68	±	1.03	4	1.55	±	0.82	3	3.98	±	1.20	4	0.79	0.09		-0.57	0.08		-1.36	0.02	*
EIF6	0.03	±	0.02	4	0.05	±	0.01	2	0.09	±	0.04	3	-0.44	0.28		-1.38	0.03	*	-0.94	0.11	
CDSN	0.03	±	0.02	3	0.01	±	0.00	2	0.04	±	0.03	2	0.94	0.26		-0.47	0.34		-1.42	0.20	
IGHG1	0.15	±	0.08	4	0.41	±	0.58	3	0.41	±	0.69	4	-1.41	0.21		-1.43	0.24		-0.01	0.50	
PSMA7	0.02	±	0.01	4	0.03	±	0.02	2	0.05	±	0.01	3	-0.78	0.14		-1.45	0.00	*	-0.67	0.10	
PRDX2	0.34	±	0.15	4	0.18	±	0.17	2	0.49	±	0.10	4	0.92	0.15		-0.53	0.07		-1.45	0.02	*
DSP	2.65	±	0.92	4	1.84	±	0.60	3	5.37	±	4.68	4	0.53	0.12		-1.02	0.15		-1.55	0.13	
ARG1	0.29	±	0.11	4	0.22	±	0.16	2	0.67	±	0.79	4	0.40	0.28		-1.21	0.19		-1.61	0.25	
HSPA1A	0.02	±	0.02	2	0.01	±	0.00	2	0.03	±	0.02	3	0.72	0.34		-0.92	0.22		-1.65	0.10	
LGALS7	1.01	±	0.73	4	0.79	±	0.27	3	2.52	±	3.41	4	0.35	0.32		-1.31	0.21		-1.66	0.22	
CAT	0.23	±	0.15	4	0.11	±	0.10	2	0.36	±	0.29	4	1.09	0.19		-0.66	0.23		-1.75	0.16	
TUBA1	0.38	±	0.47	4	0.26	±	0.19	2	0.87	±	0.94	4	0.58	0.37		-1.19	0.20		-1.77	0.22	
ACTN1	0.02	±	0.01	2	0.01	±	0.01	2	0.04	±	0.03	4	0.78	0.21		-1.08	0.21		-1.86	0.14	
YWHAZ	0.04	±	0.07	4	0.03	±	0.01	2	0.10	±	0.07	4	0.75	0.37		-1.13	0.17		-1.87	0.14	
SERPINB7	0.02	±	0.02	4	0.01	±	0.00	2	0.04	±	0.00	3	0.62	0.34		-1.25	0.04	*	-1.87	0.00	*
TKT	0.05	±	0.04	4	0.04	±	0.03	2	0.15	±	0.10	4	0.45	0.33		-1.46	0.06		-1.91	0.12	
KRT3	0.06	±	0.04	4	0.03	±	0.02	2	0.13	±	0.02	2	0.69	0.25		-1.23	0.03	*	-1.92	0.02	*
PKP1	0.09	±	0.07	3	0.04	±	0.02	2	0.14	±	0.15	3	1.35	0.18		-0.60	0.32		-1.95	0.21	
PRDX1	0.04	±	0.03	4	0.02	±	0.02	2	0.07	±	0.04	3	1.13	0.20		-0.83	0.15		-1.95	0.10	
GGH	0.03	±	0.01	3	0.02	±	0.01	2	0.07	±	0.03	2	0.60	0.21		-1.44	0.03	*	-2.04	0.06	
CSTA	0.11	±	0.08	3	0.03	±	0.03	2	0.14	±	0.14	3	1.79	0.15		-0.31	0.40		-2.10	0.20	
TPI1	0.08	±	0.03	4	0.06	±	0.05	3	0.26	±	0.17	4	0.37	0.30		-1.74	0.04	*	-2.11	0.05	
PKM	0.17	±	0.07	4	0.13	±	0.17	2	0.56	±	0.21	4	0.37	0.35		-1.75	0.01	*	-2.12	0.03	*
EEF2	0.05	±	0.06	4	0.02	±	0.01	2	0.07	±	0.10	4	1.56	0.25		-0.56	0.35		-2.12	0.24	
GAPDH	0.44	±	0.33	4	0.26	±	0.06	2	1.14	±	0.73	4	0.78	0.25		-1.36	0.07		-2.15	0.09	
MYH9	0.11	±	0.13	4	0.03	±	0.02	2	0.13	±	0.08	4	2.03	0.22		-0.21	0.41		-2.23	0.08	
EEF1A1	0.13	±	0.15	4	0.12	±	0.07	2	0.59	±	0.58	4	0.11	0.47		-2.13	0.09		-2.24	0.17	
EZR	0.02	±	0.01	2	0.01	±	0.00	2	0.05	±	0.04	3	1.24	0.15		-1.09	0.24		-2.33	0.15	
EIF5A2	0.03	±	0.01	2	0.02	±	0.00	2	0.08	±	0.11	2	0.96	0.12		-1.40	0.29		-2.36	0.24	
PNP	0.06	±	0.00	3	0.04	±	0.02	2	0.19	±	0.09	3	0.70	0.07		-1.72	0.03	*	-2.42	0.06	

Table S1A. Continued. Identified proteins in the epidermal samples. Of the 312 proteins, 131 were found to be expressed in the epidermal samples. * p<0.05 between groups.

	Induced granulation tissue IGT			Artificial dermal matrix ADM			Control			IGT vs ADM			IGT vs control			ADM vs control		
Protein	Mean	SD	N	Mean	SD	N	Mean	SD	N	log_2 fold change	p-value		log_2 fold change	p-value		log_2 fold change	p-value	
HIST1H2A	0.11	± 0.09	3	0.60	± 0.75	2	0.37	± 0.27	3	-2.44	0.15		-1.75	0.09		0.69	0.32	
PGK1	0.02	± 0.01	2	0.04	± 0.04	2	0.10	± 0.05	3	-1.26	0.25		-2.44	0.06		-1.18	0.14	
SERPINB3	0.30	± 0.19	4	0.16	± 0.21	2	0.89	± 0.80	4	0.89	0.23		-1.58	0.10		-2.47	0.15	
IGKC	0.12	± 0.05	3	0.04	± 0.03	2	0.20	± 0.29	4	1.77	0.05		-0.70	0.34		-2.47	0.25	
EPPK1	0.13	± 0.14	3	0.04	± 0.06	2	0.25	± 0.14	3	1.60	0.24		-0.88	0.19		-2.47	0.08	
CASP14	1.55	± 0.76	4	0.48	± 0.18	3	2.67	± 1.38	4	1.69	0.03	*	-0.78	0.10		-2.48	0.02	*
LCN1	0.04	± 0.03	3	0.21	± 0.29	2	0.04	± 0.05	2	-2.50	0.17		-0.05	0.48		2.45	0.25	
ANXA1	0.09	± 0.10	3	0.06	± 0.06	2	0.33	± 0.23	4	0.61	0.37		-1.90	0.08		-2.52	0.10	
FLG	0.12	± 0.11	4	0.09	± 0.08	2	0.54	± 0.52	4	0.37	0.39		-2.18	0.08		-2.55	0.16	
SFN	0.03	± 0.02	4	0.12	± 0.13	2	0.20	± 0.16	3	-1.80	0.10		-2.56	0.04	*	-0.76	0.30	
A2ML1	0.05	± 0.04	3	0.01	± 0.01	2	0.07	± 0.02	2	2.25	0.15		-0.40	0.33		-2.65	0.03	*
S100A9	0.08	± 0.06	4	0.05	± 0.02	2	0.35	± 0.15	2	0.66	0.27		-2.04	0.02	*	-2.71	0.06	
FABP5	0.09	± 0.05	3	0.07	± 0.08	2	0.47	± 0.63	3	0.37	0.37		-2.41	0.18		-2.77	0.23	
KRT15	0.15	± 0.22	4	0.04	± 0.04	2	0.30	± 0.40	2	1.81	0.27		-0.98	0.28		-2.79	0.23	
CALML5	0.09	± 0.04	3	0.07	± 0.01	2	0.48	± 0.55	3	0.39	0.27		-2.42	0.14		-2.81	0.19	
GSTP1	0.04	± 0.03	3	0.03	± 0.02	2	0.23	± 0.22	3	0.34	0.37		-2.48	0.11		-2.82	0.16	
S100A11	0.03	± 0.03	2	0.03	± 0.04	2	0.20	± 0.13	4	-0.31	0.43		-2.83	0.08		-2.52	0.08	
ACTG1	0.14	± 0.16	4	0.11	± 0.05	3	0.80	± 0.72	4	0.33	0.39		-2.51	0.06		-2.84	0.08	
ENO1	0.18	± 0.10	4	0.15	± 0.16	2	1.13	± 0.59	4	0.20	0.42		-2.68	0.01	*	-2.88	0.05	*
ATP5B	0.03	± 0.02	4	0.01	± 0.00	2	0.08	± 0.01	2	1.47	0.20		-1.43	0.04	*	-2.90	0.01	*
LTF	0.02	± 0.01	3	0.11	± 0.14	2	0.13	± 0.13	2	-2.72	0.15		-2.90	0.11		-0.19	0.46	
S100A7	0.58	± 0.38	4	0.24	± 0.11	2	1.87	± 2.49	4	1.26	0.15		-1.69	0.17		-2.95	0.22	
TGM5	0.01	± 0.00	2	0.01	± 0.00	2	0.06	± 0.08	2	0.97	0.03	*	-2.23	0.24		-3.20	0.21	
HIST1H2B	0.08	± 0.12	4	0.77	± 1.00	2	0.51	± 0.77	4	-3.34	0.10		-2.76	0.15		0.58	0.37	
KRT4	0.06	± 0.05	4	0.22	± 0.26	3	0.90	± 1.21	3	-1.82	0.14		-3.88	0.11		-2.06	0.20	
GDI2	0.04	± 0.02	4	0.01	± 0.00	2	0.15	± 0.10	4	1.98	0.09		-1.91	0.04	*	-3.89	0.07	
PAICS	0.01	± 0.01	2	0.00	± 0.00	2	0.04	± 0.04	4	2.26	0.18		-1.91	0.19		-4.17	0.13	
IGHG2	0.03	± 0.01	3	0.01	± 0.01	2	0.21	± 0.27	3	1.24	0.14		-3.05	0.15		-4.29	0.19	
VIM	0.48	± 0.71	4	0.08	± 0.10	2	2.68	± 4.09	3	2.56	0.25		-2.48	0.16		-5.05	0.23	
KRT85	0.15	± 0.10	4	7.45	± 12.29	3	0.35	± 0.50	3	-5.63	0.14		-1.23	0.23		4.41	0.19	
COL6A3	0.17	± 0.32	4	0.03	± 0.03	2	3.09	± 4.88	3	2.67	0.29		-4.17	0.14		-6.84	0.23	
KRT31	0.11	± 0.10	3	13.02	± 12.99	3	0.14	± 0.08	3	-6.90	0.08		-0.31	0.37		6.59	0.08	
RPS24	0.01	± 0.01	2	0.01	± 0.00	2	1.73	± 2.43	2	0.71	0.34		-7.45	0.21		-8.16	0.21	

Table S1B. Identified proteins in the dermal samples. Of the 312 proteins, 181 were found to be expressed in the dermal samples. * p<0.05 between groups.

Dermal proteins identified across samples (N=181)

	Induced granulation tissue IGT				Artificial dermal matrix ADM				Control				IGT vs ADM			IGT vs control			ADM vs control		
Protein	Mean		SD	N	Mean		SD	N	Mean		SD	N	log_2 fold change	p-value		log_2 fold change	p-value		log_2 fold change	p-value	
KRT3	1.91	±	2.44	2	0.02	±	0.01	3	0.03	±	0.03	4	6.56	0.12		5.86	0.08		-0.71	0.24	
KRT13	3.76	±	4.76	2	0.13	±	0.04	2	0.22	±	0.21	4	4.80	0.20		4.08	0.08		-0.72	0.30	
KRT6A	0.42	±	0.42	3	0.04	±	0.02	3	0.07	±	0.04	4	3.37	0.10		2.62	0.07		-0.74	0.17	
S100A9	0.48	±	0.57	3	0.07	±	0.05	3	0.05	±	0.04	4	2.80	0.14		3.27	0.09		0.48	0.28	
HBB	4.12	±	4.98	4	1.52	±	1.98	4	0.44	±	0.47	4	1.44	0.18		3.23	0.10		1.79	0.17	
HBA2	0.45	±	0.38	3	0.70	±	0.67	3	0.07	±	0.06	3	-0.62	0.31		2.60	0.08		3.22	0.09	
KRT6C	2.15	±	1.33	4	0.28	±	0.14	4	1.46	±	1.75	4	2.93	0.02	*	0.56	0.28		-2.38	0.11	
LCN1	0.05	±	0.02	3	0.06	±	0.09	3	0.01	±	0.01	2	-0.35	0.41		2.18	0.06		2.53	0.25	
CSTA	0.34	±	0.47	3	0.06	±	0.05	3	0.07	±	0.05	3	2.44	0.19		2.24	0.19		-0.21	0.42	
EIF5A2	0.03	±	0.03	2	0.04	±	0.04	3	0.01	±	0.00	2	-0.27	0.44		2.16	0.21		2.43	0.21	
KRT4	0.63	±	0.68	3	0.14	±	0.03	2	0.22	±	0.15	3	2.17	0.20		1.52	0.18		-0.65	0.27	
HAL	0.17	±	0.19	2	0.04	±	0.03	4	0.04	±	0.05	3	2.17	0.09		2.09	0.15		-0.08	0.47	
GSTP1	0.15	±	0.16	2	0.04	±	0.03	3	0.03	±	0.02	3	2.05	0.15		2.12	0.14		0.06	0.47	
IGHG1	0.84	±	0.60	4	2.44	±	3.19	4	0.58	±	0.36	4	-1.55	0.18		0.54	0.24		2.08	0.14	
CALML5	0.20	±	0.23	3	0.05	±	0.03	4	0.12	±	0.16	3	2.04	0.12		0.76	0.32		-1.28	0.21	
DSC3	0.15	±	0.03	2	0.04	±	0.01	3	0.05	±	0.06	4	2.02	0.00	*	1.48	0.06		-0.54	0.33	
SFN	0.12	±	0.16	3	0.03	±	0.02	2	0.06	±	0.00	2	2.02	0.25		1.00	0.32		-1.02	0.07	
PRDX2	0.78	±	0.57	4	0.20	±	0.08	4	0.20	±	0.07	4	1.93	0.05	*	1.98	0.04	*	0.06	0.45	
PRDX1	0.11	±	0.11	2	0.03	±	0.02	2	0.03	±	0.02	4	1.74	0.23		1.97	0.10		0.23	0.38	
LGALS3	0.05	±	0.05	2	0.01	±	0.01	2	0.02	±	0.01	2	1.96	0.22		1.19	0.28		-0.77	0.19	
ZG16B	0.21	±	0.23	3	0.11	±	0.10	4	0.06	±	0.08	4	0.95	0.23		1.88	0.13		0.93	0.22	
HSPA1A	0.04	±	0.01	2	0.01	±	0.01	3	0.03	±	0.01	3	1.87	0.02	*	0.28	0.25		-1.59	0.02	*
EEF2	0.07	±	0.03	2	0.02	±	0.01	3	0.03	±	0.02	4	1.84	0.03	*	1.18	0.08		-0.66	0.24	
KRT77	1.02	±	1.47	4	0.30	±	0.06	4	0.84	±	0.53	4	1.79	0.18		0.29	0.41		-1.50	0.05	*
S100A14	0.13	±	0.14	2	0.04	±	0.02	4	0.07	±	0.04	3	1.78	0.10		0.80	0.27		-0.99	0.09	
FABP5	0.07	±	0.08	2	0.03	±	0.03	3	0.02	±	0.01	3	0.98	0.25		1.75	0.17		0.77	0.22	
PRSS1	0.14	±	0.00	2	0.10	±	0.09	2	0.05	±	0.05	3	0.46	0.31		1.55	0.04		1.09	0.22	
YWHAZ	0.07	±	0.09	2	0.03	±	0.01	4	0.02	±	0.01	4	1.29	0.17		1.53	0.14		0.24	0.32	
FGG	0.03	±	0.02	2	0.08	±	0.05	3	0.03	±	0.01	2	-1.37	0.15		0.13	0.44		1.50	0.14	
HSPA5	0.10	±	0.13	3	0.04	±	0.01	3	0.06	±	0.05	3	1.50	0.22		0.69	0.33		-0.80	0.22	
SERPINB3	0.55	±	0.48	4	0.21	±	0.17	4	0.20	±	0.20	4	1.41	0.11		1.47	0.11		0.07	0.47	
CAPN1	0.06	±	0.05	2	0.02	±	0.02	4	0.05	±	0.03	4	1.47	0.11		0.41	0.33		-1.06	0.10	

Table S1B. Continued. Identified proteins in the dermal samples. Of the 312 proteins, 181 were found to be expressed in the dermal samples. * p<0.05 between groups.

Protein	Induced granulation tissue IGT Mean		SD	N	Artificial dermal matrix ADM Mean		SD	N	Control Mean		SD	N	IGT vs ADM log2 fold change	IGT vs ADM p-value	IGT vs control log2 fold change	IGT vs control p-value	ADM vs control log2 fold change	ADM vs control p-value
KRT9	31.37	±	23.28	4	21.31	±	11.01	4	11.54	±	5.49	4	0.56	0.23	1.44	0.07	0.88	0.08
TKT	0.14	±	0.08	2	0.10	±	0.02	3	0.05	±	0.01	4	0.52	0.21	1.39	0.04	0.87	0.00
HBD	0.10	±	0.03	3	0.09	±	0.08	3	0.04	±	0.03	3	0.15	0.43	1.39	0.04	1.23	0.20
KRT17	1.52	±	1.07	4	0.58	±	0.64	4	0.81	±	0.77	4	1.39	0.09	0.90	0.16	-0.49	0.33
HNRNPK	0.03	±	0.02	3	0.07	±	0.03	3	0.03	±	0.02	4	-1.18	0.05	0.14	0.42	1.32	0.03
GDI2	0.05	±	0.04	3	0.08	±	0.07	4	0.03	±	0.01	3	-0.73	0.27	0.58	0.28	1.31	0.17
IGHG2	0.20	±	0.10	2	0.30	±	0.25	4	0.13	±	0.07	3	-0.59	0.32	0.65	0.19	1.24	0.16
GAPDH	1.00	±	1.79	4	0.44	±	0.23	4	0.47	±	0.42	4	1.17	0.28	1.09	0.29	-0.08	0.46
KRT71	0.27	±	0.23	3	0.12	±	0.08	4	0.19	±	0.03	4	1.14	0.13	0.56	0.23	-0.58	0.11
KRT16	1.14	±	0.34	4	0.52	±	0.46	4	0.72	±	0.55	4	1.13	0.04	0.67	0.12	-0.46	0.30
PNP	0.10	±	0.02	2	0.05	±	0.06	2	0.04	±	0.03	3	0.83	0.23	1.10	0.06	0.27	0.42
ARG1	0.47	±	0.57	4	0.22	±	0.09	4	0.29	±	0.26	4	1.10	0.21	0.70	0.29	-0.40	0.32
LAMC1	0.15	±	0.11	2	0.07	±	0.05	2	0.10	±	0.05	2	1.09	0.22	0.53	0.32	-0.57	0.27
KRT1	150.96	±	60.26	4	86.09	±	29.00	4	70.92	±	37.01	4	0.81	0.05	1.09	0.03	0.28	0.27
PTBP1	0.03	±	0.02	2	0.07	±	0.07	3	0.03	±	0.00	3	-1.05	0.28	0.02	0.48	1.07	0.22
ATP5B	0.07	±	0.08	3	0.03	±	0.01	4	0.03	±	0.01	4	1.04	0.19	1.02	0.20	-0.02	0.48
ANXA6	0.06	±	0.00	2	0.05	±	0.04	2	0.03	±	0.01	3	0.18	0.41	1.04	0.02	0.85	0.19
TXN	0.30	±	0.19	4	0.15	±	0.03	4	0.29	±	0.30	3	1.02	0.08	0.05	0.48	-0.96	0.19
FLG	0.08	±	0.06	3	0.05	±	0.05	4	0.04	±	0.01	4	0.69	0.24	1.01	0.11	0.32	0.36
PSMA7	0.05	±	0.06	3	0.03	±	0.01	4	0.03	±	0.02	3	0.91	0.24	0.98	0.27	0.06	0.46
UBB	0.09	±	0.13	4	0.05	±	0.02	3	0.05	±	0.06	4	0.96	0.29	0.75	0.31	-0.21	0.43
PFN1	0.07	±	0.06	2	0.05	±	0.04	2	0.04	±	0.02	3	0.36	0.40	0.90	0.21	0.54	0.28
TPI1	0.04	±	0.01	3	0.06	±	0.02	3	0.03	±	0.01	4	-0.60	0.07	0.29	0.17	0.88	0.03
PEPD	0.04	±	0.04	2	0.03	±	0.01	2	0.02	±	0.02	2	0.72	0.31	0.88	0.29	0.16	0.43
ATOH1	0.22	±	0.15	3	0.14	±	0.14	3	0.12	±	0.12	3	0.62	0.27	0.86	0.21	0.24	0.42
CASP14	2.46	±	2.67	4	1.36	±	0.78	4	1.43	±	1.26	4	0.85	0.23	0.78	0.26	-0.07	0.46
IGHG3	0.11	±	0.13	3	0.09	±	0.00	3	0.06	±	0.04	3	0.30	0.39	0.85	0.28	0.54	0.14
KRT14	7.46	±	3.05	4	4.72	±	1.86	4	4.25	±	2.61	4	0.66	0.09	0.81	0.08	0.15	0.39
KRT78	0.52	±	0.51	4	0.29	±	0.12	4	0.44	±	0.14	4	0.81	0.21	0.24	0.39	-0.56	0.09
GSDMA	0.18	±	0.14	4	0.23	±	0.06	4	0.14	±	0.10	4	-0.36	0.26	0.40	0.31	0.76	0.08
CTSD	0.28	±	0.40	4	0.17	±	0.13	4	0.19	±	0.18	4	0.75	0.31	0.58	0.34	-0.17	0.43
PPIA	0.09	±	0.06	3	0.09	±	0.07	3	0.06	±	0.02	4	0.03	0.49	0.74	0.15	0.71	0.18
VIM	9.85	±	7.49	4	5.94	±	4.83	4	7.00	±	5.19	4	0.73	0.21	0.49	0.28	-0.24	0.39

Table S1B. Continued. Identified proteins in the dermal samples. Of the 312 proteins, 181 were found to be expressed in the dermal samples. * p<0.05 between groups.

	Induced granulation tissue IGT			Artificial dermal matrix ADM			Control			IGT vs ADM		IGT vs control		ADM vs control	
Protein	Mean	SD	N	Mean	SD	N	Mean	SD	N	log_2 fold change	p-value	log_2 fold change	p-value	log_2 fold change	p-value
MDH2	0.03	± 0.03	2	0.02	± 0.00	2	0.03	± 0.01	4	0.71	0.32	0.11	0.45	-0.61	0.10
EIF6	0.05	± 0.06	2	0.04	± 0.02	4	0.03	± 0.01	3	0.18	0.43	0.71	0.30	0.54	0.18
LAMP1	0.12	± 0.11	4	0.08	± 0.04	4	0.10	± 0.10	4	0.69	0.22	0.39	0.35	-0.31	0.37
PRELP	0.14	± 0.11	4	0.09	± 0.08	3	0.13	± 0.17	3	0.67	0.27	0.04	0.49	-0.63	0.34
CPA4	0.07	± 0.01	2	0.09	± 0.11	3	0.06	± 0.06	4	-0.40	0.40	0.26	0.40	0.66	0.30
DPYSL3	0.02	± 0.02	3	0.04	± 0.03	3	0.02	± 0.01	3	-0.55	0.31	0.10	0.45	0.65	0.26
S100A7	0.79	± 0.99	4	0.89	± 0.87	4	0.57	± 0.47	4	-0.17	0.44	0.47	0.35	0.65	0.27
NPM1	0.08	± 0.08	2	0.08	± 0.00	2	0.05	± 0.02	3	-0.07	0.48	0.57	0.31	0.64	0.11
ENO1	0.25	± 0.12	4	0.16	± 0.08	4	0.22	± 0.12	4	0.61	0.14	0.18	0.37	-0.42	0.24
EEF1A1	0.24	± 0.15	4	0.30	± 0.34	4	0.20	± 0.05	4	-0.31	0.38	0.29	0.30	0.60	0.28
ANXA2	0.54	± 0.32	4	0.36	± 0.23	4	0.42	± 0.25	4	0.60	0.19	0.37	0.28	-0.24	0.36
DSG1	1.82	± 0.66	4	2.72	± 2.36	4	1.81	± 0.53	4	-0.58	0.25	0.01	0.49	0.59	0.24
PIP	0.47	± 0.22	4	0.57	± 0.33	4	0.38	± 0.31	4	-0.28	0.32	0.29	0.34	0.57	0.22
DCD	0.68	± 0.77	4	0.47	± 0.31	4	0.63	± 0.58	4	0.53	0.32	0.11	0.46	-0.42	0.32
F13A1	0.17	± 0.22	3	0.20	± 0.06	3	0.14	± 0.12	4	-0.20	0.43	0.31	0.40	0.51	0.24
KRT5	5.76	± 1.36	4	4.79	± 2.89	4	4.16	± 1.41	4	0.27	0.28	0.47	0.08	0.20	0.36
ACTG1	0.44	± 0.34	4	0.56	± 0.33	3	0.41	± 0.38	4	-0.35	0.33	0.09	0.46	0.44	0.31
ASPRV1	0.03	± 0.03	2	0.02	± 0.01	3	0.03	± 0.01	2	0.44	0.34	0.05	0.48	-0.39	0.26
TUBA1	0.95	± 0.61	4	0.73	± 0.28	4	0.85	± 0.42	4	0.38	0.27	0.16	0.40	-0.22	0.33
IGKC	0.85	± 0.56	3	0.68	± 0.43	4	0.79	± 0.25	3	0.32	0.33	0.11	0.43	-0.21	0.36
CAT	0.39	± 0.15	4	0.44	± 0.34	4	0.35	± 0.23	4	-0.17	0.40	0.15	0.40	0.32	0.35
FLNA	0.10	± 0.09	3	0.11	± 0.07	4	0.09	± 0.06	4	-0.06	0.47	0.26	0.39	0.31	0.34
ACTN1	0.03	± 0.04	2	0.04	± 0.02	2	0.03	± 0.02	4	-0.31	0.41	0.00	0.50	0.31	0.35
COL6A3	8.42	± 10.91	4	7.16	± 7.23	4	7.40	± 6.20	4	0.23	0.43	0.19	0.44	-0.05	0.48
FSCN1	0.06	± 0.03	3	0.05	± 0.04	2	0.06	± 0.03	2	0.23	0.38	0.08	0.45	-0.15	0.44
ARF1	0.03	± 0.02	2	0.03	± 0.01	2	0.02	± 0.01	2	0.07	0.47	0.16	0.44	0.09	0.46
AZGP1	0.45	± 0.24	4	0.41	± 0.15	4	0.41	± 0.18	3	0.14	0.39	0.12	0.42	-0.02	0.48
TUBB	0.33	± 0.18	4	0.35	± 0.15	4	0.32	± 0.21	4	-0.08	0.44	0.06	0.46	0.14	0.40
ANXA1	0.20	± 0.24	4	0.18	± 0.14	4	0.18	± 0.15	4	0.09	0.47	0.10	0.46	0.01	0.49
PIGR	0.05	± 0.02	3	0.05	± 0.02	3	0.06	± 0.08	3	0.01	0.49	-0.16	0.45	-0.17	0.45
FBN1	0.10	± 0.08	3	0.09	± 0.09	2	0.11	± 0.09	3	0.10	0.47	-0.14	0.45	-0.23	0.43
COL6A1	1.23	± 1.35	4	1.47	± 2.07	4	1.51	± 1.24	4	-0.25	0.43	-0.29	0.39	-0.04	0.49
EZR	0.02	± 0.02	2	0.02	± 0.00	3	0.02	± 0.01	4	-0.31	0.33	-0.18	0.41	0.13	0.34

Table S1B. Continued. Identified proteins in the dermal samples. Of the 312 proteins, 181 were found to be expressed in the dermal samples. * p<0.05 between groups.

	Induced granulation tissue IGT				Artificial dermal matrix ADM				Control				IGT vs ADM		IGT vs control		ADM vs control	
Protein	Mean		SD	N	Mean		SD	N	Mean		SD	N	log_2 fold change	p-value	log_2 fold change	p-value	log_2 fold change	p-value
COL14A1	0.50	±	0.68	4	0.64	±	0.74	4	0.52	±	0.16	3	-0.36	0.39	-0.07	0.48	0.29	0.40
LMNA	0.06	±	0.09	3	0.08	±	0.01	3	0.06	±	0.04	4	-0.37	0.37	-0.02	0.49	0.35	0.24
FABP4	0.06	±	0.02	2	0.08	±	0.09	2	0.08	±	0.05	2	-0.40	0.40	-0.41	0.33	-0.01	0.50
DSC1	0.44	±	0.12	4	0.60	±	0.47	4	0.49	±	0.39	4	-0.44	0.27	-0.15	0.41	0.29	0.37
LGALS7	0.34	±	0.15	4	0.25	±	0.15	4	0.35	±	0.28	4	0.45	0.21	-0.03	0.48	-0.48	0.28
MYH9	0.14	±	0.10	3	0.11	±	0.09	4	0.16	±	0.13	4	0.31	0.36	-0.19	0.42	-0.51	0.28
KRT10	154.93	±	54.21	4	221.66	±	80.48	4	186.46	±	54.73	4	-0.52	0.11	-0.27	0.22	0.25	0.25
ALDOA	0.08	±	0.08	3	0.09	±	0.11	2	0.12	±	0.07	3	-0.07	0.48	-0.52	0.30	-0.45	0.36
HNRNPA2B1	0.09	±	0.08	3	0.14	±	0.21	4	0.10	±	0.08	4	-0.58	0.37	-0.07	0.47	0.51	0.37
ANXA5	0.07	±	0.03	3	0.09	±	0.07	4	0.11	±	0.06	4	-0.37	0.33	-0.61	0.18	-0.24	0.36
HNRNPC	0.02	±	0.00	2	0.02	±	0.01	2	0.02	±	0.01	3	0.37	0.30	-0.26	0.35	-0.63	0.24
TGM1	0.22	±	0.20	3	0.35	±	0.10	3	0.23	±	0.31	4	-0.63	0.20	-0.05	0.49	0.58	0.29
IGHA1	0.11	±	0.07	4	0.17	±	0.07	3	0.14	±	0.10	4	-0.63	0.16	-0.39	0.30	0.24	0.36
JUP	0.61	±	0.41	4	0.46	±	0.38	4	0.72	±	0.74	4	0.39	0.31	-0.25	0.40	-0.64	0.28
HSPA8	0.05	±	0.03	3	0.04	±	0.02	4	0.06	±	0.05	4	0.21	0.38	-0.44	0.30	-0.64	0.20
SSR1	0.02	±	0.01	2	0.04	±	0.04	2	0.03	±	0.04	3	-0.69	0.31	-0.37	0.41	0.32	0.41
CLTC	0.05	±	0.02	3	0.07	±	0.10	3	0.05	±	0.05	4	-0.69	0.33	-0.15	0.44	0.54	0.35
HIST1H2B	0.22	±	0.17	4	0.21	±	0.14	4	0.33	±	0.19	4	0.13	0.43	-0.57	0.22	-0.70	0.16
KRT2	30.57	±	21.62	4	49.73	±	10.42	4	48.47	±	30.55	4	-0.70	0.08	-0.66	0.19	0.04	0.47
PSMB2	0.07	±	0.03	3	0.04	±	0.01	4	0.07	±	0.07	3	0.61	0.08	-0.11	0.46	-0.72	0.23
TGFBI	0.12	±	0.11	3	0.12	±	0.14	4	0.20	±	0.12	3	0.01	0.50	-0.71	0.22	-0.72	0.24
HIST1H4A	0.09	±	0.10	3	0.09	±	0.07	4	0.15	±	0.11	4	0.02	0.49	-0.70	0.26	-0.72	0.20
RPS24	0.03	±	0.03	2	0.02	±	0.01	3	0.03	±	0.03	4	0.49	0.31	-0.23	0.42	-0.72	0.24
SERPINH1	0.06	±	0.08	3	0.04	±	0.03	4	0.06	±	0.01	3	0.61	0.34	-0.15	0.45	-0.76	0.14
DNAH10	11.89	±	2.61	3	11.94	±	5.76	4	20.65	±	12.00	3	-0.01	0.49	-0.80	0.14	-0.79	0.13
DSP	1.62	±	0.35	4	1.63	±	1.39	4	2.83	±	2.86	4	-0.01	0.50	-0.80	0.22	-0.80	0.24
TUBA1A	0.17	±	0.16	4	0.21	±	0.08	4	0.31	±	0.07	4	-0.32	0.32	-0.87	0.08	-0.54	0.06
SERPINB12	0.50	±	0.30	4	0.93	±	0.61	4	0.60	±	0.22	4	-0.88	0.13	-0.26	0.31	0.62	0.18
PKM	0.18	±	0.11	3	0.25	±	0.25	4	0.33	±	0.31	4	-0.52	0.32	-0.90	0.23	-0.38	0.36
HSPB1	0.10	±	0.05	3	0.07	±	0.09	4	0.13	±	0.11	4	0.55	0.31	-0.35	0.35	-0.90	0.22
KRT22E	0.77	±	0.63	4	0.72	±	0.26	4	1.36	±	1.62	4	0.10	0.45	-0.82	0.26	-0.91	0.23
LYZ	0.14	±	0.11	4	0.26	±	0.26	4	0.23	±	0.25	3	-0.91	0.21	-0.73	0.27	0.18	0.44
FIS1	3.37	±	3.99	4	6.37	±	2.18	4	4.14	±	2.02	4	-0.92	0.12	-0.30	0.37	0.62	0.09

Table S1B. Continued. Identified proteins in the dermal samples. Of the 312 proteins, 181 were found to be expressed in the dermal samples. * p<0.05 between groups.

	Induced granulation tissue IGT				Artificial dermal matrix ADM				Control				IGT vs ADM		IGT vs control		ADM vs control	
Protein	Mean		SD	N	Mean		SD	N	Mean		SD	N	log_2 fold change	p-value	log_2 fold change	p-value	log_2 fold change	p-value
HSPG2	0.21	±	0.18	3	0.22	±	0.17	3	0.40	±	0.26	2	-0.09	0.47	-0.94	0.19	-0.85	0.20
C3	0.20	±	0.12	4	0.38	±	0.45	4	0.35	±	0.21	3	-0.96	0.22	-0.81	0.14	0.15	0.45
SLURP1	0.03	±	0.01	3	0.06	±	0.04	4	0.04	±	0.02	2	-0.98	0.15	-0.17	0.39	0.81	0.23
PSMA5	0.03	±	0.03	2	0.04	±	0.04	2	0.05	±	0.05	2	-0.66	0.36	-1.02	0.29	-0.36	0.41
FBLN2	0.09	±	0.11	2	0.08	±	0.07	3	0.16	±	0.24	3	0.24	0.44	-0.79	0.37	-1.03	0.30
POSTN	0.66	±	0.62	4	1.35	±	1.76	4	0.81	±	0.89	4	-1.04	0.24	-0.29	0.40	0.75	0.30
FLG2	0.70	±	0.41	4	0.65	±	0.26	4	1.38	±	1.08	4	0.11	0.42	-0.97	0.14	-1.08	0.12
LTF	0.07	±	0.06	3	0.16	±	0.16	2	0.09	±	0.06	2	-1.09	0.22	-0.25	0.41	0.85	0.31
LGALS1	0.06	±	0.04	3	0.12	±	0.11	3	0.07	±	0.03	2	-1.11	0.20	-0.33	0.36	0.78	0.29
HNRNPA1	0.06	±	0.04	3	0.14	±	0.12	3	0.09	±	0.03	3	-1.11	0.18	-0.48	0.20	0.63	0.26
KRT23	0.13	±	0.07	3	0.08	±	0.04	4	0.19	±	0.11	3	0.64	0.17	-0.53	0.25	-1.17	0.07
BLMH	0.23	±	0.25	4	0.22	±	0.11	4	0.50	±	0.21	4	0.09	0.46	-1.11	0.08	-1.19	0.03
EFEMP1	0.09	±	0.07	4	0.21	±	0.12	4	0.10	±	0.07	3	-1.25	0.07	-0.19	0.41	1.07	0.11
LDHA	0.03	±	0.02	2	0.02	±	0.00	3	0.05	±	0.05	4	0.52	0.20	-0.76	0.31	-1.28	0.18
HIST1H2A	0.14	±	0.12	4	0.13	±	0.10	4	0.32	±	0.22	4	0.11	0.45	-1.19	0.10	-1.30	0.09
TGM3	0.61	±	0.37	4	0.67	±	0.42	4	1.50	±	1.14	4	-0.13	0.42	-1.30	0.09	-1.17	0.11
COL4A1	0.11	±	0.04	2	0.05	±	0.05	3	0.12	±	0.11	2	1.11	0.13	-0.21	0.43	-1.32	0.17
OGN	0.11	±	0.03	4	0.27	±	0.30	4	0.18	±	0.16	3	-1.33	0.16	-0.70	0.22	0.63	0.32
CDC42	0.04	±	0.02	3	0.01	±	0.00	2	0.04	±	0.01	2	1.29	0.14	-0.03	0.48	-1.33	0.06
RPN1	0.02	±	0.01	2	0.04	±	0.02	3	0.06	±	0.05	4	-0.76	0.23	-1.34	0.20	-0.59	0.28
DPYSL2	0.08	±	0.07	3	0.08	±	0.09	3	0.19	±	0.07	2	0.12	0.46	-1.24	0.09	-1.36	0.10
KRT80	0.17	±	0.10	3	0.07	±	0.04	4	0.17	±	0.12	4	1.32	0.07	-0.05	0.47	-1.37	0.07
COL1A1	0.29	±	0.28	4	0.37	±	0.46	4	0.78	±	1.02	3	-0.34	0.39	-1.43	0.19	-1.09	0.25
FBLN1	0.08	±	0.07	3	0.19	±	0.16	3	0.22	±	0.20	4	-1.18	0.18	-1.44	0.15	-0.26	0.40
ASPN	0.03	±	0.01	2	0.09	±	0.12	3	0.08	±	0.06	3	-1.50	0.28	-1.40	0.16	0.10	0.47
ALB	4.71	±	2.69	4	13.48	±	14.11	4	5.02	±	3.00	4	-1.52	0.13	-0.09	0.44	1.42	0.14
COL1A2	0.15	±	0.15	4	0.26	±	0.31	4	0.43	±	0.47	3	-0.78	0.27	-1.53	0.15	-0.74	0.29
FN1	0.17	±	0.17	4	0.48	±	0.25	3	0.28	±	0.19	3	-1.54	0.05	-0.77	0.21	0.76	0.17
MFAP4	0.06	±	0.08	2	0.07	±	0.02	2	0.20	±	0.26	2	-0.09	0.48	-1.59	0.28	-1.50	0.28
ECM1	0.03	±	0.01	2	0.02	±	0.02	4	0.06	±	0.02	2	0.59	0.24	-1.03	0.11	-1.62	0.03
C4B	0.01	±	0.00	2	0.04	±	0.02	2	0.03	±	0.01	3	-1.62	0.13	-1.30	0.10	0.32	0.34
P4HB	0.01	±	0.01	2	0.05	±	0.02	4	0.04	±	0.02	4	-1.70	0.08	-1.37	0.10	0.32	0.28
ATP5A1	0.05	±	0.03	4	0.06	±	0.07	3	0.18	±	0.08	3	-0.19	0.42	-1.71	0.02	-1.52	0.07

Table S1B. Continued. Identified proteins in the dermal samples. Of the 312 proteins, 181 were found to be expressed in the dermal samples. * p<0.05 between groups.

	Induced granulation tissue IGT				Artificial dermal matrix ADM				Control				IGT vs ADM		IGT vs control		ADM vs control	
Protein	Mean		SD	N	Mean		SD	N	Mean		SD	N	log_2 fold change	p-value	log_2 fold change	p-value	log_2 fold change	p-value
ALOX12B	0.02	±	0.01	2	0.03	±	0.01	3	0.05	±	0.04	4	-0.76	0.18	-1.78	0.12	-1.03	0.14
TUBB2B	0.01	±	0.00	2	0.03	±	0.01	2	0.02	±	0.00	2	-1.79	0.02	-1.27	0.01	0.52	0.09
IGKV3	0.02	±	0.01	3	0.06	±	0.01	2	0.03	±	0.01	2	-1.87	0.01	-0.78	0.19	1.09	0.05
TUBA4A	0.01	±	0.00	2	0.01	±	0.00	2	0.04	±	0.03	2	0.39	0.26	-1.49	0.16	-1.88	0.13
COL6A2	0.25	±	0.16	4	0.63	±	0.98	4	1.08	±	0.56	3	-1.31	0.24	-2.09	0.02	-0.78	0.26
GANAB	0.01	±	0.00	2	0.04	±	0.04	2	0.02	±	0.00	2	-2.13	0.21	-0.85	0.10	1.28	0.26
BGN	0.15	±	0.16	3	0.73	±	0.99	2	0.24	±	0.06	3	-2.27	0.18	-0.64	0.21	1.63	0.21
SERPINA3	0.02	±	0.01	2	0.02	±	0.02	2	0.08	±	0.08	2	-0.57	0.36	-2.29	0.20	-1.71	0.24
TPM2	0.02	±	0.02	2	0.01	±	0.01	2	0.05	±	0.01	2	0.78	0.30	-1.56	0.07	-2.34	0.03
S100A11	0.08	±	0.08	3	0.02	±	0.01	4	0.10	±	0.11	3	2.25	0.08	-0.25	0.43	-2.50	0.10
TYMP	0.02	±	0.01	2	0.01	±	0.00	2	0.09	±	0.12	2	0.60	0.24	-1.98	0.26	-2.57	0.24
TAGLN	0.02	±	0.01	2	0.01	±	0.01	2	0.07	±	0.09	2	0.80	0.20	-2.08	0.23	-2.87	0.21
IGHM	0.03	±	0.03	2	0.23	±	0.08	2	0.11	±	0.12	2	-3.00	0.04	-1.97	0.22	1.02	0.18
COL12A1	0.02	±	0.01	3	0.19	±	0.08	3	0.07	±	0.07	3	-3.07	0.01	-1.65	0.15	1.42	0.06
KRT85	0.33	±	0.46	2	0.04	±	0.02	2	1.42	±	1.93	2	3.06	0.23	-2.08	0.26	-5.15	0.21
KRT83	0.24	±	0.38	3	0.02	±	0.03	4	2.89	±	5.75	4	3.31	0.14	-3.58	0.24	-6.89	0.18
KRT31	0.09	±	0.14	3	0.04	±	0.04	4	19.79	±	34.14	3	1.21	0.25	-7.80	0.19	-9.01	0.14

Table S2A. Differentially expressed proteins in epidermal samples sorted according to statistical significance in comparison across groups. Table lists mass-spectroscopy-obtained expression mean values, SD, SEM for proteins in treatment groups as well as p-values for group-wise comparisons.

Page 1/2

EPIDERMAL SAMPLES

Induced granulation tissue (IGT) vs. Artificial dermal matrix (ADM); p<0.05 at least in one comparison between groups

	Induced granulation tissue (IGT)				Artificial dermal matrix (ADM)				Control				IGT vs ADM	IGT vs Control	ADM vs Control
Protein	N	Mean	SD	SEM	N	Mean	SD	SEM	N	Mean	SD	SEM	p-value	p-value	p-value
S100A14	3	**0.161**	0.032	0.019	2	**0.041**	0.007	0.005	3	**0.096**	0.065	0.038	0.0080	0.0990	0.1707
KRT16	4	**1.010**	0.543	0.271	3	**1.923**	0.388	0.224	4	**2.560**	2.976	1.488	0.0287	0.1726	0.3669
PSMA6	2	**0.038**	0.006	0.004	2	**0.014**	0.007	0.005	2	**0.036**	0.002	0.001	0.0306	0.3434	0.0213
TGM5	2	**0.013**	0.000	0.000	2	**0.007**	0.002	0.002	2	**0.061**	0.078	0.055	0.0318	0.2373	0.2137
CASP14	4	**1.551**	0.763	0.382	3	**0.479**	0.183	0.105	4	**2.665**	1.384	0.692	0.0337	0.1041	0.0226
HAL	2	**0.061**	0.012	0.008	2	**0.015**	0.017	0.012	4	**0.052**	0.036	0.018	0.0444	0.3766	0.1292
LYZ	3	**0.373**	0.140	0.081	2	**0.116**	0.015	0.011	3	**0.229**	0.292	0.169	0.0456	0.2420	0.3205

Induced granulation tissue (IGT) vs. Control; p<0.05 at least in one comparison between groups

	Induced granulation tissue (IGT)				Artificial dermal matrix (ADM)				Control				IGT vs ADM	IGT vs Control	ADM vs Control
Protein	N	Mean	SD	SEM	N	Mean	SD	SEM	N	Mean	SD	SEM	p-value	p-value	p-value
SSR1	3	**0.013**	0.007	0.004	1	**0.0217**	-	-	3	**0.043**	0.004	0.002	-	0.0016	-
PSMA7	4	**0.017**	0.008	0.004	2	**0.0290**	0.018	0.012	3	**0.046**	0.007	0.004	0.1405	0.0021	0.1046
ME1	2	**0.013**	0.009	0.007	0	**-**	-	-	4	**0.057**	0.011	0.005	-	0.0044	-
PKM	4	**0.168**	0.070	0.035	2	**0.130**	0.172	0.122	4	**0.564**	0.211	0.106	0.3502	0.0059	0.0341
ENO1	4	**0.176**	0.097	0.048	2	**0.154**	0.160	0.113	4	**1.134**	0.587	0.293	0.4165	0.0091	0.0464
PRSS1	4	**0.048**	0.018	0.009	2	**0.022**	0.004	0.003	3	**0.010**	0.008	0.005	0.0653	0.0100	0.0756
S100A9	4	**0.084**	0.061	0.031	2	**0.053**	0.016	0.011	2	**0.347**	0.152	0.107	0.2702	0.0153	0.0563
MDH2	3	**0.021**	0.013	0.007	1	**0.014**	-	-	4	**0.068**	0.025	0.012	-	0.0173	-
ATOH1	4	**0.198**	0.067	0.034	2	**0.140**	0.092	0.065	2	**0.059**	0.027	0.019	0.2117	0.0280	0.1779
KRT3	4	**0.056**	0.037	0.018	2	**0.035**	0.020	0.014	2	**0.132**	0.016	0.011	0.2486	0.0281	0.0169
EIF6	4	**0.034**	0.024	0.012	2	**0.046**	0.006	0.004	3	**0.088**	0.037	0.022	0.2770	0.0328	0.1136
PNP	3	**0.058**	0.003	0.002	2	**0.036**	0.020	0.014	3	**0.190**	0.091	0.053	0.0671	0.0328	0.0550
EIF4A2	2	**0.018**	0.003	0.002	2	**0.010**	0.004	0.003	2	**0.008**	0.001	0.001	0.0856	0.0333	0.3619
GGH	3	**0.025**	0.010	0.006	2	**0.017**	0.010	0.007	2	**0.069**	0.026	0.018	0.2131	0.0343	0.0579
TPI1	4	**0.078**	0.035	0.017	3	**0.061**	0.048	0.028	4	**0.261**	0.166	0.083	0.2969	0.0369	0.0514
ATP5B	4	**0.028**	0.025	0.012	2	**0.010**	0.001	0.000	2	**0.075**	0.015	0.010	0.1978	0.0370	0.0121
SERPINB7	4	**0.017**	0.018	0.009	2	**0.011**	0.004	0.003	3	**0.041**	0.005	0.003	0.3395	0.0397	0.0033
GDI2	4	**0.039**	0.024	0.012	2	**0.010**	0.001	0.001	4	**0.147**	0.099	0.050	0.0913	0.0401	0.0702
SFN	4	**0.034**	0.016	0.008	2	**0.118**	0.125	0.089	3	**0.200**	0.161	0.093	0.1025	0.0440	0.2965
TGM1	4	**0.102**	0.072	0.036	2	**0.127**	0.118	0.084	4	**0.198**	0.066	0.033	0.3804	0.0491	0.1871
KRT34	2	**0.070**	0.039	0.027	2	**8.246**	11.636	8.228	3	**0.018**	0.010	0.006	0.2125	0.0499	0.1361

Table S2A. Continued. Differentially expressed proteins in epidermal samples sorted according to statistical significance in comparison across groups. Table lists mass-spectroscopy-obtained expression mean values, SD, SEM for proteins in treatment groups as well as p-values for group-wise comparisons.

Page 2/2

EPIDERMAL SAMPLES

Artificial dermal matrix (ADM) vs Control; p<0.05 at least in one comparison between groups

	Induced granulation tissue (IGT)				Artificial dermal matrix (ADM)				Control				IGT vs ADM	IGT vs Control	ADM vs Control
Protein	N	Mean	SD	SEM	N	Mean	SD	SEM	N	Mean	SD	SEM	p-value	p-value	p-value
SERPINB7	4	**0.017**	0.018	0.009	2	**0.011**	0.004	0.003	3	**0.041**	0.005	0.003	0.3395	0.0397	0.0033
ATP5B	4	**0.028**	0.025	0.012	2	**0.010**	0.001	0.000	2	**0.075**	0.015	0.010	0.1978	0.0370	0.0121
DSG1	4	**2.675**	1.033	0.516	3	**1.545**	0.822	0.475	4	**3.979**	1.204	0.602	0.0908	0.0757	0.0153
KRT3	4	**0.056**	0.037	0.018	2	**0.035**	0.020	0.014	2	**0.132**	0.016	0.011	0.2486	0.0281	0.0169
PRDX2	4	**0.342**	0.154	0.077	2	**0.181**	0.169	0.120	4	**0.495**	0.095	0.048	0.1519	0.0715	0.0187
DNAH10	3	**31.486**	41.226	23.802	2	**11.978**	8.231	5.820	4	**0.921**	1.499	0.749	0.2871	0.0929	0.0208
PSMA6	2	**0.038**	0.006	0.004	2	**0.014**	0.007	0.005	2	**0.036**	0.002	0.001	0.0306	0.3434	0.0213
CASP14	4	**1.551**	0.763	0.382	3	**0.479**	0.183	0.105	4	**2.665**	1.384	0.692	0.0337	0.1041	0.0226
A2ML1	3	**0.050**	0.043	0.025	2	**0.010**	0.006	0.004	2	**0.065**	0.018	0.012	0.1549	0.3328	0.0266
PKM	4	**0.168**	0.070	0.035	2	**0.130**	0.172	0.122	4	**0.564**	0.211	0.106	0.3502	0.0059	0.0341
SPTBN1	2	**0.604**	0.783	0.554	2	**0.379**	0.188	0.133	4	**0.120**	0.096	0.048	0.3654	0.1175	0.0376
ENO1	4	**0.176**	0.097	0.048	2	**0.154**	0.160	0.113	4	**1.134**	0.587	0.293	0.4165	0.0091	0.0464
RAB10	1	**0.033**	-	-	2	**0.006**	0.001	0.001	2	**0.012**	0.003	0.002	-	-	0.0471

Table S2B. Differentially expressed proteins in dermal samples sorted according to statistical significance in comparison across groups. Table lists mass-spectroscopy-obtained expression mean values, SD, SEM for proteins in treatment groups as well as p-values for group-wise comparisons.

Page 1/2

DERMAL SAMPLES

Induced granulation tissue (IGT) vs. Artificial dermal matrix (ADM); p<0.05 at least in one comparison between groups

	Induced granulation tissue (IGT)				Artificial dermal matrix (ADM)				Control				IGT vs ADM	IGT vs Control	ADM vs Control
Protein	N	Mean	SD	SEM	N	Mean	SD	SEM	N	Mean	SD	SEM	p-value	p-value	p-value
GGH	2	**0.063**	0.007	0.005	3	**0.017**	0.002	0.001	1	**0.097**	-	-	0.0007	-	-
DSC3	2	**0.148**	0.027	0.019	3	**0.036**	0.014	0.008	4	**0.053**	0.061	0.031	0.0039	0.0576	0.3345
COL12A1	3	**0.023**	0.008	0.005	3	**0.195**	0.081	0.047	3	**0.073**	0.071	0.041	0.0109	0.1468	0.0606
IGKV3	3	**0.017**	0.013	0.008	2	**0.062**	0.010	0.007	2	**0.029**	0.012	0.009	0.0133	0.1860	0.0474
KRT6C	4	**2.145**	1.330	0.665	4	**0.281**	0.138	0.069	4	**1.459**	1.753	0.877	0.0158	0.2780	0.1144
HSPA1A	2	**0.036**	0.010	0.007	3	**0.010**	0.007	0.004	3	**0.029**	0.009	0.005	0.0203	0.2488	0.0203
TUBB2B	2	**0.009**	0.002	0.001	2	**0.031**	0.007	0.005	2	**0.022**	0.001	0.001	0.0235	0.0090	0.0944
EEF2	2	**0.066**	0.027	0.019	3	**0.019**	0.007	0.004	4	**0.029**	0.024	0.012	0.0259	0.0790	0.2446
KRT16	4	**1.136**	0.340	0.170	4	**0.521**	0.460	0.230	4	**0.716**	0.554	0.277	0.0374	0.1219	0.3035
IGHM	2	**0.029**	0.027	0.019	2	**0.231**	0.081	0.057	2	**0.114**	0.120	0.085	0.0394	0.2160	0.1845
PRDX2	4	**0.777**	0.570	0.285	4	**0.204**	0.081	0.040	4	**0.197**	0.075	0.037	0.0468	0.0449	0.4461
FN1	4	**0.166**	0.169	0.084	3	**0.482**	0.249	0.144	3	**0.284**	0.191	0.110	0.0496	0.2126	0.1678

Induced granulation tissue (IGT) vs. Control; p<0.05 at least in one comparison between groups

	Induced granulation tissue (IGT)				Artificial dermal matrix (ADM)				Control				IGT vs ADM	IGT vs Control	ADM vs Control
Protein	N	Mean	SD	SEM	N	Mean	SD	SEM	N	Mean	SD	SEM	p-value	p-value	p-value
TUBB2B	2	**0.009**	0.002	0.001	2	**0.031**	0.007	0.005	2	**0.022**	0.001	0.001	0.0235	0.0090	0.0944
COL6A2	4	**0.254**	0.157	0.079	4	**0.629**	0.984	0.492	3	**1.081**	0.560	0.323	0.2404	0.0170	0.2563
ANXA6	2	**0.062**	0.004	0.003	2	**0.054**	0.042	0.029	3	**0.030**	0.011	0.007	0.4133	0.0182	0.1895
ATP5A1	4	**0.055**	0.035	0.017	3	**0.063**	0.066	0.038	3	**0.179**	0.085	0.049	0.4223	0.0208	0.0658
KRT1	4	**150.956**	60.264	30.132	4	**86.093**	28.998	14.499	4	**70.916**	37.007	18.504	0.0502	0.0321	0.2712
TKT	2	**0.137**	0.081	0.057	3	**0.095**	0.016	0.009	4	**0.052**	0.009	0.005	0.2088	0.0385	0.0029
HBD	3	**0.095**	0.027	0.016	3	**0.086**	0.083	0.048	3	**0.036**	0.035	0.020	0.4295	0.0409	0.1971
PRSS1	2	**0.136**	0.004	0.003	2	**0.099**	0.091	0.064	3	**0.046**	0.047	0.027	0.3120	0.0417	0.2199
PRDX2	4	**0.777**	0.570	0.285	4	**0.204**	0.081	0.040	4	**0.197**	0.075	0.037	0.0468	0.0449	0.4461
CLU	3	**0.049**	0.026	0.015	0	-	-	-	3	**0.015**	0.008	0.005	-	0.0493	-

Table S2B. Continued. Differentially expressed proteins in dermal samples sorted according to statistical significance in comparison across groups. Table lists mass-spectroscopy-obtained expression mean values, SD, SEM for proteins in treatment groups as well as p-values for group-wise comparisons.

DERMAL SAMPLES

Artificial dermal matrix (ADM) vs Control; p<0.05 at least in one comparison between groups

	Induced granulation tissue (IGT)				Artificial dermal matrix (ADM)				Control				IGT vs ADM	IGT vs Control	ADM vs Control
Protein	N	Mean	SD	SEM	N	Mean	SD	SEM	N	Mean	SD	SEM	p-value	p-value	p-value
TKT	2	**0.1367**	0.0810	0.0573	3	**0.0952**	0.0156	0.0090	4	**0.0520**	0.0094	0.0047	0.2088	0.0385	0.0029
PLEC	1	**0.1182**	-	-	3	**0.0354**	0.0179	0.0104	2	**0.0879**	0.0022	0.0016	-	-	0.0149
HSPA1A	2	**0.0357**	0.0095	0.0067	3	**0.0098**	0.0074	0.0043	3	**0.0294**	0.0086	0.0050	0.0203	0.2488	0.0203
TPI1	3	**0.0393**	0.0053	0.0030	3	**0.0593**	0.0184	0.0106	4	**0.0322**	0.0104	0.0052	0.0720	0.1669	0.0268
BLMH	4	**0.2338**	0.2503	0.1251	4	**0.2204**	0.1074	0.0537	4	**0.5041**	0.2127	0.1064	0.4624	0.0754	0.0273
HNRNPK	3	**0.0327**	0.0200	0.0115	3	**0.0739**	0.0284	0.0164	4	**0.0297**	0.0192	0.0096	0.0545	0.4235	0.0278
ECM1	2	**0.0277**	0.0082	0.0058	4	**0.0184**	0.0154	0.0077	2	**0.0565**	0.0210	0.0149	0.2418	0.1065	0.0303
TPM2	2	**0.0173**	0.0152	0.0107	2	**0.0101**	0.0059	0.0042	2	**0.0512**	0.0143	0.0101	0.2965	0.0745	0.0322
KRT77	4	**1.0230**	1.4701	0.7350	4	**0.2968**	0.0644	0.0322	4	**0.8368**	0.5344	0.2672	0.1809	0.4099	0.0458
IGKV3	3	**0.0171**	0.0132	0.0076	2	**0.0622**	0.0097	0.0068	2	**0.0293**	0.0120	0.0085	0.0133	0.1860	0.0474

Table S3A. Ingenuity pathway analysis-associated diseases and functions for differentially expressed proteins across groups in epidermal samples. Skin-relevant associations highlighted in yellow.

Categories	Diseases or Functions Annotation	p-Value	Molecules	# Molecules
Cancer, Organismal Injury and Abnormalities	Non-melanoma solid tumor	3.93E-09	A2ML1,ATOH1,ATP5F1B,CASP14, DNAH10,DSG1,EIF4A2,EIF6,ENO1, GDI2,GGH,HAL,KRT16,KRT3,KRT34, LYZ,MDH2,ME1,PKM,PNP,PRDX2, PRSS1,PSMA6,PSMA7,RAB10, S100A14,S100A9,SERPINB7,SFN, SPTBN1,SSR1,TGM1,TGM5,TPI1	34
Dermatological Diseases and Conditions, Organismal Injury and Abnormalities	Chronic psoriasis	1.53E-08	HAL,KRT16,ME1,PKM,S100A9,TGM1	6
Cancer, Organismal Injury and Abnormalities	Nonhematologic malignant neoplasm	1.63E-08	A2ML1,ATOH1,ATP5F1B,CASP14, DNAH10,DSG1,EIF4A2,EIF6,ENO1, GDI2,GGH,HAL,KRT16,KRT3,KRT34, LYZ,MDH2,ME1,PKM,PNP,PRDX2, PRSS1,PSMA6,PSMA7,RAB10, S100A14,S100A9,SERPINB7,SFN, SPTBN1,SSR1,TGM1,TGM5,TPI1	34
Dermatological Diseases and Conditions, Organismal Injury and Abnormalities	Psoriasis	1.66E-08	DSG1,EIF6,HAL,KRT16,ME1,PKM, PSMA6,S100A9,SFN,TGM1	10
Cancer, Organismal Injury and Abnormalities	Carcinoma	1.82E-08	A2ML1,ATOH1,ATP5F1B,CASP14, DNAH10,DSG1,EIF4A2,EIF6,ENO1, GDI2,GGH,HAL,KRT16,KRT3,KRT34, LYZ,MDH2,ME1,PKM,PNP,PRDX2, PRSS1,PSMA7,RAB10,S100A14, S100A9,SERPINB7,SFN,SPTBN1,	33
Cancer, Organismal Injury and Abnormalities	Extracranial solid tumor	3.41E-08	A2ML1,ATOH1,ATP5F1B,CASP14, DNAH10,DSG1,EIF4A2,EIF6,ENO1, GDI2,GGH,HAL,KRT16,KRT3,KRT34, LYZ,MDH2,ME1,PKM,PNP,PRDX2, PRSS1,PSMA6,PSMA7,RAB10, S100A14,S100A9,SERPINB7,SFN, SPTBN1,SSR1,TGM1,TGM5,TPI1	34
Cancer, Organismal Injury and Abnormalities	Abdominal neoplasm	6.22E-08	A2ML1,ATOH1,ATP5F1B,CASP14, DNAH10,DSG1,EIF4A2,ENO1,GDI2, GGH,HAL,KRT16,KRT3,KRT34,LYZ, MDH2,ME1,PKM,PNP,PRDX2,PRSS1, PSMA6,PSMA7,RAB10,S100A14, S100A9,SERPINB7,SFN,SPTBN1, SSR1,TGM1,TGM5,TPI1	33
Cancer, Organismal Injury and Abnormalities	Adenocarcinoma	7.78E-07	A2ML1,ATOH1,ATP5F1B,CASP14, DNAH10,DSG1,EIF4A2,ENO1,GGH, HAL,KRT3,KRT34,LYZ,MDH2,ME1, PKM,PNP,PRDX2,PRSS1,PSMA7, RAB10,S100A14,S100A9,SERPINB7, SFN,SPTBN1,SSR1,TGM1,TGM5,TPI1	30

Table S3A. Continued. Ingenuity pathway analysis-associated diseases and functions for differentially expressed proteins across groups in epidermal samples. Skin-relevant associations highlighted in yellow.

Categories	Diseases or Functions Annotation	p-Value	Molecules	# Molecules
Embryonic Development, Hair and Skin Development and Function, Organ Development, Organismal Development, Tissue Development	Formation of skin	1.20E-06	CASP14,DSG1,KRT16,KRT34,SFN, TGM1,TGM5	7
Dermatological Diseases and Conditions, Organismal Injury and Abnormalities	Keratosis	1.37E-06	DSG1,KRT16,ME1,SERPINB7,TGM1	5
Cancer, Organismal Injury and Abnormalities	Abdominal cancer	1.76E-06	A2ML1,ATOH1,ATP5F1B,CASP14, DNAH10,DSG1,EIF4A2,ENO1,GDI2, GGH,HAL,KRT3,KRT34,LYZ,MDH2, ME1,PKM,PNP,PRDX2,PRSS1,PSMA7, RAB10,S100A14,S100A9,SERPINB7, SFN,SPTBN1,SSR1,TGM1,TGM5,TPI1	31
Dermatological Diseases and Conditions, Organismal Injury and Abnormalities	Hyperkeratosis	5.96E-06	DSG1,KRT16,SERPINB7,TGM1	4
Cellular Development, Embryonic Development, Hair and Skin Development and Function, Organ Development, Organismal Development, Tissue Development	Differentiation of skin	6.06E-06	DSG1,KRT16,SFN,TGM1,TGM5	5
Cancer, Organismal Injury and Abnormalities	Genitourinary tumor	1.71E-05	A2ML1,ATOH1,DNAH10,EIF4A2, ENO1,HAL,KRT16,KRT34,LYZ,ME1, PKM,PNP,PRDX2,PSMA6,PSMA7, S100A14,S100A9,SERPINB7,SFN, SPTBN1,SSR1,TGM1,TGM5	23
Carbohydrate Metabolism	Glycolysis of cells	4.29E-05	EIF6,ENO1,PKM,TPI1	4
Cancer, Organismal Injury and Abnormalities	Urogenital cancer	4.50E-05	A2ML1,ATOH1,DNAH10,EIF4A2, ENO1,HAL,KRT16,KRT34,LYZ,ME1, PKM,PNP,PRDX2,PSMA7,S100A14, S100A9,SERPINB7,SFN,SPTBN1,SSR1, TGM1,TGM5	22
Cellular Development, Embryonic Development, Hair and Skin Development and Function, Organ Development, Organismal Development, Tissue Development	Differentiation of keratinocytes	6.04E-05	DSG1,KRT16,SFN,TGM1	4
Cancer, Gastrointestinal Disease, Organismal Injury and Abnormalities	Digestive organ tumor	7.25E-05	A2ML1,ATOH1,ATP5F1B,CASP14, DNAH10,DSG1,EIF4A2,ENO1,GDI2, GGH,HAL,KRT16,KRT3,KRT34,LYZ, MDH2,ME1,PKM,PRDX2,PRSS1, PSMA7,RAB10,S100A14,S100A9, SERPINB7,SFN,SPTBN1,TGM5,TPI1	29

Table S3A. Continued. Ingenuity pathway analysis-associated diseases and functions for differentially expressed proteins across groups in epidermal samples. Skin-relevant associations highlighted in yellow.

Categories	Diseases or Functions Annotation	p-Value	Molecules	# Molecules
Cancer, Organismal Injury and Abnormalities, Reproductive System Disease	Genital tumor	7.65E-05	A2ML1,ATOH1,DNAH10,EIF4A2, ENO1,HAL,KRT16,KRT34,LYZ,ME1, PKM,PSMA6,PSMA7,S100A14, S100A9,SERPINB7,SFN,SPTBN1,SSR1, TGM1,TGM5	21
Cancer, Organismal Injury and Abnormalities	Cancer of secretory structure	8.76E-05	A2ML1,DNAH10,DSG1,EIF4A2,ENO1, HAL,LYZ,ME1,PKM,PNP,PRSS1, PSMA7,S100A9,SERPINB7,SFN, SPTBN1,SSR1,TGM1,TGM5	19
Embryonic Development, Hair and Skin Development and Function, Organ Development, Organismal Development, Tissue Development	Formation of epidermis	1.01E-04	CASP14,KRT16,KRT34,TGM5	4
Cancer, Gastrointestinal Disease, Organismal Injury and Abnormalities	Gastrointestinal tract cancer	1.04E-04	A2ML1,ATOH1,ATP5F1B,CASP14, DNAH10,DSG1,EIF4A2,ENO1,GGH, HAL,KRT3,KRT34,LYZ,MDH2,ME1,PKM , PRSS1,PSMA7,RAB10,S100A14, S100A9,SERPINB7,SFN,SPTBN1,TGM5, TPI1	26
Dermatological Diseases and Conditions, Developmental Disorder, Organismal Injury and Abnormalities	Congenital anomaly of skin	1.42E-04	CASP14,KRT16,TGM1,TGM5	4
Amino Acid Metabolism, Small Molecule Biochemistry	Metabolism of amino acids	1.46E-04	GGH,HAL,ME1,PKM	4
Dermatological Diseases and Conditions, Inflammatory Disease, Inflammatory Response, Organismal Injury and Abnormalities	Dermatitis	1.53E-04	DSG1,ENO1,KRT16,S100A9,SFN,TPI1	6
Cancer, Organismal Injury and Abnormalities	Head and neck neoplasia	1.78E-04	A2ML1,ATOH1,ATP5F1B,DNAH10, EIF6,ME1,PKM,PNP,PRDX2,PRSS1, S100A9,SERPINB7,SFN,SPTBN1,TGM5	15
Cancer, Organismal Injury and Abnormalities, Reproductive System Disease	Genital tract cancer	1.90E-04	A2ML1,ATOH1,DNAH10,EIF4A2, ENO1,HAL,KRT16,KRT34,LYZ,ME1, PKM,PSMA7,S100A14,S100A9, SERPINB7,SFN,SPTBN1,SSR1,TGM1, TGM5	20
Cancer, Organismal Injury and Abnormalities	Benign neoplasia	1.95E-04	ATOH1,EIF4A2,KRT16,PKM,PRDX2, PSMA6,S100A9,SPTBN1	8
Cancer, Organismal Injury and Abnormalities	Tumor in nervous system	2.28E-04	ATOH1,ATP5F1B,DNAH10,MDH2, PKM,PRSS1,S100A9,SERPINB7, SPTBN1	9

Table S3A. Continued. Ingenuity pathway analysis-associated diseases and functions for differentially expressed proteins across groups in epidermal samples. Skin-relevant associations highlighted in yellow.

Categories	Diseases or Functions Annotation	p-Value	Molecules	# Molecules
Cancer, Gastrointestinal Disease, Organismal Injury and Abnormalities	Digestive system cancer	2.51E-04	A2ML1,ATOH1,ATP5F1B,CASP14, DNAH10,DSG1,EIF4A2,ENO1,GDI2, GGH,HAL,KRT3,KRT34,LYZ,MDH2,ME1, PKM,PRSS1,PSMA7,RAB10,S100A14, S100A9,SERPINB7,SFN,SPTBN1,TGM5, TPI1	27
Cellular Development, Tissue Development	Differentiation of epithelial tissue	3.74E-04	DSG1,KRT16,PKM,SFN,TGM1	5
Cancer, Organismal Injury and Abnormalities	Head and neck cancer	3.81E-04	A2ML1,ATOH1,DNAH10,EIF6,PKM, PNP,PRSS1,S100A9,SERPINB7,SFN, SPTBN1,TGM5	12
Nucleic Acid Metabolism, Small Molecule Biochemistry	Synthesis of purine nucleotide	4.19E-04	ATP5F1B,ME1,PKM,PNP	4
Tissue Development	Growth of epithelial tissue	4.42E-04	ATOH1,KRT16,PKM,S100A9,SFN, SPTBN1,TGM1	7
Cancer, Gastrointestinal Disease, Organismal Injury and Abnormalities	Gastrointestinal carcinoma	5.11E-04	A2ML1,ATOH1,ATP5F1B,CASP14, DNAH10,DSG1,EIF4A2,GGH,HAL,KRT3, KRT34,LYZ,MDH2,ME1,PKM, PRSS1,PSMA7,RAB10,S100A14, S100A9,SERPINB7,SPTBN1,TGM5,TPI1	24
Cancer, Organismal Injury and Abnormalities	Genitourinary carcinoma	5.11E-04	A2ML1,ATOH1,DNAH10,EIF4A2,HAL, KRT16,LYZ,ME1,PKM,PNP,PRDX2, PSMA7,S100A14,SERPINB7,SFN, SPTBN1,SSR1,TGM1,TGM5	19
Dermatological Diseases and Conditions, Immunological Disease, Inflammatory Disease, Inflammatory Response, Organismal Injury and Abnormalities	Atopic dermatitis	7.12E-04	ENO1,KRT16,S100A9,TPI1	4
Cancer, Organismal Injury and Abnormalities	Development of benign tumor	7.18E-04	ATOH1,EIF4A2,PKM,PRDX2,S100A9, SPTBN1	6
Cancer, Organismal Injury and Abnormalities, Reproductive System Disease	Malignant neoplasm of male genital organ	7.38E-04	A2ML1,KRT16,LYZ,ME1,PKM,PSMA7, S100A14,S100A9,SERPINB7,SFN,SPTB N1,SSR1,TGM1,TGM5	14
Cell Death and Survival	Necrosis	8.51E-04	ATOH1,CASP14,DSG1,EIF6,ENO1,LYZ, PKM,PNP,PRDX2,PSMA6,PSMA7, S100A9,SFN,SPTBN1,TGM1	15
Cell Death and Survival	Cell viability of breast cancer cell lines	9.32E-04	PKM,PRDX2,S100A9,SFN	4
Cancer, Neurological Disease, Organismal Injury and Abnormalities	Central nervous system solid tumor	9.61E-04	ATOH1,ATP5F1B,DNAH10,PKM, PRSS1,S100A9,SERPINB7,SPTBN1	8
Cell Death and Survival	Cell death	1.03E-03	ATOH1,CASP14,DSG1,EIF6,ENO1, LYZ,PKM,PNP,PRDX2,PRSS1,PSMA6, PSMA7,S100A14,S100A9,SFN, SPTBN1,TGM1	17

Table S3A. Continued. Ingenuity pathway analysis-associated diseases and functions for differentially expressed proteins across groups in epidermal samples. Skin-relevant associations highlighted in yellow.

Categories	Diseases or Functions Annotation	p-Value	Molecules	# Molecules
Cancer, Gastrointestinal Disease, Organismal Injury and Abnormalities	Gastrointestinal adenocarcinoma	1.06E-03	A2ML1,ATOH1,ATP5F1B,CASP14, DNAH10,DSG1,GGH,HAL,KRT3,KRT34, LYZ,MDH2,ME1,PKM,PRSS1,PSMA7, RAB10,S100A14,S100A9,SERPINB7, SPTBN1,TGM5,TPI1	23
Organ Morphology	Morphology of skin	1.11E-03	ATOH1,CASP14,KRT16,TGM1	4
Immunological Disease	Hypersensitive reaction	1.24E-03	ENO1,HAL,KRT16,S100A9,TPI1	5
Cancer, Organismal Injury and Abnormalities	Head and neck carcinoma	1.33E-03	A2ML1,DNAH10,EIF6,PKM,PNP, PRSS1,S100A9,SERPINB7,SPTBN1, TGM5	10
Cancer, Neurological Disease, Organismal Injury and Abnormalities	Glioma	1.48E-03	ATP5F1B,DNAH10,PKM,PRSS1, S100A9,SERPINB7,SPTBN1	7
Cell Death and Survival	Apoptosis	1.63E-03	ATOH1,CASP14,DSG1,EIF6,ENO1, PKM,PNP,PRDX2,PRSS1,S100A14, S100A9,SFN,SPTBN1,TGM1	14
Cancer, Neurological Disease, Organismal Injury and Abnormalities	Brain tumor	1.78E-03	ATOH1,ATP5F1B,DNAH10,PKM, PRSS1,SERPINB7	6
Cancer, Organismal Injury and Abnormalities	Malignant neoplasm of upper aerodigestive tract	1.85E-03	A2ML1,ATP5F1B,LYZ,PKM,PRSS1, S100A9,SERPINB7,SFN	8
Cancer, Gastrointestinal Disease, Organismal Injury and Abnormalities	Malignant neoplasm of large intestine	2.03E-03	A2ML1,ATOH1,CASP14,DNAH10, DSG1,EIF4A2,ENO1,GGH,HAL,KRT3, KRT34,MDH2,ME1,PKM,PRSS1, PSMA7,RAB10,S100A14,S100A9, SERPINB7,SPTBN1,TGM5,TPI1	23
Cancer, Organismal Injury and Abnormalities	Melanoma	2.14E-03	A2ML1,ATP5F1B,CASP14,DNAH10, DSG1,EIF4A2,HAL,KRT16,KRT3,KRT34, MDH2,ME1,PKM,PNP,PRSS1,PSMA6, S100A9,SERPINB7,SPTBN1,TGM5	20
Protein Trafficking	Interaction of protein	2.16E-03	ENO1,ME1,PKM,TGM1	4
Cancer, Gastrointestinal Disease, Hepatic System Disease, Liver Hyperplasia/Hyperproliferation, Organismal Injury and Abnormalities	Liver tumor	2.23E-03	A2ML1,ATOH1,ATP5F1B,DNAH10, DSG1,ENO1,GDI2,HAL,KRT16,KRT34, ME1,PKM,PRDX2,PSMA7,RAB10, S100A14,S100A9,SERPINB7,SPTBN1, TGM5,TPI1	21
Protein Synthesis	Metabolism of protein	2.29E-03	CASP14,DSG1,EIF4A2,EIF6,S100A9, SERPINB7,TGM1	7
Cancer, Endocrine System Disorders, Organismal Injury and Abnormalities	Thyroid gland tumor	2.33E-03	DNAH10,PKM,PNP,PRDX2,SPTBN1, TGM5	6
Cancer, Gastrointestinal Disease, Organismal Injury and Abnormalities	Large intestine carcinoma	2.40E-03	A2ML1,ATOH1,CASP14,DNAH10, DSG1,EIF4A2,GGH,HAL,KRT3,KRT34, MDH2,ME1,PKM,PRSS1,PSMA7, RAB10,S100A14,S100A9,SERPINB7, SPTBN1,TGM5,TPI1	22
Cancer, Organismal Injury and Abnormalities	Adenoma	2.56E-03	ATOH1,EIF4A2,PKM,PRDX2,SPTBN1	5

Table S3A. Continued. Ingenuity pathway analysis-associated diseases and functions for differentially expressed proteins across groups in epidermal samples. Skin-relevant associations highlighted in yellow.

Categories	Diseases or Functions Annotation	p-Value	Molecules	# Molecules
Cancer, Organismal Injury and Abnormalities	Breast or pancreatic cancer	2.62E-03	DNAH10,DSG1,EIF4A2,EIF6,ENO1, HAL,PKM,PRDX2,PRSS1,PSMA7, S100A14,S100A9,SFN,SPTBN1,TGM1, TPI1	16
Cellular Growth and Proliferation, Tissue Development	Proliferation of epithelial cells	2.66E-03	ATOH1,KRT16,SFN,SPTBN1,TGM1	5
Nucleic Acid Metabolism, Small Molecule Biochemistry	Metabolism of nucleotide	2.67E-03	ATP5F1B,MDH2,ME1,PKM,PNP	5
Tissue Development	Development of epithelial tissue	2.81E-03	ATOH1,KRT16,PKM,S100A9,SFN	5
Cancer, Gastrointestinal Disease, Organismal Injury and Abnormalities	Upper gastrointestinal tract cancer	2.88E-03	ATP5F1B,DNAH10,LYZ,PKM,PRSS1, PSMA7,S100A9,SERPINB7,SFN, SPTBN1	10
Cell Death and Survival	Cell viability of tumor cell lines	3.32E-03	ENO1,LYZ,PKM,PRDX2,PSMA6, S100A9,SFN	7
Cancer, Organismal Injury and Abnormalities	Pelvic cancer	3.61E-03	A2ML1,ATOH1,DNAH10,EIF4A2, ENO1,HAL,KRT34,LYZ,ME1,PKM, PSMA7,S100A9,SERPINB7,SFN, SPTBN1,SSR1,TGM1,TGM5	18
Inflammatory Response, Organismal Injury and Abnormalities	Inflammation of organ	3.70E-03	DSG1,ENO1,KRT16,PKM,PRDX2,PRSS1, S100A9,SFN,TPI1	9
Cancer, Organismal Injury and Abnormalities, Reproductive System Disease	Breast or ovarian cancer	4.27E-03	DSG1,EIF4A2,EIF6,ENO1,HAL,PKM, PRDX2,PSMA7,S100A14,S100A9, SPTBN1,TGM1,TPI1	13
Cancer, Organismal Injury and Abnormalities	Neck neoplasm	4.35E-03	A2ML1,DNAH10,PKM,PNP,PRDX2, SPTBN1,TGM5	7
Cancer, Endocrine System Disorders, Organismal Injury and Abnormalities	Thyroid carcinoma	4.38E-03	DNAH10,PKM,PNP,SPTBN1,TGM5	5
Cancer, Gastrointestinal Disease, Hepatic System Disease, Liver Hyperplasia/Hyperproliferation, Organismal Injury and Abnormalities	Liver carcinoma	4.44E-03	A2ML1,ATOH1,ATP5F1B,DNAH10, DSG1,GDI2,HAL,KRT34,PKM,RAB10, SERPINB7,SPTBN1,TGM5,TPI1	14
Psychological Disorders	Major affective disorder	4.48E-03	EIF6,ME1,PSMA7,SSR1	4
Cancer, Organismal Injury and Abnormalities, Reproductive System Disease	Breast cancer	4.57E-03	DSG1,EIF4A2,EIF6,ENO1,HAL,PKM, PRDX2,PSMA7,S100A14,S100A9,TPI1	11
Cancer, Gastrointestinal Disease, Organismal Injury and Abnormalities	Gastro-esophageal carcinoma	4.76E-03	ATP5F1B,DNAH10,LYZ,PKM,PRSS1, PSMA7,SERPINB7,SPTBN1	8
Cell Death and Survival	Cell survival	5.03E-03	ENO1,LYZ,PKM,PRDX2,PSMA6, PSMA7,S100A9,SFN,TGM1	9
Cellular Movement	Cell movement	5.07E-03	ATOH1,ATP5F1B,ENO1,KRT16,LYZ, PKM,PRDX2,S100A14,S100A9,SFN,TPI	11
Cancer, Organismal Injury and Abnormalities, Renal and Urological Disease	Kidney carcinoma	5.48E-03	EIF4A2,LYZ,PKM,PNP,PRDX2,SPTBN1	6

Table S3A. Continued. Ingenuity pathway analysis-associated diseases and functions for differentially expressed proteins across groups in epidermal samples. Skin-relevant associations highlighted in yellow.

Categories	Diseases or Functions Annotation	p-Value	Molecules	# Molecules
Cancer, Gastrointestinal Disease, Organismal Injury and Abnormalities	Upper gastrointestinal tract tumor	5.59E-03	ATP5F1B,DNAH10,LYZ,ME1,PKM, PRSS1,PSMA7,S100A9,SERPINB7,SFN, SPTBN1	11
Cancer, Gastrointestinal Disease, Organismal Injury and Abnormalities	Large intestine adenocarcinoma	5.97E-03	A2ML1,ATOH1,CASP14,DNAH10, DSG1,GGH,HAL,KRT3,KRT34,MDH2, ME1,PKM,PRSS1,PSMA7,RAB10, S100A14,S100A9,SERPINB7,SPTBN1, TGM5,TPI1	21
Cancer, Endocrine System Disorders, Gastrointestinal Disease, Organismal Injury and Abnormalities	Pancreatic tumor	6.49E-03	DNAH10,DSG1,EIF4A2,ENO1,HAL, MDH2,PKM,PRSS1,SFN,SPTBN1	10
Cancer, Organismal Injury and Abnormalities	Cancer of head	6.55E-03	ATOH1,PKM,S100A9,SERPINB7	4
Inflammatory Disease	Chronic inflammatory disorder	6.63E-03	ENO1,LYZ,PKM,PRDX2,PRSS1,RAB10, S100A9	7
Cancer, Organismal Injury and Abnormalities, Reproductive System Disease	Prostate cancer	6.73E-03	A2ML1,LYZ,ME1,PKM,PSMA7, S100A9,SERPINB7,SFN,SPTBN1,SSR1, TGM1,TGM5	12
Cancer, Endocrine System Disorders, Gastrointestinal Disease, Organismal Injury and Abnormalities	Pancreatic cancer	7.39E-03	DNAH10,DSG1,EIF4A2,ENO1,HAL, PKM,PRSS1,SFN,SPTBN1	9
Carbohydrate Metabolism	Metabolism of carbohydrate	7.75E-03	PKM,PRDX2,RAB10,SFN,TPI1	5
Cancer, Organismal Injury and Abnormalities	Sarcoma	7.96E-03	A2ML1,ATOH1,HAL,PKM,PNP,SPTBN1	6
Cancer, Endocrine System Disorders, Organismal Injury and Abnormalities	Endocrine gland tumor	8.96E-03	DNAH10,MDH2,PKM,PNP,PRDX2, SPTBN1,TGM5	7
Cancer, Organismal Injury and Abnormalities	Connective or soft tissue tumor	9.13E-03	A2ML1,ATOH1,HAL,PKM,PNP,PSMA6, SPTBN1	7
Connective Tissue Disorders, Immunological Disease, Inflammatory Disease, Inflammatory Response, Organismal Injury and Abnormalities, Skeletal and Muscular Disorders	Rheumatoid arthritis	1.01E-02	ENO1,LYZ,PRDX2,RAB10,S100A9	5
Cancer, Gastrointestinal Disease, Organismal Injury and Abnormalities	Gastroesophageal adenocarcinoma	1.02E-02	ATP5F1B,DNAH10,LYZ,PKM,PSMA7, SERPINB7,SPTBN1	7
Cancer, Neurological Disease, Organismal Injury and Abnormalities	Brain astrocytoma	1.02E-02	ATP5F1B,DNAH10,PRSS1,SERPINB7	4
Cancer, Organismal Injury and Abnormalities, Respiratory Disease	Lung adenocarcinoma	1.03E-02	A2ML1,EIF4A2,ENO1,PKM,SPTBN1, TPI1	6
Embryonic Development, Organismal Development	Development of body trunk	1.05E-02	ATOH1,PKM,PRDX2,RAB10,SFN, SPTBN1	6

Table S3A. Continued. Ingenuity pathway analysis-associated diseases and functions for differentially expressed proteins across groups in epidermal samples. Skin-relevant associations highlighted in yellow.

Categories	Diseases or Functions Annotation	p-Value	Molecules	# Molecules
Cancer, Organismal Injury and Abnormalities	Squamous-cell carcinoma	1.10E-02	A2ML1,DNAH10,EIF4A2,EIF6,KRT16, ME1,PKM,PRSS1,S100A9,SERPINB7, SPTBN1	11
Cancer, Gastrointestinal Disease, Hepatic System Disease, Hepatocellular Carcinoma, Liver Hyperplasia/Hyperproliferation, Organismal Injury and Abnormalities	Hepatocellular carcinoma	1.48E-02	HAL,PKM,SPTBN1,TGM5,TPI1	5

Table S3B. Ingenuity pathway analysis-associated diseases and functions for differentially expressed proteins across groups in dermal samples. Skin-relevant associations highlighted in yellow.

Categories	Diseases or Functions Annotation	p-Value	Molecules	# Molecules
Cancer, Organismal Injury and Abnormalities, Reproductive System Disease	Malignant neoplasm of male genital organ	5.05E-08	ANXA6,BLMH,CLU,COL12A1,COL6A2, DSC3,ECM1,EEF2,FN1,HNRNPK,IGHM, KRT1,KRT16,KRT77,PLEC,TKT,TPM2	17
Cancer, Organismal Injury and Abnormalities	Urogenital cancer	1.97E-07	ANXA6,BLMH,CLU,COL12A1,COL6A2, DSC3,ECM1,EEF2,FN1,HBD,HNRNPK, HSPA1A/HSPA1B,IGHM,KRT1,KRT16, KRT77,PLEC,PRDX2,TKT,TPM2,TUBB2B	21
Cancer, Organismal Injury and Abnormalities	Abdominal neoplasm	2.41E-07	ANXA6,ATP5F1A,BLMH,CLU,COL12A1, COL6A2,DSC3,ECM1,EEF2,FN1,GGH, HBD,HNRNPK,HSPA1A/HSPA1B,IGHM, KRT1,KRT16,KRT6C,KRT77,PLEC,PRDX2, PRSS1,TKT,TPI1,TPM2,TUBB2B	26
Dermatological Diseases and Conditions, Organismal Injury and Abnormalities	Keratosis	3.37E-07	FN1,KRT1,KRT16,KRT6C,TKT	5
Cancer, Organismal Injury and Abnormalities	Non-melanoma solid tumor	3.74E-07	ANXA6,ATP5F1A,BLMH,CLU,COL12A1, COL6A2,DSC3,ECM1,EEF2,FN1,GGH, HBD,HNRNPK,HSPA1A/HSPA1B,IGHM, KRT1,KRT16,KRT6C,KRT77,PLEC,PRDX2, PRSS1,TKT,TPI1,TPM2,TUBB2B	26
Cancer, Organismal Injury and Abnormalities	Nonhematologic malignant neoplasm	1.11E-06	ANXA6,ATP5F1A,BLMH,CLU,COL12A1, COL6A2,DSC3,ECM1,EEF2,FN1,GGH, HBD,HNRNPK,HSPA1A/HSPA1B,IGHM, KRT1,KRT16,KRT6C,KRT77,PLEC,PRDX2, PRSS1,TKT,TPI1,TPM2,TUBB2B	26
Cancer, Gastrointestinal Disease, Organismal Injury and Abnormalities	Digestive organ tumor	1.51E-06	ANXA6,ATP5F1A,BLMH,CLU,COL12A1, COL6A2,DSC3,ECM1,EEF2,FN1,GGH, HBD,HNRNPK,HSPA1A/HSPA1B,KRT1, KRT16,KRT6C,KRT77,PLEC,PRDX2,PRSS1, TKT, TPI1,TPM2,TUBB2B	25
Immunological Disease	Systemic autoimmune syndrome	1.68E-06	CLU,COL12A1,COL6A2,ECM1,EEF2,FN1, HSPA1A/HSPA1B,IGHM,PRDX2,TPM2	10
Cancer, Organismal Injury and Abnormalities	Extracranial solid tumor	1.95E-06	ANXA6,ATP5F1A,BLMH,CLU,COL12A1, COL6A2,DSC3,ECM1,EEF2,FN1,GGH, HBD,HNRNPK,HSPA1A/HSPA1B,IGHM, KRT1,KRT16,KRT6C,KRT77,PLEC,PRDX2, PRSS1,TKT,TPI1,TPM2,TUBB2B	26
Dermatological Diseases and Conditions, Organismal Injury and Abnormalities	Hyperkeratosis	1.96E-06	FN1,KRT1,KRT16,KRT6C	4
Immunological Disease	Hypersensitive reaction	2.00E-06	ECM1,HSPA1A/HSPA1B,IGHM,KRT1, KRT16,KRT6C,TPI1	7
Cancer, Organismal Injury and Abnormalities	Genitourinary carcinoma	2.02E-06	ANXA6,CLU,COL12A1,COL6A2,DSC3, ECM1,EEF2,FN1,HBD,HNRNPK,HSPA1A/ HSPA1B,KRT1,KRT16,KRT77,PLEC, PRDX2,TKT,TPM2,TUBB2B	19
Cancer, Organismal Injury and Abnormalities	Carcinoma	2.08E-06	ANXA6,ATP5F1A,BLMH,CLU,COL12A1, COL6A2,DSC3,ECM1,EEF2,FN1,GGH, HBD,HNRNPK,HSPA1A/HSPA1B,KRT1, KRT16,KRT6C,KRT77,PLEC,PRDX2,PRSS1, TKT,TPI1,TPM2,TUBB2B	25

Table S3B. Continued. Ingenuity pathway analysis-associated diseases and functions for differentially expressed proteins across groups in dermal samples. Skin-relevant associations highlighted in yellow.

Categories	Diseases or Functions Annotation	p-Value	Molecules	# Molecules
Cancer, Organismal Injury and Abnormalities, Reproductive System Disease	Prostatic carcinoma	2.29E-06	ANXA6,CLU,COL12A1,COL6A2,DSC3, ECM1,EEF2,FN1,HNRNPK,KRT1,KRT77, PLEC,TKT,TPM2	14
Cancer, Organismal Injury and Abnormalities	Lymphoreticular neoplasm	2.33E-06	ANXA6,ATP5F1A,CLU,COL12A1,COL6A2, DSC3,FN1,HSPA1A/HSPA1B,IGHM,KRT1, KRT16,KRT6C,PRSS1,TPM2	14
Cancer, Organismal Injury and Abnormalities, Reproductive System Disease	Prostate cancer	2.54E-06	ANXA6,CLU,COL12A1,COL6A2,DSC3, ECM1,EEF2,FN1,HNRNPK,IGHM,KRT1, KRT77,PLEC,TKT,TPM2	15
Cancer, Hematological Disease, Organismal Injury and Abnormalities	Hematologic cancer	3.13E-06	ANXA6,ATP5F1A,CLU,COL12A1,COL6A2, DSC3,FN1,HSPA1A/HSPA1B,IGHM,KRT1, KRT16,KRT6C,PRSS1,TPM2	14
Cancer, Organismal Injury and Abnormalities, Reproductive System Disease	Prostatic adenocarcinoma	8.61E-06	ANXA6,CLU,COL12A1,COL6A2,DSC3, EEF2,FN1,HNRNPK,KRT1,KRT77,PLEC, TKT,TPM2	13
Inflammatory Response, Organismal Injury and Abnormalities	Inflammation of organ	1.15E-05	BLMH,CLU,ECM1,FN1,IGHM,KRT1, KRT16,PRDX2,PRSS1,TKT,TPI1	11
Cellular Movement	Cell movement	1.33E-05	ATP5F1A,CLU,ECM1,FN1,HNRNPK, HSPA1A/HSPA1B,IGHM,KRT16,PLEC, PRDX2,TKT,TPI1,TUBB2B	13
Developmental Disorder, Organismal Injury and Abnormalities	Dystrophy	1.38E-05	CLU,COL12A1,COL6A2,FN1,PLEC	5
Cancer, Gastrointestinal Disease, Organismal Injury and Abnormalities	Large intestine adenocarcinoma	1.64E-05	ANXA6,ATP5F1A,BLMH,CLU,COL12A1, COL6A2,DSC3,ECM1,EEF2,FN1,GGH, HBD,HNRNPK,KRT1,KRT77,PLEC, PRSS1,TKT, TPI1,TPM2,TUBB2B	21
Cancer, Organismal Injury and Abnormalities	Abdominal cancer	1.74E-05	ANXA6,ATP5F1A,BLMH,CLU,COL12A1, COL6A2,DSC3,ECM1,EEF2,FN1,GGH, HBD,HNRNPK,HSPA1A/HSPA1B,IGHM, KRT1,KRT77,PLEC,PRDX2,PRSS1,TKT, TPI1,TPM2,TUBB2B	24
Cancer, Organismal Injury and Abnormalities, Reproductive System Disease	Genital tract cancer	1.75E-05	ANXA6,BLMH,CLU,COL12A1,COL6A2, DSC3,ECM1,EEF2,FN1,HNRNPK,IGHM, KRT1,KRT16,KRT77,PLEC,TKT,TPM2, TUBB2B	18
Cancer, Organismal Injury and Abnormalities, Reproductive System Disease	Genital tumor	2.69E-05	ANXA6,BLMH,CLU,COL12A1,COL6A2, DSC3,ECM1,EEF2,FN1,HNRNPK,IGHM, KRT1,KRT16,KRT77,PLEC,TKT,TPM2, TUBB2B	18
Connective Tissue Disorders, Hereditary Disorder, Organismal Injury and Abnormalities	Hereditary connective tissue disorder	3.52E-05	CLU,COL12A1,COL6A2,HNRNPK,PLEC, TPM2	6
Immunological Disease	Allergy	3.77E-05	ECM1,IGHM,KRT1,KRT16,TKT,TPI1	6
Connective Tissue Disorders, Immunological Disease, Inflammatory Disease, Inflammatory Response, Organismal Injury and Abnormalities, Skeletal and Muscular Disorders	Rheumatoid arthritis	4.95E-05	CLU,EEF2,FN1,HSPA1A/HSPA1B,IGHM, PRDX2,TPM2	7
Immunological Disease	Immediate hypersensitivity	5.48E-05	ECM1,IGHM,KRT1,KRT16,TPI1	5
Cancer, Organismal Injury and Abnormalities	Cancer of secretory structure	6.53E-05	ANXA6,CLU,COL12A1,COL6A2,DSC3, ECM1,EEF2,FN1,HNRNPK,IGHM,KRT1, KRT77,PLEC,PRSS1,TKT,TPM2	16

Table S3B. Continued. Ingenuity pathway analysis-associated diseases and functions for differentially expressed proteins across groups in dermal samples. Skin-relevant associations highlighted in yellow.

Categories	Diseases or Functions Annotation	p-Value	Molecules	# Molecules
Endocrine System Disorders, Gastrointestinal Disease, Inflammatory Disease, Inflammatory Response, Organismal Injury and Abnormalities	Inflammation of pancreas	7.32E-05	CLU,IGHM,PRDX2,PRSS1	4
Cancer, Organismal Injury and Abnormalities	Adenocarcinoma	1.02E-04	ANXA6,ATP5F1A,BLMH,CLU,COL12A1, COL6A2,DSC3,ECM1,EEF2,FN1,GGH, HBD,HNRNPK,KRT1,KRT77,PLEC, PRDX2,PRSS1,TKT,TPI1,TPM2,TUBB2B	22
Cancer, Hematological Disease, Immunological Disease, Organismal Injury and Abnormalities	Non-Hodgkin disease	1.29E-04	ANXA6,CLU,COL12A1,COL6A2,DSC3, FN1,IGHM,KRT16	8
Developmental Disorder, Hereditary Disorder, Organismal Injury and Abnormalities, Skeletal and Muscular Disorders	Dystrophy of muscle	1.34E-04	COL12A1,COL6A2,FN1,PLEC	4
Cancer, Organismal Injury and Abnormalities	Pelvic cancer	1.68E-04	ANXA6,CLU,COL12A1,COL6A2,DSC3, ECM1,EEF2,FN1,HNRNPK,HSPA1A/ HSPA1B,IGHM,KRT1,KRT77,PLEC,TKT, TPM2,TUBB2B	17
Endocrine System Disorders, Gastrointestinal Disease, Metabolic Disease, Organismal Injury and Abnormalities	Diabetes mellitus	1.73E-04	CLU,COL12A1,COL6A2,ECM1,EEF2,FN1, HSPA1A/HSPA1B,IGHM	8
Cancer, Organismal Injury and Abnormalities	Primary solid tumor	1.78E-04	CLU,COL12A1,COL6A2,FN1,PLEC	5
Inflammatory Disease	Chronic inflammatory disorder	2.15E-04	CLU,EEF2,FN1,HSPA1A/HSPA1B,IGHM, PRDX2,PRSS1,TPM2	8
Dermatological Diseases and Conditions, Immunological Disease, Inflammatory Disease, Inflammatory Response, Organismal Injury and Abnormalities	Atopic dermatitis	2.48E-04	IGHM,KRT1,KRT16,TPI1	4
Cancer, Organismal Injury and Abnormalities	Primary tumor	2.55E-04	CLU,COL12A1,COL6A2,FN1,PLEC	5
Cancer, Organismal Injury and Abnormalities, Renal and Urological Disease	Urinary tract cancer	2.60E-04	CLU,ECM1,FN1,HBD,HNRNPK,HSPA1A/ HSPA1B,PRDX2,TPM2	8
Skeletal and Muscular Disorders, Skeletal and Muscular System Development and Function	Abnormal morphology of muscle	3.06E-04	CLU,COL12A1,FN1,PLEC	4
Cellular Development, Embryonic Development, Organismal Development	Differentiation of embryonic cells	3.06E-04	COL12A1,ECM1,FN1,TPM2	4
Protein Synthesis	Translation	3.06E-04	EEF2,FN1,HNRNPK,HSPA1A/HSPA1B	4
Cancer, Organismal Injury and Abnormalities	Connective or soft tissue tumor	3.25E-04	CLU,COL12A1,COL6A2,DSC3,FN1, HSPA1A/HSPA1B,PLEC,TKT	8
Cancer, Hematological Disease, Immunological Disease, Organismal Injury and Abnormalities	Mature T-cell neoplasm	3.40E-04	CLU,COL12A1,COL6A2,FN1,IGHM	5

Table S3B. Continued. Ingenuity pathway analysis-associated diseases and functions for differentially expressed proteins across groups in dermal samples. Skin-relevant associations highlighted in yellow.

Categories	Diseases or Functions Annotation	p-Value	Molecules	# Molecules
Hereditary Disorder, Organismal Injury and Abnormalities, Skeletal and Muscular Disorders	Hereditary myopathy	3.44E-04	COL12A1,COL6A2,FN1,PLEC,TPM2	5
Dermatological Diseases and Conditions, Inflammatory Disease, Inflammatory Response, Organismal Injury and Abnormalities	Dermatitis	3.63E-04	BLMH,IGHM,KRT1,KRT16,TPI1	5
Cancer, Hematological Disease, Immunological Disease, Organismal Injury and Abnormalities	Lymphoma	3.87E-04	ANXA6,CLU,COL12A1,COL6A2,DSC3, FN1,IGHM,KRT1,KRT16	9
Organ Morphology	Morphology of skin	3.89E-04	BLMH,KRT1,KRT16,PLEC	4
Cancer, Organismal Injury and Abnormalities	Cancer of cells	4.00E-04	ANXA6,CLU,COL12A1,COL6A2,DSC3, ECM1,FN1,IGHM,KRT1,KRT16,KRT6C, PLEC,PRDX2,PRSS1	14
Protein Synthesis	Metabolism of protein	4.14E-04	CLU,DSC3,EEF2,FN1,HNRNPK,HSPA1A/ HSPA1B,IGHM	7
Cancer, Organismal Injury and Abnormalities, Reproductive System Disease	Ductal breast carcinoma	4.14E-04	ECM1,FN1,KRT1,PLEC	4
Cell Death and Survival	Cell death of neuroblastoma cell lines	4.31E-04	ATP5F1A,CLU,HSPA1A/HSPA1B,PRDX2	4
Endocrine System Disorders, Gastrointestinal Disease, Metabolic Disease, Organismal Injury and Abnormalities	Diabetic complication	4.35E-04	CLU,COL12A1,COL6A2,FN1	4
Cancer, Organismal Injury and Abnormalities, Renal and Urological Disease	Bladder cancer	4.38E-04	CLU,ECM1,FN1,HSPA1A/HSPA1B,TPM2	5
Cancer, Organismal Injury and Abnormalities	Head and neck neoplasia	4.76E-04	CLU,COL12A1,COL6A2,DSC3,ECM1,FN1, HNRNPK,KRT1,KRT6C,PLEC,PRDX2, PRSS1	12
Cellular Development, Embryonic Development, Organismal Development, Tissue Development	Differentiation of embryonic tissue	4.77E-04	COL12A1,ECM1,FN1,TPM2	4
Inflammatory Response	Inflammation of absolute anatomical region	5.24E-04	CLU,ECM1,FN1,IGHM,PRDX2,PRSS1,TKT, TPI1	8
Cancer, Organismal Injury and Abnormalities	Head and neck cancer	5.31E-04	CLU,COL12A1,DSC3,ECM1,FN1, HNRNPK,KRT1,KRT6C,PLEC,PRSS1	10
Inflammatory Response	Inflammation of body cavity	6.26E-04	CLU,FN1,IGHM,PRDX2,PRSS1,TKT,TPI1	7
Cancer, Hematological Disease, Immunological Disease, Organismal Injury and Abnormalities	T-cell non-Hodgkin disease	6.50E-04	CLU,COL12A1,COL6A2,FN1,IGHM	5
Protein Synthesis	Synthesis of protein	6.73E-04	EEF2,FN1,HNRNPK,HSPA1A/HSPA1B, IGHM	5
Cellular Movement	Migration of cells	6.81E-04	ATP5F1A,CLU,ECM1,FN1,HNRNPK, HSPA1A/HSPA1B,KRT16,PLEC,PRDX2, TUBB2B	10
Skeletal and Muscular Disorders	Abnormality of limb	7.13E-04	COL12A1,FN1,KRT16,PLEC	4
Cancer, Hematological Disease, Immunological Disease, Organismal Injury and Abnormalities	Mature B-cell neoplasm	7.94E-04	ANXA6,COL12A1,DSC3,FN1,IGHM,KRT1, KRT16	7

Table S3B. Continued. Ingenuity pathway analysis-associated diseases and functions for differentially expressed proteins across groups in dermal samples. Skin-relevant associations highlighted in yellow.

Categories	Diseases or Functions Annotation	p-Value	Molecules	# Molecules
Cancer, Organismal Injury and Abnormalities	Development of adenocarcinoma	8.21E-04	CLU,COL12A1,COL6A2,DSC3,EEF2,FN1,HBD,KRT77,PLEC,PRDX2,TPI1,TPM2,TUBB2B	13
Cancer, Organismal Injury and Abnormalities	Invasive carcinoma	8.51E-04	CLU,FN1,HSPA1A/HSPA1B,TPM2	4
Cancer, Organismal Injury and Abnormalities, Reproductive System Disease	Breast or ovarian cancer	8.79E-04	ATP5F1A,CLU,COL12A1,COL6A2,ECM1,FN1,IGHM,KRT1,PLEC,PRDX2,TPI1,TPM2	12
Cancer, Organismal Injury and Abnormalities	Melanoma	8.81E-04	CLU,COL12A1,COL6A2,DSC3,ECM1,EEF2,FN1,HBD,HNRNPK,HSPA1A/HSPA1B,KRT1,KRT16,KRT6C,KRT77,PLEC,PRSS1,TUBB2B	17
Protein Synthesis	Expression of protein	9.50E-04	FN1,HNRNPK,HSPA1A/HSPA1B,IGHM	4
Cardiovascular System Development and Function	Development of vasculature	1.02E-03	ATP5F1A,CLU,ECM1,FN1,IGHM,KRT1,TKT	7
Metabolic Disease, Neurological Disease, Organismal Injury and Abnormalities, Psychological Disorders	Alzheimer disease	1.03E-03	ATP5F1A,CLU,EEF2,HSPA1A/HSPA1B,IGHM	5
Skeletal and Muscular System Development and Function	Function of muscle	1.05E-03	ANXA6,CLU,COL12A1,PLEC	4
Cancer, Hematological Disease, Organismal Injury and Abnormalities	Malignant myeloid neoplasm	1.11E-03	ATP5F1A,COL12A1,COL6A2,HSPA1A/HSPA1B,KRT6C,PRSS1,TPM2	7
Cellular Movement	Cellular infiltration	1.14E-03	FN1,IGHM,PLEC,TKT,TPI1	5
Cell Morphology, Organ Morphology, Skeletal and Muscular System Development and Function, Tissue Morphology	Morphology of muscle cells	1.23E-03	CLU,COL12A1,FN1,PLEC	4
Cancer, Organismal Injury and Abnormalities, Reproductive System Disease	Female genital tract cancer	1.30E-03	CLU,COL12A1,COL6A2,DSC3,EEF2,FN1,IGHM,KRT1,KRT77,PLEC,TPM2,TUBB2B	12
Cancer, Hematological Disease, Organismal Injury and Abnormalities	Bone marrow cancer	1.37E-03	ATP5F1A,COL12A1,COL6A2,HSPA1A/HSPA1B,KRT6C,PRSS1,TPM2	7
Organismal Injury and Abnormalities	Fibrosis	1.49E-03	BLMH,CLU,FN1,IGHM,TPM2	5
Cancer, Organismal Injury and Abnormalities, Reproductive System Disease	Breast cancer	1.58E-03	ATP5F1A,CLU,COL12A1,ECM1,FN1,KRT1,PLEC,PRDX2,TPI1,TPM2	10
Cancer, Hematological Disease, Immunological Disease, Organismal Injury and Abnormalities	B cell cancer	1.63E-03	ANXA6,COL12A1,DSC3,FN1,IGHM,KRT16	6
Organismal Survival	Organismal death	1.70E-03	BLMH,COL12A1,COL6A2,DSC3,ECM1,FN1,HNRNPK,IGHM,KRT1,PLEC,TKT	11
Cancer, Hematological Disease, Organismal Injury and Abnormalities	Hematologic cancer of cells	1.89E-03	CLU,COL12A1,COL6A2,FN1,IGHM,KRT1	6
Cancer, Organismal Injury and Abnormalities, Reproductive System Disease	Tumorigenesis of reproductive tract	1.93E-03	CLU,COL12A1,COL6A2,DSC3,EEF2,FN1,IGHM,KRT1,KRT77,PLEC,TPM2,TUBB2B	12
Cell Death and Survival	Cell viability	1.93E-03	CLU,ECM1,EEF2,FN1,HNRNPK,HSPA1A/HSPA1B,IGHM,PRDX2	8
Inflammatory Response	Inflammatory response	1.98E-03	ECM1,FN1,IGHM,KRT1,KRT16,PLEC	6

Table S3B. Continued. Ingenuity pathway analysis-associated diseases and functions for differentially expressed proteins across groups in dermal samples. Skin-relevant associations highlighted in yellow.

Categories	Diseases or Functions Annotation	p-Value	Molecules	# Molecules
Cancer, Organismal Injury and Abnormalities, Renal and Urological Disease	Renal cancer	2.16E-03	CLU,FN1,HBD,HNRNPK,PRDX2,TPM2	6
Cell-To-Cell Signaling and Interaction	Aggregation of cells	2.31E-03	CLU,FN1,IGHM,PRDX2	4
Cancer, Organismal Injury and Abnormalities, Reproductive System Disease	Uterine cancer	2.34E-03	CLU,COL12A1,DSC3,EEF2,FN1,KRT1, KRT77,PLEC,TPM2,TUBB2B	10
Cancer, Organismal Injury and Abnormalities	Liquid tumor	2.35E-03	ATP5F1A,COL12A1,COL6A2,HSPA1A/ HSPA1B,KRT1,KRT6C,PRSS1,TPM2	8
Cancer, Gastrointestinal Disease, Hepatic System Disease, Liver Hyperplasia/Hyperproliferation, Organismal Injury and Abnormalities	Liver tumor	2.45E-03	ATP5F1A,BLMH,CLU,COL12A1,COL6A2, DSC3,ECM1,EEF2,FN1,HNRNPK,HSPA1A/ HSPA1B,KRT16,KRT6C,KRT77,PLEC, PRDX2,TPI1	17
Cancer, Gastrointestinal Disease, Hepatic System Disease, Liver Hyperplasia/Hyperproliferation, Organismal Injury and Abnormalities	Liver cancer	1.26E-02	ATP5F1A,CLU,COL12A1,COL6A2,DSC3, ECM1,EEF2,FN1,HNRNPK,PLEC,TPI1	11
Cancer, Gastrointestinal Disease, Hepatic System Disease, Liver Hyperplasia/Hyperproliferation, Organismal Injury and Abnormalities	Liver carcinoma	2.56E-02	ATP5F1A,COL12A1,COL6A2,DSC3,ECM1, EEF2,FN1,HNRNPK,PLEC,TPI1	10

Table S4. TRANSFAC-predicted transcription factors binding to the *dnah10* gene promoter region.

DNAH10_promoter_ENSR00000058778

>12 dna:chromosome chromosome:GRCh38:12:123761800:123762601:1
AAAGAACTCTGCTCCTTCCCTGATTTTGTAGGCCATGAGGAACTCGTGCTTTCTCGGCTTATTTCCTCCTCGTCTGGCCTCTAATCCCGCCTCTGCCACCGCCGAGGTGC
TGTGACCTTCGTCTCGCCAACTCTCTAGCCCCAGCTTCATCTGCAAGGTGGGCGGGCTGCTTGCAAACCGGTCGGCGTGGGGTGGGGTCGCACCCTCGGCTGCAGCGGCC
CGAGGGCGGCTCCTGGCGGCGAGGGTCCCGCTCCGCATCCAGGGGCGTGCGCGCTGCCGGGGCCAGGGAGCAACCAGCCTACCTCGGCGGCCCGGGGGCCTGGTGCGCGC
CTCCGAGATCCCGGGGCCGGGGCGTGGGCTGCGGCTCGCGGGTTCGGGATCGAGGGGCCGGGCGCGGGGCACTGCCGAGCTTGCAGCCCCCAGCGCCGGCGCCGTCGGCG
GGGCGAGCTGCGGCCGTTTCCGGGGCGCCCATTGCTAGGGCCGCCGTTGCCACGGACGCCCGCCCTGCGCCCGGCTCCCTCTGCACTGCGCGGCGCCATGGACGACCTGC
GGGTGCTGTGGATGCGCGACCGCGTGTATGCGGCTTTCGGCATCACCGACCCCCAGCTTTTCGAGGACCTGCTCAACCGCGACGACGGCCAGGGCGAGGACCTCATCTTG
CACTTCCTCAACCAGGCGAGCGAGGAGGAGGGGCCCTCGGCGCTCTTCATCTACCGCACTATGGTGCCGGAGGAGGTGGAGGTGGAGATTGGTGAGCCTCGACGCGCCGC
TCCCTTCCCCGGGCTTCCCTCCTGCCCGTCCC

Transcription factor (TF) counts

TF	count
AP-2 family	23
KLF-6	17
ZF5	15
SP-family	12
PAX-family	9
EGR-family	9
Kid3	8
E2F-family	8
SPI-family	8
KLF-family	8
FOX-family	8
Kaiso	5
ELF-family	5

Total sequence length : 802
Total number of sites : 277
Frequency of sites : 0.34539

Total number of sequences : 1
Number of sequences with sites : 1
Average number of sites per sequence: 277.00

Matrix library : TRANSFAC MATRIX TABLE, Release 2017.3
Sequence file : DNAH10_promoter_ENSR00000058778
Profile : vertebrates.prf (details)
Only high-quality matrices : yes
Cut-offs : Minimize false positives

Matrix	Factor name	Position (strand)	Core score	Matrix score	Sequence
V$SZF11_01	SZF1-1	9 (-)	1.000	0.844	ctgctccttCCCTGa
V$SPIB_Q3	Spi-B	37 (-)	1.000	1.000	gAGGAA
V$MAX_01	Max	39 (+)	0.800	0.843	ggaaCTCGTgcttt
V$MAX_01	Max	39 (-)	0.800	0.843	ggaacTCGTGcttt
V$HES1_02	Hes1	41 (-)	0.965	0.963	aacTCGTGct
V$BCL6_02	BCL-6	47 (+)	1.000	0.878	tgCTTTCtcggctt
V$PAX6_01	Pax-6	47 (+)	0.812	0.792	tgcttTCTCGgcttatttcct
V$IRF4_04	IRF4 secondary motif	47 (+)	1.000	0.949	tgcttTCTCGgctta
V$SPIB_Q3	Spi-B	63 (+)	1.000	1.000	TTCCTc
V$ZBTB2_Q3_01	ZBTB2	64 (+)	1.000	0.987	tccTCCTCg
V$SMAD3_03	Smad3	66 (-)	1.000	0.953	ctcctcGTCTGgcctct
V$PITX1_Q6	PITX1	77 (+)	1.000	0.947	gcctcTAATCc
V$PITX3_Q3_01	Pitx3	77 (-)	1.000	0.999	gcctcTAATCc
V$PITX1_01	Pitx1	77 (-)	1.000	0.931	gcctctAATCCcgcctc
V$CRX_Q4_02	Crx	80 (+)	1.000	1.000	tcTAATCcc
V$PITX3_Q3	Pitx3	80 (-)	1.000	1.000	tcTAATC
V$PITX3_03	PITX3	80 (+)	1.000	0.998	tcTAATCcc
V$GSC2_01	GSC2	80 (+)	1.000	0.992	tcTAATCccg
V$DPRX_01	DPRX	80 (-)	1.000	0.993	tcTAATCccg
V$DMBX1_02	DMBX1	80 (-)	1.000	0.996	tcTAATCccg
V$TCF1_07	TCF1 secondary motif	80 (-)	1.000	0.992	tcTAATCccgcctc
V$GTF2IRD1_01	GTF2IRD1-isoform2	80 (-)	1.000	0.985	tcTAATCcc
V$CRX_Q4_01	CRX	81 (+)	1.000	1.000	cTAATC
V$RHOXF1_02	RHOXF1	81 (+)	1.000	0.999	ctAATCCc
V$E2F4_05	E2F-4	82 (+)	1.000	0.936	taatCCCGCctc
V$SREBP2_Q6	SREBP-2	93 (+)	1.000	0.988	ctGCCACcgccg
V$KID3_01	Kid3	96 (+)	1.000	1.000	CCACC
V$EP300_05	p300	98 (+)	0.793	0.870	ACCGCcg
V$SIN3A_01	sin3A	99 (+)	1.000	0.803	ccgccgagGTGCTg
V$REST_Q5	REST	102 (+)	1.000	0.968	ccgaggTGCTGtg
V$DAX1_01	DAX1	107 (-)	1.000	0.991	gtgctgtGACCTtcgtctcg
V$COUPTF2_Q6	COUP-TF2	109 (-)	1.000	0.969	gctgTGACCttcgtc
V$RXRA_04	RXR-ALPHA secondary motif	109 (-)	1.000	0.920	gctgtgACCTTcgtct
V$ZBTB44_01	Zbtb44	110 (+)	1.000	1.000	CTGTGa
V$NR4A2_01	NURR1	112 (-)	1.000	1.000	gTGACCtt
V$COUPTF1_Q6_01	COUP-TF1	113 (+)	1.000	1.000	TGACCtt
V$RARG_Q3	RAR-gamma	113 (+)	1.000	0.995	TGACCttcgtc
V$NR1B1_01	NR1B1	113 (-)	1.000	1.000	TGACCtt
V$RXRA_12	RXR-ALPHA	113 (-)	1.000	1.000	TGACCtt
V$EP300_05	p300	115 (+)	0.815	0.883	ACCTTcg
V$PAX3_01	Pax-3	119 (+)	1.000	0.827	TCGTCtcgccaac
V$ZBTB33_01	Kaiso	122 (+)	0.795	0.814	tcTCGCCaa
V$CHD2_01	CHD2	122 (+)	0.793	0.804	tcTCGCCaa
V$CPBP_Q6	CPBP	138 (+)	1.000	1.000	GCCCCag
V$NMYC_02	NMYC	148 (+)	1.000	1.000	CATCTg
V$HSF4_Q3	HSF4	151 (+)	1.000	1.000	CTGCAag
V$EGR3_Q6	EGR3	154 (-)	1.000	0.965	caagGTGGGcgggc
V$KID3_01	Kid3	157 (-)	1.000	1.000	GGTGG
V$ZF5_B	ZF5	157 (+)	0.888	0.906	ggtgggCGGGCtg
V$LKLF_02	LKLF	159 (+)	1.000	1.000	tGGGCGg
V$KLF17_01	KLF17	159 (+)	1.000	1.000	tgGGCGG

V$KLF17_02	Klf17	159 (+)	1.000	1.000	tgGGCGG
V$GABPA_08	GABP-alpha	176 (+)	1.000	0.925	AACCGgtcg
V$RREB1_01	RREB-1	184 (-)	0.901	0.840	ggcgtgggGTGGGg
V$EGR1_17	Egr-1	185 (+)	1.000	0.942	GCGTGgggtg

Table S4. Continued. TRANSFAC-predicted transcription factors binding to the *dnah10* gene promoter region.

Matrix	Factor name	Position (strand)	Core score	Matrix score	Sequence
V$KID3_01	Kid3	186 (-)	1.000	1.000	CGTGG
V$ETF_Q6_01	ETF	187 (-)	0.965	0.954	gtggGGTGGgg
V$SREBP1_Q6	SREBP-1	188 (-)	1.000	1.000	tgGGGTG
V$GKLF_03	GKLF	189 (+)	1.000	0.961	gGGGTGggtcg
V$LKLF_Q6	LKLF	189 (-)	1.000	1.000	gGGGTGggg
V$LKLF_Q3	LKLF	189 (+)	1.000	0.998	gGGGTGgggt
V$PAX4_03	Pax-4	189 (-)	1.000	0.986	gGGGTGggtcg
V$SALL2_01	SALL2	190 (+)	1.000	1.000	GGGTGgg
V$KLF_Q3	KLF	190 (+)	1.000	1.000	GGGTGggg
V$KID3_01	Kid3	191 (-)	1.000	1.000	GGTGG
V$AP2BETA_Q3	AP-2beta	212 (+)	0.990	0.989	gcagcggcccGAGGGc
V$BEN_01	BEN	213 (+)	1.000	0.996	CAGCGgcc
V$TFAP2A_10	TFAP2A	214 (+)	1.000	0.929	agcggcccgAGGGCg
V$TCFAP2C_04	TCFAP2C secondary motif	216 (+)	0.968	0.969	cggcccgaGGGCGg
V$TCFAP2B_03	Tcfap2b	216 (+)	0.939	0.950	cggcccgaGGGCGg
V$TCFAP2C_04	TCFAP2C secondary motif	216 (-)	0.956	0.953	cGGCCCgagggcgg
V$TFAP2C_01	TFAP2C	217 (+)	0.978	0.965	GGCCCgagggcg
V$AP2_Q6_01	AP-2	217 (-)	0.986	0.986	gGCCCGagggcgg
V$AP2_Q6	AP-2	217 (+)	0.942	0.943	ggCCCGAgggcg
V$AP2_Q6	AP-2	217 (-)	0.953	0.940	ggcccGAGGGcg
V$TFAP2A_02	TFAP2A	217 (-)	0.986	0.982	ggcccgaGGGCG
V$TFAP2C_01	TFAP2C	217 (-)	0.990	0.961	ggcccgaGGGCG
V$AP2GAMMA_Q5_01	AP-2gamma	218 (-)	0.982	0.986	gcccgAGGGC
V$AP2GAMMA_01	AP-2gamma	218 (+)	1.000	0.998	GCCCGaggg
V$EGR2_04	Egr-2	220 (+)	1.000	0.849	ccgaGGGCGg
V$CTCF_18	ctcf	223 (-)	0.921	0.853	agggcggCTCCTggcggcga
V$SP6_02	SP6	223 (+)	1.000	1.000	agGGCGG
V$SP6_01	Sp6	223 (+)	1.000	1.000	agGGCGG
V$SP3_02	Sp3	223 (+)	1.000	1.000	aGGGCGg
V$CPBP_02	CPBP	223 (+)	1.000	1.000	aGGGCGg
V$LKLF_01	LKLF	223 (+)	1.000	1.000	aGGGCGg
V$SP2_05	Sp2	223 (+)	1.000	1.000	aGGGCGg
V$CPBP_01	CPBP	223 (+)	1.000	1.000	aGGGCGg
V$CTCF_04	CTCF	225 (+)	0.813	0.824	gGCGGCtcctggcggcg
V$LRF_Q6	LRF	237 (+)	0.994	0.981	cggcGAGGGtcc
V$PAX3_B	Pax-3	238 (+)	0.818	0.820	ggcgagggTCCCGctccgcat
V$PAX5_02	Pax-5	239 (-)	0.973	0.773	gcgagggtccCGCTCcgcatccaggggc
V$E2F1_09	E2F-1	244 (-)	1.000	0.916	ggtCCCGCtcc
V$FOXN1_01	FOXN1	244 (-)	0.840	0.787	ggtcccgctccGCATCcaggggc
V$CPBP_Q6	CPBP	260 (-)	1.000	1.000	caGGGGC
V$ZFP161_04	ZF5 secondary motif	262 (-)	1.000	0.866	ggggcgtGCGCGct
V$AHRHIF_Q6	AhR, Arnt, HIF-1	264 (+)	1.000	0.997	gGCGTGcgc
V$EGR1_15	Egr-1	264 (+)	1.000	0.985	ggcGTGCGc
V$ZF5_B	ZF5	264 (+)	1.000	0.962	ggcgtgCGCGCtg
V$ZF5_B	ZF5	267 (-)	1.000	0.877	gtGCGCGctgccg
V$ZF5_01	ZF5	269 (+)	1.000	0.926	GCGCGctg
V$BEN_01	BEN	269 (-)	1.000	0.951	gcgCGCTG
V$HSF4_Q3	HSF4	274 (+)	1.000	1.000	CTGCCgg
V$CPBP_Q6	CPBP	277 (-)	1.000	1.000	ccGGGGC
V$PUR1_Q4	PUR1	280 (+)	1.000	0.999	ggGCCAGgg
V$REST_13	REST	282 (-)	0.797	0.738	gccagGGAGCaaccagc
V$CP2_02	CP2/LBP-1c/LSF	284 (-)	1.000	0.959	cagggagcaaCCAGC
V$AP2_Q3	AP-2	304 (-)	1.000	0.944	tcggcgGCCCGggggc
V$TFAP2C_01	TFAP2C	309 (+)	0.978	0.953	GGCCCgggggcc
V$AP2_Q6	AP-2	309 (-)	0.992	0.952	ggcccGGGGGcc
V$TFAP2C_01	TFAP2C	309 (-)	0.978	0.956	ggcccggGGGCC
V$AP2GAMMA_Q5_01	AP-2gamma	310 (-)	0.982	0.986	gcccgGGGGC
V$AP2ALPHA_Q6	AP-2alpha	310 (-)	1.000	0.993	gcccgGGGGCc
V$AP2_Q4	AP2	310 (+)	1.000	0.987	GCCCGggggc
V$AP2GAMMA_01	AP-2gamma	310 (+)	1.000	1.000	GCCCGgggg
V$AP2ALPHA_01	AP-2alpha	310 (+)	1.000	1.000	GCCCGgggg
V$CPBP_Q6	CPBP	312 (-)	1.000	1.000	ccGGGGG
V$CHCH_01	Churchill	313 (+)	1.000	1.000	CGGGGg
V$ZF5_B	ZF5	323 (-)	1.000	0.913	gtGCGCGcctccg
V$E2F_Q2	E2F	326 (-)	1.000	1.000	cGCGCC
V$IRF4_04	IRF4 secondary motif	329 (-)	1.000	0.931	gcctcCGAGAtcccg
V$SP3_Q3	Sp3	338 (+)	0.944	0.904	atcccgggGCCGGg
V$CPBP_Q6	CPBP	341 (-)	1.000	1.000	ccGGGGC
V$ZAC_04	ZAC	343 (+)	1.000	1.000	gGGGCCg
V$KLF15_Q2	KLF15	344 (+)	1.000	0.984	gggccGGGGCgtgg
V$CPBP_Q6	CPBP	347 (-)	1.000	1.000	ccGGGGC
V$GKLF_Q3_01	GKLF	347 (+)	1.000	0.997	ccggggcGTGGGc
V$SP3_01	SP3	348 (-)	1.000	0.967	cgGGGCGtggg
V$CACD_01	CACD	350 (-)	0.983	0.988	GGGCGtgg
V$EGR1_15	Egr-1	351 (+)	0.870	0.887	ggcGTGGGc
V$EGR2_04	Egr-2	352 (+)	0.800	0.894	gcgtGGGCTg
V$EGR1_17	Egr-1	352 (+)	1.000	0.947	GCGTGggctg
V$KID3_01	Kid3	353 (-)	1.000	1.000	CGTGG
V$ZBED6_01	ZBED6	361 (+)	1.000	0.950	gcGGCTCgcggg
V$CHD2_01	CHD2	364 (+)	1.000	0.777	gcTCGCGgg
V$CHD2_01	CHD2	365 (-)	0.793	0.777	ctCGCGGgt
V$ZNF777_02	ZNF777	375 (-)	0.832	0.751	cgggatcgAGGGGccgggc
V$CPBP_Q6	CPBP	382 (-)	1.000	1.000	gaGGGGC
V$PLAG1_02	PLAG1	384 (-)	0.900	0.902	ggggccgggcgCGGGG
V$FOXN1_01	FOXN1	384 (+)	0.789	0.760	ggggccgGGCGCggggcactgcc
V$ZAC_04	ZAC	384 (+)	1.000	1.000	gGGGCCg
V$ZFP161_04	ZF5 secondary motif	385 (-)	1.000	0.891	gggccggGCGCGgg
V$KAISO_Q2	Kaiso	387 (+)	0.902	0.955	gccgggCGCGGg
V$ZF5_01	ZF5	390 (+)	1.000	1.000	GGGCGcgg
V$AP2ALPHA_Q6	AP-2alpha	391 (-)	1.000	0.989	ggcgcGGGGCa
V$E2F_Q2	E2F	391 (+)	1.000	1.000	GGCGCg
V$CPBP_Q6	CPBP	394 (-)	1.000	1.000	gcGGGGC
V$EP300_05	p300	401 (+)	0.795	0.871	ACTGCcg
V$REST_13	REST	409 (+)	1.000	0.733	gcttgcaGCCCCcagcg
V$HSF4_Q3	HSF4	410 (-)	1.000	1.000	ctTGCAG
V$KAISO_Q2	Kaiso	413 (-)	0.913	0.927	gCAGCCcccagc

V$AP2ALPHA_Q6	AP-2alpha	415 (+)	1.000	0.993	aGCCCCcagcg
V$ZNF300_04	ZNF300	415 (-)	1.000	0.990	agcCCCCAg
V$CPBP_Q6	CPBP	417 (+)	1.000	1.000	CCCCCag
V$EGR1_11	Egr-1	423 (+)	0.969	0.975	GCGCCggcg
V$EGR1_11	Egr-1	424 (-)	0.969	0.975	cgccGGCGC
V$AP2ALPHA_03	AP-2alphaA	428 (+)	0.901	0.874	ggCGCCGtcggcggg
V$AP2ALPHA_03	AP-2alphaA	428 (-)	0.901	0.874	ggcgccgtCGGCGgg
V$E2F3_Q6	E2F-3	437 (+)	1.000	1.000	GGCGGgg

Table S4. Continued. TRANSFAC-predicted transcription factors binding to the *dnah10* gene promoter region.

Matrix	Factor name	Position (strand)	Core score	Matrix score	Sequence
V$ZF5_B	ZF5	438 (+)	0.888	0.866	gcggggCGAGCtg
V$CPBP_Q6	CPBP	438 (-)	1.000	1.000	gcGGGGC
V$RREB1_06	RREB-1	440 (+)	0.969	0.978	GGGGCga
V$ZBED6_01	ZBED6	440 (-)	0.961	0.946	ggggcGAGCTgc
V$DEAF1_02	DEAF1	444 (+)	0.814	0.864	cgagcTGCGGccgtttccggggcgc
V$DEAF1_01	DEAF1	444 (+)	1.000	0.846	cgagctgcggccgtTTCCGgggcgc
V$EP300_05	p300	451 (-)	0.790	0.867	cgGCCGT
V$VMYB_02	v-Myb	453 (-)	1.000	0.938	gCCGTTtcc
V$ELK1_02	Elk-1	454 (-)	1.000	0.968	ccgtTTCCGgggcg
V$RFX1_02	RFX1	454 (-)	0.982	0.938	ccGTTTCcggggcgccca
V$ELK1_06	ELK-1	455 (-)	1.000	0.904	cgtTTCCGgg
V$ER81_02	ER81	455 (-)	1.000	0.966	cgtTTCCGgg
V$ER71_02	ER71	455 (-)	1.000	0.969	cgtTTCCGgg
V$TEL1_02	TEL1	455 (-)	1.000	0.996	cgtTTCCGgg
V$ETV7_01	ETV7	455 (-)	1.000	1.000	cgtTTCCGgg
V$GABPA_02	GABPalpha	455 (-)	1.000	0.974	cgtTTCCGgg
V$FLI1_03	FLI1	455 (-)	1.000	0.882	cgtTTCCGgg
V$FLI1_06	FLI-1	455 (+)	1.000	0.942	cgtTTCCGgg
V$CETS1_01	c-ets-1 (ets1)	455 (-)	1.000	0.942	cgttTCCGGg
V$ETS1_02	ETS1	455 (-)	1.000	0.941	cgttTCCGGg
V$ELF4_02	ELF4	455 (-)	1.000	0.970	cgtTTCCGgg
V$ELF_02	ELF1	455 (-)	1.000	0.959	cgtTTCCGgg
V$EHF_02	EHF	455 (-)	0.989	0.959	cgTTTCCggg
V$ELF5_02	Elf5	455 (-)	0.996	0.976	cgTTTCCggg
V$ELF5_03	Elf5	455 (-)	0.996	0.992	cgTTTCCggg
V$ESE1_01	ESE-1	455 (-)	1.000	0.978	cgtTTCCGgg
V$ELF4_01	ELF4	455 (-)	1.000	0.976	cgtTTCCGgg
V$ELK1_05	ELK-1	455 (-)	1.000	0.918	cgtTTCCGgg
V$PEA3_01	PEA3	455 (-)	1.000	0.954	cgtTTCCGgg
V$TEL1_01	TEL1	455 (-)	1.000	0.996	cgtTTCCGgg
V$GABPA_01	GABPalpha	455 (-)	1.000	0.967	cgtTTCCGgg
V$EHF_03	EHF	455 (-)	1.000	0.970	cgtTTCCGgg
V$PLAGL1_03	Plagl1	459 (+)	1.000	0.979	tccggGGCGCccattg
V$PLAGL1_03	Plagl1	459 (-)	1.000	0.984	tccgggGCGCCcattg
V$ZF5_B	ZF5	460 (+)	0.919	0.858	ccggggCGCCCat
V$CPBP_Q6	CPBP	460 (-)	1.000	1.000	ccGGGGC
V$SOX18_Q5	Sox-18	468 (-)	1.000	1.000	ccCATTG
V$RFX1_05	rfx1	480 (+)	1.000	0.949	gccgccGTTGCcacggacgccc
V$VMYB_02	v-Myb	483 (-)	1.000	0.984	gCCGTTgcc
V$RFX_Q6	RFX	484 (+)	1.000	0.994	ccGTTGCca
V$RFX_Q6_01	RFX	484 (+)	1.000	0.991	ccGTTGCcacgga
V$RFX1_04	rfx1	486 (-)	1.000	0.931	GTTGCcacggacgc
V$RFX1_Q6	Rfx1	486 (+)	1.000	0.947	GTTGCcacggacgc
V$KID3_01	Kid3	490 (+)	1.000	1.000	CCACG
V$EP300_05	p300	496 (+)	0.811	0.881	ACGCCcg
V$SP3_02	Sp3	500 (-)	1.000	1.000	cCGCCCt
V$CPBP_02	CPBP	500 (-)	1.000	1.000	cCGCCCt
V$LKLF_01	LKLF	500 (-)	1.000	1.000	cCGCCCt
V$SP2_05	Sp2	500 (-)	1.000	1.000	cCGCCCt
V$CPBP_01	CPBP	500 (-)	1.000	1.000	cCGCCCt
V$SP6_02	SP6	500 (-)	1.000	1.000	CCGCCct
V$SP6_01	Sp6	500 (-)	1.000	1.000	CCGCCct
V$KAISO_Q2	Kaiso	504 (-)	0.931	0.968	cCTGCGcccggc
V$ZF5_01	ZF5	505 (-)	1.000	0.926	ctgCGCCC
V$FOXN1_01	FOXN1	519 (-)	0.791	0.775	ctctgcactgcGCGGCgccatgg
V$ZFP161_04	ZF5 secondary motif	521 (-)	1.000	0.999	ctgcactGCGCGgc
V$E2F2_04	E2F-2 secondary motif	527 (+)	1.000	0.957	tgcgcGGCGCcatggac
V$YY1_06	YY1	532 (+)	1.000	0.953	ggcGCCATgga
V$EP300_05	p300	545 (+)	0.793	0.870	ACCTGcg
V$FOXN4_04	FOXN4_2ndary	561 (+)	1.000	0.851	gatgcgcgacCGCGTgtatgcg
V$ZF5_B	ZF5	562 (-)	1.000	0.888	atGCGCGaccgcg
V$E2F1_Q3	E2F-1	564 (-)	1.000	0.974	gCGCGAcc
V$HES1_02	Hes1	569 (-)	1.000	0.983	accGCGTGta
V$NR2C2_04	TR4	599 (+)	1.000	0.871	ACCCCca
V$CPBP_Q6	CPBP	600 (+)	1.000	1.000	CCCCCag
V$SALL1_04	Sall1	609 (-)	1.000	0.948	tttcgaGGACCt
V$SALL1_02	Sall1	609 (-)	1.000	0.948	tttcgaGGACCt
V$DR3_Q4	VDR, CAR, PXR	612 (+)	0.941	0.857	cgaggacctgcTCAACcgcga
V$PAX5_02	Pax-5	612 (+)	0.738	0.754	cgaggacctgctcAACCGcgacgacggc
V$FOXN4_04	FOXN4 secondary motif	618 (+)	0.818	0.759	cctgctcaacCGCGAcgacggc
V$PAX3_B	Pax-3	620 (-)	0.810	0.853	tgctcaacCGCGAcgacggcc
V$GTF3C2_01	TF3C-beta	622 (-)	0.650	0.715	ctCAACCgcgacgacggccagg
V$PAX3_01	Pax-3	623 (-)	1.000	0.823	tcaaccgcGACGA
V$GABPA_08	GABP-alpha	625 (+)	1.000	0.916	AACCGcgac
V$GTF3C2_01	TF3C-beta	627 (+)	0.641	0.725	ccgcgacgacggccaGGGCGag
V$MEQCJUN_02	MEQ:c-Jun	647 (-)	0.906	0.837	agGACCTcatc
V$PRDM16_04	MEL1	652 (-)	1.000	1.000	cTCATC
V$ZNF35_04	ZNF35	656 (-)	1.000	1.000	TCTTGc
V$ETS1_B	c-Ets-1	656 (-)	1.000	0.934	tcttgcacTTCCTca
V$DR3_Q4	VDR, CAR, PXR	657 (+)	0.941	0.872	cttgcacttccTCAACcaggc
V$TFIII_Q6_01	TFII-I	660 (-)	1.000	0.992	gcacTTCCTc
V$SPI1_03	SPI1	661 (-)	1.000	0.998	caCTTCCtca
V$SPIC_02	Spic	661 (-)	1.000	1.000	caCTTCCtca
V$SPIB_02	Spi-B	661 (-)	1.000	0.977	caCTTCCtca
V$PEA3_Q6	PEA3	662 (+)	1.000	1.000	aCTTCCt
V$ETS_Q6	Ets	662 (+)	1.000	1.000	aCTTCCt
V$CETS1_Q6	C-ets-1	662 (-)	1.000	1.000	aCTTCCt
V$ELK1_Q6	Elk-1	662 (-)	1.000	1.000	aCTTCC
V$FLI1_Q5	Fli-1	662 (-)	1.000	1.000	aCTTCCt
V$SPI1_04	SPI1	662 (-)	1.000	1.000	acTTCCT
V$PEA3_Q6_02	PEA3	662 (-)	1.000	1.000	acTTCCT
V$SPI1_Q5	PU.1	663 (-)	1.000	1.000	cTTCCT
V$ELF1_Q5	Elf-1	663 (-)	1.000	1.000	cTTCCT
V$SPIB_Q3	Spi-B	664 (+)	1.000	1.000	TTCCTc
V$PAX6_05_01	Pax-6	665 (-)	0.957	0.765	tcctcaaccAGGCGagcga
V$SP1_08	Sp1	680 (+)	0.945	0.936	gcgaGGAGGagggg

V$ZBTB2_Q3_01	ZBTB2	681 (-)	1.000	0.987	cGAGGAgga
V$CPBP_Q6	CPBP	688 (-)	1.000	1.000	gaGGGGC
V$ZF5_01	ZF5	698 (-)	0.961	0.900	cggCGCTC
V$ZNF177_01	ZNF177	701 (-)	1.000	0.971	cgcTCTTCatct
V$ZFP536_02	Zfp536	727 (+)	1.000	1.000	cCGGAGg
V$ZNF263_02	FPM315	729 (+)	1.000	0.918	gGAGGAggtggaggtggagat
V$TFIII_Q6_01	TFII-I	730 (+)	0.984	0.973	gAGGAGgtgg
V$KID3_01	Kid3	735 (-)	1.000	1.000	GGTGG

**Matrix**	**Factor name**	**Position (strand)**	**Core score**	**Matrix score**	**Sequence**
V$KID3_01	Kid3	741 (-)	1.000	1.000	GGTGG
V$YB1_Q3	YB-1	743 (-)	1.000	0.966	tggagATTGG
V$HOXA7_01	HOXA7	746 (-)	1.000	1.000	aGATTGg
V$FOXN1_01	FOXN1	753 (-)	0.789	0.765	tgagcctcgacGCGCCgctccct
V$FOXN1_01	FOXN1	754 (+)	1.000	0.910	gagcctcGACGCgccgctccctt
V$FOXN4_04	FOXN4_2ndary	755 (-)	1.000	0.792	agcctcgACGCGccgctccctt
V$E2F_Q2	E2F	763 (-)	1.000	1.000	cGCGCC
V$ZF5_01	ZF5	767 (-)	0.844	0.896	ccgCTCCC
V$IRF3_06	IRF3_2ndary	767 (-)	0.975	0.920	ccgctCCCTTcccc
V$IRF4_07	IRF-4	771 (-)	1.000	0.970	tccCTTCCc
V$SP3_Q3	Sp3	772 (-)	0.962	0.926	cCCTTCcccgggct
V$KAISO_Q2	Kaiso	773 (-)	0.938	0.923	cCTTCCccgggc
V$SPIB_Q3	Spi-B	775 (+)	1.000	1.000	TTCCCc
V$HSF_Q6	HSF	775 (+)	0.976	0.857	ttccccggGCTTC

Table S5. Transcripts produced from the DNAH10 gene ENSG00000197653.

Name	Transcript ID	Exons	Known variations	bp	Protein	Molecular mass (g/mol)	Isoelectric point	Charge	Translation ID	Biotype	CCDS	UniProt	RefSeq	Flags
DNAH10-210	ENST00000638045.1	78	5.175	13.678	4,471aa	514,840.64	5.6602	-46.5	ENSP00000489675	Protein coding	CCDS9255	Q8IVF4	NM_207437 NP_997320	TSL:5GENCODE basicAPPRIS P2
DNAH10-201	ENST00000409039.7	78	5.194	14.473	4,532aa	521,940.56	5.5432	-54.0	ENSP00000386770	Protein coding	-	A0A1C7CYW8	-	TSL:5GENCODE basicAPPRIS ALT2
DNAH10-209	ENST00000614082.1	20	1.134	3.484	970aa	113,324.25	6.9217	6.5	ENSP00000479072	Protein coding	-	A0A087WV07	-	TSL:5GENCODE basic
DNAH10-207	ENST00000540041.2	5	310	1.003	319aa	36,283.53	6.2835	-0.5	ENSP00000445308	Protein coding	-	H0YGZ2	-	CDS 3' incompleteTSL:5
DNAH10-205	ENST00000497783.3	21	870	3.890	648aa	73,713.07	5.6665	-4.5	ENSP00000444761	Nonsense-mediated decay	-	F5H515	-	CDS 5' incompleteTSL:2
DNAH10-206	ENST00000538983.1	4	103	448	81aa	9,456.08	10.3018	3.5	ENSP00000485079	Nonsense-mediated decay	-	A0A096LNK1	-	CDS 5' incompleteTSL:3
DNAH10-203	ENST00000467219.5	-	-	3.422	No protein	-	-	-	-	Retained intron	-	-	-	TSL:2
DNAH10-202	ENST00000447853.2	-	-	3.211	No protein	-	-	-	-	Retained intron	-	-	-	TSL:1
DNAH10-204	ENST00000492261.5	-	-	2.793	No protein	-	-	-	-	Retained intron	-	-	-	TSL:2
DNAH10-208	ENST00000545078.1	-	-	721	No protein	-	-	-	-	Retained intron	-	-	-	TSL:3

Table S6.

Small-molecular weight transcripts from the *dnah10* gene as predicted by TRANSFAC analysis.

dnah10-gene-translated protein sequences of alternative transcripts with translated protein products Mw less than 120kDa.

DNAH10-209; Mw 113,324.25 g/mol; length 970 aa [Unique peptide not found in sequence]

>tr|A0A087WV07|A0A087WV07_HUMAN Dynein heavy chain 10, axonemal OS=Homo sapiens OX=9606 GN=DNAH10 PE=1 SV=1
MPGEAVEYHSIQLIRDEFLMNVQKFASNIQRTMQQLEGEIKLEMPIISVEGEVSDLAADPETVDILEQCVINWLNQISTAVEAQLKKTPQGKGPLAEIEFWRERNATLSALHEQTKLPIVRKVLDVIKESDSMLVANLQPVFTELFKFHTEASDNVRFLSTVERYFKNITHGSGFHVVLDTIPAMMSA
LRMVWIISRHYNKDERMIPLMERIAWEIAERVCRVVNLRTLFKENRASAQSKTLEARNTLRLWKKAYFDTRAKIEASGREDRWEFDRKRLFERTDYMATICQDLSDVLQILEEFYNIFGPELKAVTGDPKRIDDVLCRVDGLVTPMENLTFDPFSIKSSQFWKYVMDEFKIEVLVIEKEAKHFIDESFK
TLRSAEAAFDMLLKFKHIRSREAVNRQMMMKFNDILAQYCKEIDIINKIFVQNLENPPLYKNHPPVAGAIYWERSLFFRIKHTILRFQEVQEILDSDRGQEVKQKYLEVGRTMKEYEDRKYEQWMEVTEQVLPALMKKSLLTKSSIATEEPSTLERGAVFAINFSPALREIINETKYLEQLGFTVPELAR
NVALQEDKFLRYTAGIQRMLDHYHMLIGTLNDAESVLLKDHSQELLRVFRSGYKRLNWNSLGIGDYITGCKQAIGKFESLVHQIHKNADDISSRLTLIEAINLFKYPAAKSEEELPGVKEFFEHIERERASDVDHMVRWYLAIGPLLTKVEGLVVHTNTGKAPKLASYYKYWEKKIYEVLTKLILKNLQSF
NSLILGNVPLFHTETILTAPEIILHPNTNEIDKMCFHCVRNCVEITKHFVRWMNGSCIECPPQKGEEEEVVIINFYNDISLNPQIIEQAVMIPQNVHRILINLMKYLQKWKRYRPLWKLDKAIVMEKFAAKKPPCVAYDEKLQFYSKIAYEVMRHPLIKDEHCIRLQLRHLANTVQENAKSWVISLGKLL
NESAKEELYNLHEEMEVLNRCV

DNAH10-207; Mw 36,283.53 g/mol; length 319 aa [Unique peptide not found in sequence]

>tr|H0YGZ2|H0YGZ2_HUMAN Dynein heavy chain 10, axonemal (Fragment) OS=Homo sapiens OX=9606 GN=DNAH10 PE=1 SV=2
MPGPLPGRPHTASMPSGTSVSCSSLLLFLPLLLCLVCAVLFHFCLSMAWSGRLLWLSLTLPGSGMPRSFAKPPKQVQTVCECILIMKGYKELNWKTAKGVMSDPNFLRSLMEIDFDSITQSQVKNIKGLLKTLNTTTEEMEAVSKAGLGMLKFVEAVMGYCDVFREIKPKREKVARLERNFYLTKRE
LERIQNELAAIQKELETLGAKYEAAILEKQKLQEEAEIMERRLIAADKLISGLGSENIRWLNDLDELMHRRVKLLGDCLLCAAFLSYEGAFTWEFRDEMVNRIWQNDILEREIPLSQPFRLESLLTDDVEIS

DNAH10-205; Mw 73,713.07 g/mol; length 648 aa [Unique peptide at aa 1-28]

>tr|F5H515|F5H515_HUMAN Dynein heavy chain 10, axonemal (Fragment) OS=Homo sapiens OX=9606 GN=DNAH10 PE=1 SV=2
LESIFIGGDIRSQLPEEAKKFDNIDKVFKRIMGETLKDPVIKRCCEAPNRLSDLQNVSEGLEKCQKSLNDYLDSKRNAFPRFFFISDDELLSILGSSDPLCVQEHMIKMYDNIASLRFNDGDSGEKLVSAMISAEGEVMEFRKILRAEGRVEDWMTAVLNEMRRTNRLITKEAIFRYCEDRSRVDWMLL
YQGMVVLAASQVWWTWEVEDVFHKAQKGEKQAMKNYGRKMHRQIDELVTRITMPLSKNDRKKYNTVLIIDVHARDIVDSFIRGSILEAREFDWESQLRFYWDREPDELNIRQCTGTFGYGYEYMGLNGRLVITPLTDRIYLTLTQALSMYLGGAPAGPAGTGKTETTKDLAKALGLLCVVTNC
GEGMDYRAVGKIFSGLAQCGAWGCFDEFNRIDASVLSVISSQIQTIRNALIHQLTTFQFEGQEISLDSRMGIFITMNPGYAGRTELPESVKALFRPVVVIVPDLQQICEIMLFSEGFLEAKTLAKKMTVLYKLAREQLSKQYHYDFGLRALKSVLVMAGELKRGSSDLREDVVLMRALRDMNLPKFVF
EDVPLFLGLISDLFPGLDCPRVRYPDFNDAVEQVLEENGYAVLPIQVDKVVQMFETMLTRHTTMVVGPTRGGKSVVINTLCQAQTNLS

DNAH10-206; Mw 9,456.08 g/mol; length 81 aa [Unique peptide not found in sequence]

>tr|A0A096LNK1|A0A096LNK1_HUMAN Dynein heavy chain 10, axonemal (Fragment) OS=Homo sapiens OX=9606 GN=DNAH10 PE=1 SV=1
ERFNKLVVRMTKSLAELQRALAGEVGMSNELDDVARSLFIGHIPNIWRRLAPDTLKSLGNWMVYFLRRFSQYMLWLLLDGS

DNAH10-201; 521,940.56 g/mol; length 4,532 aa [Unique peptide at aa 1548-1575]

>tr|A0A1C7CYW8|A0A1C7CYW8_HUMAN Dynein heavy chain 10, axonemal OS=Homo sapiens OX=9606 GN=DNAH10 PE=1 SV=1
MDDLRVLWMRDRVYAAFGITDPQLFEDLLNRDDGQGEDLILHFLNQASEEEGPSALFIYRTMVPEEVEVEIDEIPVLSEEGEEEEETYSQKVESVDKVRAKRVSLRTESLGQPLNREDEEMDKEISEKLPSKRTAKHIMEKMHLHMLCTPLPEEFLDQNVVFFLRNTKEAISEATDMKEAMEIMPET
LEYGIINANVLHFLKNIICQVFLPALSFNQHRTSTTVGVTSGEVSNSSEHESDLPPMPGEAVEYHSIQLIRDEFLMNVQKFASNIQRTMQQLEGEIKLEMPIISVEGEVSDLAADPETVDILEQCVINWLNQISTAVEAQLKKTPQGKGPLAEIEFWRERNATLSALHEQTKLPIVRKVLDVIKESDSML
VANLQPVFTELFKFHTEASDNVRFLSTVERYFKNITHGSGFHVVLDTIPAMMSALRMVWIISRHYNKDERMIPLMERIAWEIAERVCRVVNLRTLFKENRASAQSKTLEARNTLRLWKKAYFDTRAKIEASGREDRWEFDRKRLFERTDYMATICQDLSDVLQILEEFYNIFGPELKAVTGDPKRIDD
VLCRVDGLVTPMENLTFDPFSIKSSQFWKYVMDEFKIEVLIDIINKIFVQNLENPPLYKNHPPVAGAIYWERSLFFRIKHTILRFQEVQEILDSDRGQEVKQKYLEVGRTMKEYEDRKYEQWMEVTEQVLPALMKKSLLTKSSIATEEPSTLERGAVFAINFSPALREIINETKYLEQLGFTVPELARNVA
LQEDKFLRYTAGIQRMLDHYHMLIGTLNDAESVLLKDHSQELLRVFRSGYKRLNWNSLGIGDYITGCKQAIGKFESLVHQIHKNADDISSRLTLIEAINLFKYPAAKSEEELPGVKEFFEHIERERASDVDHMVRWYLAIGPLLTKVEGLVVHTNTGKAPKLASYYKYWEKKIYEVLTKLILKNLQSFNSLI
LGNVPLFHTETILTAPEIILHPNTNEIDKMCFHCVRNCVEITKHFVRWMNGSCIECPPQKGEEEEVVIINFYNDISLNPQIIEQAVMIPQNVHRILINLMKYLQKWKRYRPLWKLDKAIVMEKFAAKKPPCVAYDEKLQFYSKIAYEVMRHPLIKDEHCIRLQLRHLANTVQENAKSWVISLGKLLNES
AKEELYNLHEEMEHLAKNLRKIPNTLEDLKFVLATIAEIRSKSLVMELRYRDVQERYRTMAMYNLFPPDAEKELVDKIESIWSNLFNDSVNVEHALGDIKRTFTELTRGEIMNYRVQIEEFAKRFYSEGPGSVGDDLDKGVELLGVYERELARHEKSRQELANAEKLFDLPITMYPELLKVQKEMSGLR
MIYELYEGLKVAKEEWSQTLWINLNVQILQEGIEGFLRALRKLPRPVRGLSVTYYLEAKMKAFKDSIPLLLDLKNEALRDRHWKELMEKTSVFFEMTETFTLENMFAMELHKHTDVLNEIVTAAIKEVAIEKAVKEILDTWENMKFTVVKYCKGTQERGYILGSVDEIIQSLDDNTFNLQSISGSRFVG
PFLQTVHKWEKTLSLIGEVIEIWMLVQRKWMYLESIFIGGDIRSQLPEEAKKFDNIDKVFKRIMGETLKDPVIKRCCEAPNRLSDLQNVSEGLEKCQKSLNDYLDSKRNAFPRFFFISDDELLSILGSSDPLCVQEHMIKMYDNIASLRFNDGDSGEKLVSAMISAEGEVMEFRKILRAEGRVEDWMT
AVLNEMRRTNRLITKEAIFRYCEDRSRVDWMLLYQGMVVLAASQVWWTWEVEDVFHKAQKGEKQAMKNYGRKMHRQIDELVTRITMPLSKNDRKKYNTVLIIDVHARDIVDSFIRGSILEAREFDWESQLRFYWDREPDELNIRQCTGTFGYGYEYMGLNGRLVITPLTDRIYLTLTQALSMY
LGGAPAGPAGTGKTETTKDLAKALGLLCVVTNCGEGMDYRAVGKIFSGLAQCGAWGCFDEFNRIDASVLSVISSQIQTIRNALIHQLTTFQFEGQEISLDSRMGIFITMNPGYAGRTELPESVKALFRPVVVIVPDLQQICEIMLFSEGFLEAKTLAKKMTVLYKLAREQLSKQYHYDFGLRALKSVLV
MAGELKRGSSDLREDVVLMRALRDMNLPKFVFEDVPLFLGLISDLFPGLDCPRVRYPDFNDAVEQVLEENGYAVLPIQVDKVVQMFETMLTRHTTMVVGPTRGGKSVVINTLCQAQTKLGLTTKLYILNPKAVSVIELYGILDPTTRDWTDGVLSNIFREINKPTDKKERKYILFDGDVDALWVEN
MNSVMDDNRLLTLANGERIRLQAHCALLFEVGDLQYASPATVSRCGMVYVDPKNLKYRPYWKKWVNQIPNKVEQYNLNSLFEKYVPYLMDVIVEGIVDGRQAEKLKTIVPQTDLNMVTQLAKMLDALLEGEIEDLDLLECYFLEALYCSLGASLLEDGRMKFDEYIKRLASLSTVDTEGVWANP
GELPGQLPTLYDFHFDNKRNQWVPWSKLVPEYIHAPERKFINILVHTVDTTRTTWILEQMVKIKQPVIFVGESGTSKTATTQNFLKNLSEETNIVLMVNFSSRTTSMDIQRNLEANVEKRTKDTYGPPMGKRLLVFMDDMNMPRVDEYGTQQPIALLKLLLEKGYLYDRGKELNCKSIRDLGFIAA
MGKAGGGRNEVDPRFISLFSVFNVPFPSEESLHLIYSSILKGHTSTFHESIVAVSGKLTFCTLALYKNIVQDLPPTPSKFHYIFNLRDLSRVFNGLVLTNPERFQTVAQMVRVWRNECLRVFHDRLISETDKQLVQQHIGSLVVEHFKDDVEVVMRDPILFGDFQMALHEGEPRIYEDIQDYEAAKALF
QEILEEYNESNTKMNLVLFDDALEHLTRVHRIIRMDRGHALLVGVGGSGKQSLSRLAAFTASCEVFEILLSRGYSENSFREDLKSLYLKLGIENKAMIFLFTDAHVAEEGFLELINNMLTSGIVPALFSEEEKESILSQIGQEALKQGMGPAKESVWQYFVNKSANNLHIVLGMSPVGDTLRTWCRNFP
GMVNNTGIDWFMPWPPQALHAVAKSFLGYNPMIPAENIENVVKHVVLVHQSVDHYSQQFLQKLRRSNYVTPKNYLDFINTYSKLLDEKTQCNIAQCKRLDGGLDKLKEATIQLDELNQKLAEQKIVLAEKSAACEALLEEIAVNTAVAEEKKKLAEEKAMEIEEQNKVIAMEKAEAETTLAEVMPI
LEAAKLELQKLDKSDVTEIRSFAKPPKQVQTVCECILIMKGYKELNWKTAKGVMSDPNFLRSLMEIDFDSITQSQVKNIKGLLKTLNTTTEEMEAVSKAGLGMLKFVEAVMGYCDVFREIKPKREKVARLERNFYLTKRELERIQNELAAIQKELETLGAKYEAAILEKQKLQEEAEIMERRLIAADKLIS
GLGSENIRWLNDLDELMHRRVKLLGDCLLCAAFLSYEGAFTWEFRDEMVNRIWQNDILEREIPLSQPFRLESLLTDDVEISRWGSQGLPPDELSVQNGILTTRASRFPLCIDPQQQALNWIKRKEEKNNLRVASFNDPDFLKQLEMSIKYGTPFLFRDVDEYIDPVIDNVLEKNIKVSQGRQFIILGDK
EVDYDSNFRLYLNTKLANPRYSPSVFGKAMVINYTVTLKGLEDQLLSVLVAYERRELEEQREHLIQETSENKNLLKDLEDSLLRELATSTGNMLDNVDLVHTLEETKSKATEVSEKLKLAEKTALDIDRLRDGYRPAARRGAILFFVLSEMALVNSMYQYSLIAFLEVFRLSLKKSLPDSILMKRLRNIMDT
LTFSIYNHGCTGLFERHKLLFSFNMTIKIEQAEGRVPQEELDFFLKGNISLEKSKRKKPCAWLSDQGWEDIILLSEMFSDNFGQLPDDVENNQTVWQEWYDLDSLEQFPVPLGYDNNITPFQKLLILRCFRVDRVYRAVTDYVTVTMGEKYVQPPMISFEAIFEQSTPHSPIVFILSPGSDPATDLMKL
AERSGFGGNRLKFLAMGQGQEKVALQLLETAVARGQWLMLQNCHLLVKWLKDLEKSLERITKPHPDFRLWLTTDPTKGFPIGILQKSLKVVTEPPNGLKLNMRATYFKISHEMLDQCPHPAFKPLVYVLAFFHAVVQERRKFGKIGWNVYYDFNESDFQVCMEILNTYLTKAFQQRDPRIPWGS
LKYLIGEVMYGGRAIDSFDRRILTIYMDEYLGDFIFDTFQPFHFFRNKEVDYKIPVGDEKEKFVEAIEALPLANTPEVFGLHPNAEIGYYTQAARDMWAHLLELQPQTGESSSGISRDDYIGQVAKEIENKMPKVFDLDQVRKRLGTGLSPTSVVLLQELERFNKLVVRMTKSLAELQRALAGEVGMS
NELDDVARSLFIGHIPNIWRRLAPDTLKSLGNWMVYFLRRFSQYMLWVTESEPSVMWLSGLHIPESYLTALVQATCRKNGWPLDRSTLFTQVTKFQDADEVNERAGQGCFVSGLYLEGADWDIEKGCLIKSKPKVLVVDLPILKIIPIEAHRLKLQNTFRTPVYTTSMRRNAMGVGLVFEADLFTT
RHISHWVLQGVCLTLNSD

DNAH10-210, 514,840.64 g/mol; length 4,471 aa [Unique peptide at aa 1487-1514]

>sp|Q8IVF4|DYH10_HUMAN Dynein heavy chain 10, axonemal OS=Homo sapiens OX=9606 GN=DNAH10 PE=1 SV=4
MVPEEVEVEIDEIPVLSEEGEEEEETYSQKVESVDKVRAKRVSLRTESLGQPLNREDEEMDKEISEKLPSKRTAKHIMEKMHLHMLCTPLPEEFLDQNVVFFLRNTKEAISEATDMKEAMEIMPETLEYGIINANVLHFLKNIICQVFLPALSFNQHRTSTTVGVTSGEVSNSSEHESDLPPMPGEAVE
YHSIQLIRDEFLMNVQKFASNIQRTMQQLEGEIKLEMPIISVEGEVSDLAADPETVDILEQCVINWLNQISTAVEAQLKKTPQGKGPLAEIEFWRERNATLSALHEQTKLPIVRKVLDVIKESDSMLVANLQPVFTELFKFHTEASDNVRFLSTVERYFKNITHGSGFHVVLDTIPAMMSALRMVWIIS
RHYNKDERMIPLMERIAWEIAERVCRVVNLRTLFKENRASAQSKTLEARNTLRLWKKAYFDTRAKIEASGREDRWEFDRKRLFERTDYMATICQDLSDVLQILEEFYNIFGPELKAVTGDPKRIDDVLCRVDGLVTPMENLTFDPFSIKSSQFWKYVMDEFKIEVLIDIINKIFVQNLENPPLYKNHPP
VAGAIYWERSLFFRIKHTILRFQEVQEILDSDRGQEVKQKYLEVGRTMKEYEDRKYEQWMEVTEQVLPALMKKSLLTKSSIATEEPSTLERGAVFAINFSPALREIINETKYLEQLGFTVPELARNVALQEDKFLRYTAGIQRMLDHYHMLIGTLNDAESVLLKDHSQELLRVFRSGYKRLNWNSLGIG
DYITGCKQAIGKFESLVHQIHKNADDISSRLTLIEAINLFKYPAAKSEEELPGVKEFFEHIERERASDVDHMVRWYLAIGPLLTKVEGLVVHTNTGKAPKLASYYKYWEKKIYEVLTKLILKNLQSFNSLILGNVPLFHTETILTAPEIILHPNTNEIDKMCFHCVRNCVEITKHFVRWMNGSCIECPPQKGE
EEEVVIINFYNDISLNPQIIEQAVMIPQNVHRILINLMKYLQKWKRYRPLWKLDKAIVMEKFAAKKPPCVAYDEKLQFYSKIAYEVMRHPLIKDEHCIRLQLRHLANTVQENAKSWVISLGKLLNESAKEELYNLHEEMEHLAKNLRKIPNTLEDLKFVLATIAEIRSKSLVMELRYRDVQERYRTMAM
YNLFPPDAEKELVDKIESIWSNLFNDSVNVEHALGDIKRTFTELTRGEIMNYRVQIEEFAKRFYSEGPGSVGDDLDKGVELLGVYERELARHEKSRQELANAEKLFDLPITMYPELLKVQKEMSGLRMIYELYEGLKVAKEEWSQTLWINLNVQILQEGIEGFLRALRKLPRPVRGLSVTYYLEAKMKA
FKDSIPLLLDLKNEALRDRHWKELMEKTSVFFEMTETFTLENMFAMELHKHTDVLNEIVTAAIKEVAIEKAVKEILDTWENMKFTVVKYCKGTQERGYILGSVDEIIQSLDDNTFNLQSISGSRFVGPFLQTVHKWEKTLSLIGEVIEIWMLVQRKWMYLESIFIGGDIRSQLPEEAKKFDNIDKVFKRI
MGETLKDPVIKRCCEAPNRLSDLQNVSEGLEKCQKSLNDYLDSKRNAFPRFFFISDDELLSILGSSDPLCVQEHMIKMYDNIASLRFNDGDSGEKLVSAMISAEGEVMEFRKILRAEGRVEDWMTAVLNEMRRTNRLITKEAIFRYCEDRSRVDWMLLYQGMVVLAASQVWWTWEVEDVFHKA
QKGEKQAMKNYGRKMHRQIDELVTRITMPLSKNDRKKYNTVLIIDVHARDIVDSFIRGSILEAREFDWESQLRFYWDREPDELNIRQCTGTFGYGYEYMGLNGRLVITPLTDRIYLTLTQALSMYLGGAPAGPAGTGKTETTKDLAKALGLLCVVTNCGEGMDYRAVGKIFSGLAQCGAWGCFDE
FNRIDASVLSVISSQIQTIRNALIHQLTTFQFEGQEISLDSRMGIFITMNPGYAGRTELPESVKALFRPVVVIVPDLQQICEIMLFSEGFLEAKTLAKKMTVLYKLAREQLSKQYHYDFGLRALKSVLVMAGELKRGSSDLREDVVLMRALRDMNLPKFVFEDVPLFLGLISDLFPGLDCPRVRYPDFNDA
VEQVLEENGYAVLPIQVDKVVQMFETMLTRHTTMVVGPTRGGKSVVINTLCQAQTKLGLTTKLYILNPKAVSVIELYGILDPTTRDWTDGVLSNIFREINKPTDKKERKYILFDGDVDALWVENMNSVMDDNRLLTLANGERIRLQAHCALLFEVGDLQYASPATVSRCGMVYVDPKNLKYRPY
WKKWVNQIPNKVEQYNLNSLFEKYVPYLMDVIVEGIVDGRQAEKLKTIVPQTDLNMVTQLAKMLDALLEGEIEDLDLLECYFLEALYCSLGASLLEDGRMKFDEYIKRLASLSTVDTEGVWANPGELPGQLPTLYDFHFDNKRNQWVPWSKLVPEYIHAPERKFINILVHTVDTTRTTWILEQMVK
IKQPVIFVGESGTSKTATTQNFLKNLSEETNIVLMVNFSSRTTSMDIQRNLEANVEKRTKDTYGPPMGKRLLVFMDDMNMPRVDEYGTQQPIALLKLLLEKGYLYDRGKELNCKSIRDLGFIAAMGKAGGGRNEVDPRFISLFSVFNVPFPSEESLHLIYSSILKGHTSTFHESIVAVSGKLTFCTLALY
KNIVQDLPPTPSKFHYIFNLRDLSRVFNGLVLTNPERFQTVAQMVRVWRNECLRVFHDRLISETDKQLVQQHIGSLVVEHFKDDVEVVMRDPILFGDFQMALHEGEPRIYEDIQDYEAAKALFQEILEEYNESNTKMNLVLFDDALEHLTRVHRIIRMDRGHALLVGVGGSGKQSLSRLAAFTASC
EVFEILLSRGYSENSFREDLKSLYLKLGIENKAMIFLFTDAHVAEEGFLELINNMLTSGIVPALFSEEEKESILSQIGQEALKQGMGPAKESVWQYFVNKSANNLHIVLGMSPVGDTLRTWCRNFPGMVNNTGIDWFMPWPPQALHAVAKSFLGYNPMIPAENIENVVKHVVLVHQSVDHYSQQF

LQKLRRSNYVTPKNYLDFINTYSKLLDEKTQCNIAQCKRLDGGLDKLKEATIQLDELNQKLAEQKIVLAEKSAACEALLEEIAVNTAVAEEKKKLAEEKAMEIEEQNKVIAMEKAEAETTLAEVMPILEAAKLELQKLDKSDVTEIRSFAKPPKQVQTVCECILIMKGYKELNWKTAKGVMSDPNFLRSL
MEIDFDSITQSQVKNIKGLLKTLNTTTEEMEAVSKAGLGMLKFVEAVMGYCDVFREIKPKREKVARLERNFYLTKRELERIQNELAAIQKELETLGAKYEAAILEKQKLQEEAEIMERRLIAADKLISGLGSENIRWLNDLDELMHRRVKLLGDCLLCAAFLSYEGAFTWEFRDEMVNRIWQNDILEREI
PLSQPFRLESLLTDDVEISRWGSQGLPPDELSVQNGILTTRASRFPLCIDPQQQALNWIKRKEEKNNLRVASFNDPDFLKQLEMSIKYGTPFLFRDVDEYIDPVIDNVLEKNIKVSQGRQFIILG DKEVDYDSNFRLYLNTKLANPRYSPSVFGKAMVINYTVTLKGLEDQLLSVLVAYERRELEEQREHL
IQETSENKNLLKDLEDSLLRELATSTGNMLDNVDLVHTLEETKSKATEVSEKLKLAEKTALDIDRLRDGYRPAARRGAILFFVLSEMALVNSMYQYSLIAFLEVFRLSLKKSLPDSILMKRLRNIMDTLTFSIYNHGCTGLFERHKLLFSFNMTIKIEQAEGRVPQEELDFFLKGNISLEKSKRKKPCAWLSD
QGWEDIILLSEMFSDNFGQLPDDVENNQTVWQEWYDLDSLEQFPVPLGYDNNITPFQKLLILRCFRVDRVYRAVTDYVTVTMGEKYVQPPMISFEAIFEQSTPHSPIVFILSPGSDPATDLMKLAERSGFGGNRLKFLAMGQGQEKVALQLLETAVARGQWLMLQNCHLLVKWLKDLEKSLERI
TKPHPDFRLWLTTDPTKGFPIGILQKSLKVVTEPPNGLKLNMRATYFKISHEMLDQCPHPAFKPLVYVLAFFHAVVQERRKFGKIGWNVYYDFNESDFQVCMEILNTYLTKAFQQRDPRIPWGSLKYLIGEVMYGGRAIDSFDRRILTIYMDEYLGDFIFDTFQPFHFFRNKEVDYKIPVGDEKEKFV
EAIEALPLANTPEVFGLHPNAEIGYYTQAARDMWAHLLELQPQTGESSSGISRDDYIGQVAKEIENKMPKVFDLDQVRKRLGTGLSPTSVVLLQELERFNKLVVRMTKSLAELQRALAGEVGMSNELDDVARSLFIGHIPNIWRRLAPDTLKSLGNWMVYFLRRFSQYMLWVTESEPSVMWLSG
LHIPESYLTALVQATCRKNGWPLDRSTLFTQVTKFQDADEVNERAGQGCFVSGLYLEGADWDIEKGCLIKSKPKVLVVDLPILKIIPIEAHRLKLQNTFRTPVYTTSMRRNAMGVGLVFEADLFTTRHISHWVLQGVCLTLNSD

Table S7. Differentially expressed dynein and kinesin genes in psoriatic lesional and non-lesional skin. Expression of dynein and kinesin genes in psoriatic skin analyzed in the previous RNA sequencing studies (Jabbari et al. 2012; Li et al. 2014; Tervaniemi et al. 2016). Fold-change values are from the comparisons between the lesional skin and with the non-lesional skin. Red color, up-regulation; blue color, down-regulation.

	Dynein gene expression (fold-change)		
	Tervaniemi	*Li*	*Jabbari*
DNAH5	-	0.59	0.66
DNAH6	-	0.46	-
DNAH7	-	0.51	0.56
DNAH8	-	0.11	-
DNAH9	-	0.51	-
DNAH10	-	0.48	-
DNAH11	-	0.66	0.47
DNAH14	-	0.65	0.62
DNAH17	-	0.66	-
DNAI1	-	0.00	0.30
DNALI1	-	0.55	-
DNAL1	-	0.71	-
DNAL4	-	0.67	-
DYNC1I1	-	0.45	-
DYNC1I2	1.56	-	-
DYNC2H1	-	0.64	-
DYNC2LI1	-	0.69	-
DYNLL1	1.89	1.34	-
DYNLRB1	-	0.61	-
DYNLRB2	-	0.36	-
DYNLT1	1.82	1.32	-
DYNLT3	2.05	-	-

	Kinesin gene expression (fold-change)		
	Tervaniemi	*Li*	*Jabbari*
KIF1B	1.78	-	-
KIFC1	-	2.26	-
KIFC3	2.42	-	-
KIF2A	-	1.49	-
KIF2C	5.64	3.12	-
KIF4A	-	3.07	-
KIF4B	-	2.87	3.16
KIF5A	-	0.54	-
KIF5C	-	0.58	-
KIF6	-	0.40	0.54
KIF7	-	0.62	-
KIF11	-	2.32	0.47
KIF12	-	0.32	-
KIF13B	2.37	-	-
KIF14	-	3.33	-
KIF15	-	2.12	1.63
KIF17	-	0.50	
KIF18A	-	2.97	1.79
KIF18B	-	3.16	-
KIF19	-	0.44	-
KIF20A	-	3.86	-
KIF20B	3.60	1.77	-
KIF21A	-	0.48	-
KIF21B	-	1.45	1.94
KIF23	4.02	2.58	-
KIF24	-	1.71	-
KIF26A	-	0.39	-
KIF27	-	0.69	0.62

Table S8. Patient demographics in immunohistochemistry. In the gray background are presented those patients whose samples were used for DNAH10 and CASP14, all for MIB-1 and Collagen type IV stainings.

Gender	Age (years)	TBSA %
Male	39	30
Female	58	43
Male	54	40
Male	41	42
Male	32	45
Male	19	22
Male	37	39
Male	25	27
All mean	38 ± 13	36 ± 8 %
Subgroup mean	48 ± 9	39 ± 6 %
